# Supplementary material for: High-throughput 3D engineered paediatric tumour models for precision medicine
Source: Mol Syst Biol. 2025 Oct 1;21(12):1748–77. doi: 10.1038/s44320-025-00152-y (PMC12673126; doi:10.1038/s44320-025-00152-y)
Supplement: Supplementary file 1 — Table EV1 [file 44320_2025_152_MOESM1_ESM.docx]

# Table EV1 Patient demographics and patient-derived sample information.

| **Cancer Type** | **Sample ID** | **Source** | **Sex/Age (years)** | **Sample Type** | **Key molecular characteristics** |
| --- | --- | --- | --- | --- | --- |
| **Neuroblastoma** | zccs154 | PDX | M/7 | Progression | *MYCN* amplification; *ALK* focal amplification |
|  | zccs373 | PDX | M/1 | Relapse | *MYCN* amplification |
| **Ewing Sarcoma** | zccs59 | PDX | F/12 | Relapse | Somatic *PIK3CA* mutation;  *EWSR1-ETV1* fusion |
|  | zccs207 | PDX | F/14 | Diagnostic | Somatic *TP53* mutation; *TP53* gain;  *EWSR1-FL1* fusion |
|  | zccs227 | PDX | M/5 | Relapse | *EWSR1 – ERG* fusion |
|  | zccs1035 | Cryopreserved direct patient | M/11 | Relapse | Somatic *SETD2, STAG2* and *SDHA* mutation*;*  *EWSR1 – FLI* fusion |
|  | zccs486 | Cryopreserved direct patient | M/10 | Diagnosis | *TP53* mutation*; EWSR1 – ERG* fusion |
| **Osteosarcoma** | zccs43 | PDX | F/15 | Diagnostic | *TP53 - LSMD1* duplication |
|  | zccs225 | PDX | F/17 | Relapse | *NUDT2 -TP53* fusion;  *DLG2-DLG2* deletion |
|  | zccs265 | PDX | F/18 | Relapse | *RB1* germline mutation; *DLG2- DLG2* segmental deletion;  *TP53-TP53* segmental biallelic deletion |
