# Supplementary material for: High-throughput 3D engineered paediatric tumour models for precision medicine
Source: Mol Syst Biol. 2025 Oct 1;21(12):1748–77. doi: 10.1038/s44320-025-00152-y (PMC12673126; doi:10.1038/s44320-025-00152-y)
Supplement: Supplementary file 2 — Table EV2 [file 44320_2025_152_MOESM2_ESM.docx]

# Table EV2 Success rates for generating cultures from primary patient-derived cells (fresh or cryopreserved)

| **Cancer Type** | **Attempted cultures for unique ZERO* samples** | **Samples with successful cultures** | **Success (%)** |
| --- | --- | --- | --- |
| **Neuroblastoma** | 10 | 0 | 0 |
| **Ewing Sarcoma** | 21 | 1 | 4.8 |
| **Osteosarcoma** | 23 | 3 | 13 |

* ZERO Childhood Cancer Precision Medicine Program
