# Supplementary material for: High-throughput 3D engineered paediatric tumour models for precision medicine
Source: Mol Syst Biol. 2025 Oct 1;21(12):1748–77. doi: 10.1038/s44320-025-00152-y (PMC12673126; doi:10.1038/s44320-025-00152-y)
Supplement: Supplementary file 3 — Table EV3 [file 44320_2025_152_MOESM3_ESM.docx]

# Table EV3 In vivo expansion time for individual patient-derived samples, related to Figure 2.

| **Cancer Type** | **Sample ID (ZERO*)** | **Time**  **(days**)** |
| --- | --- | --- |
| **Neuroblastoma** | zccs154 | >70 |
|  | zccs373 | >88 |
| **Ewing Sarcoma** | zccs59 | >57 |
| **Osteosarcoma** | zccs43 | >98 |
|  | zccs265 | >179 |

* ZERO Childhood Cancer Precision Medicine Program

**In vivo expansion time was only available for five out of eight patient samples
