# Supplementary material for: High-throughput 3D engineered paediatric tumour models for precision medicine
Source: Mol Syst Biol. 2025 Oct 1;21(12):1748–77. doi: 10.1038/s44320-025-00152-y (PMC12673126; doi:10.1038/s44320-025-00152-y)
Supplement: Supplementary file 4 — Table EV4 [file 44320_2025_152_MOESM4_ESM.docx]

# Table EV4 Comparative analysis of genetic variants in original patient tumours by whole genome sequencing and 3D tumouroids by targeted sequencing (TSO500), related to Figure 3.

| \| **Disease** \| **Patient** \| **Gene** \| **Variation** \| **Description** \| **Status** \| **Original patient sample** \| \| \| **Bioprinted sample** \| \| \| \| --- \| --- \| --- \| --- \| --- \| --- \| --- \| --- \| --- \| --- \| --- \| --- \| \| **Whole Genome Sequencing (WGS)** \| **Copy number** \| **Variant allele frequency (VAF)** \| **TruSight 500 (TSO500)** \| **Copy number** \| **Variant allele frequency (VAF)** \| \| **Neuroblastoma** \| **zccs373** \| *MYCN* \| Somatic copy number variant \| CNV \| pathogenic \| Yes \| 201.51 \|  \| Yes \| 73.6 \|  \| \| **zccs154** \| *MYCN* \| Somatic copy number variant \| CNV \| pathogenic \| Yes \| 49.82 \|  \| Yes \| 127.5 \|  \| \| *ALK* \| Somatic copy number variant \| CNV \| pathogenic \| Yes \| 66.34 \|  \| Yes \| 80.6 \|  \| \| *ALK-THADA* \| Somatic structural variant (duplication) \| SV \| not pathogenic \| Yes \|  \|  \| Yes \|  \|  \| \| **Ewing Sarcoma** \| **zccs207** \| *EWSR1-FLI1* \| Somatic structural variant  (fusion) \| SV \| pathogenic \| Yes \|  \|  \| Yes \|  \|  \| \| *STAG2* \| Single nucleotide variation  NM_006603.4(STAG2):c.3395T>G (p.Leu1132Ter) \| SNV \| likely pathogenic \| Yes \|  \| 43.24% \| Yes \|  \| 63.10% \| \| *TERT* \| Single nucleotide variation  NM_198253.2(TERT):c.-57A>C \| SNV \| likely pathogenic \| Yes \|  \| 65.09% \| Yes \|  \| 32.35% \| \| *TP53* \| Single nucleotide variation  NM_000546.5(TP53):c.577C>T (p.His193Tyr) \| SNV \| pathogenic \| Yes \|  \| 85.37% \| Yes \|  \| 99.84% \| \| **zccs227** \| *EWSR-ERG* \| Somatic structural variant  (fusion) \| SV \| pathogenic \| Yes \|  \|  \| No \|  \|  \| \| **zccs59** \| *SMARCA4* \| Single nucleotide variation  NM_003072(SMARCA4):c.3469C>T (p.Arg1157Trp) \| SNV \| likely pathogenic \| Yes \|  \| 4.58% \| Yes \|  \| 47.09% \| \| *PIK3CA* \| Single nucleotide variation  NM_006218.4(PIK3CA):c.3140A>G (p.His1047Arg) \| SNV \| ­  pathogenic \| Yes \|  \| 8.09% \| Yes \|  \| 36.40% \| \| *ARID1A* \| Single nucleotide variation  NM_006015(ARID1A):c.3859dupA (p.Arg1287LysfsTer11) \| SNV \| pathogenic \| Yes \|  \| 21.21% \| Yes \|  \| 18% \| \| *EWSR1 - ETV1* \| Somatic structural variant  (fusion) \| SV \| pathogenic \| Yes \|  \|  \| Yes \|  \|  \| \| **Osteosarcoma** \| **zccs225** \| *NUDT21-TP53* \| Somatic structural variant  (fusion) \| SV \| reportable \| Yes \|  \|  \| Yes \|  \|  \| \| **zccs43** \| *TP53-LSMD1* \| Somatic structural variant  (fusion) \| SV \| reportable \| Yes \|  \|  \| No \|  \|  \| \| **zccs265** \| *BCL2* \| Somatic copy number variant \| CNV \| pathogenic \| Yes \| 17.59 \|  \| Yes \| 17.1 \|  \| \| *RB1* \| Single nucleotide variation  NM_000321(RB1):c.507_508dupTG (p.Glu170ValfsTer6) \| SNV \| pathogenic \| Yes \|  \| 99.83% \| Yes \|  \| 97% \| \| *TP53-TP53* \| Structural variant  Segmental biallelic deletion (exon 1) \| SV \| pathogenic \| Yes \|  \|  \| No \|  \|  \| |
| --- | --- | --- | --- | --- | --- | --- | --- | --- | --- | --- | --- | --- | --- | --- | --- | --- | --- | --- | --- | --- | --- | --- | --- | --- | --- | --- | --- | --- | --- | --- | --- | --- | --- | --- | --- | --- | --- | --- | --- | --- | --- | --- | --- | --- | --- | --- | --- | --- | --- | --- | --- | --- | --- | --- | --- | --- | --- | --- | --- | --- | --- | --- | --- | --- | --- | --- | --- | --- | --- | --- | --- | --- | --- | --- | --- | --- | --- | --- | --- | --- | --- | --- | --- | --- | --- | --- | --- | --- | --- | --- | --- | --- | --- | --- | --- | --- | --- | --- | --- | --- | --- | --- | --- | --- | --- | --- | --- | --- | --- | --- | --- | --- | --- | --- | --- | --- | --- | --- | --- | --- | --- | --- | --- | --- | --- | --- | --- | --- | --- | --- | --- | --- | --- | --- | --- | --- | --- | --- | --- | --- | --- | --- | --- | --- | --- | --- | --- | --- | --- | --- | --- | --- | --- | --- | --- | --- | --- | --- | --- | --- | --- | --- | --- | --- | --- | --- | --- | --- | --- | --- | --- | --- | --- | --- | --- | --- | --- | --- | --- | --- | --- | --- | --- | --- | --- | --- | --- | --- | --- | --- | --- | --- | --- | --- | --- | --- | --- | --- | --- | --- | --- | --- | --- | --- | --- | --- | --- | --- | --- |
