# Supplementary material for: High-throughput 3D engineered paediatric tumour models for precision medicine
Source: Mol Syst Biol. 2025 Oct 1;21(12):1748–77. doi: 10.1038/s44320-025-00152-y (PMC12673126; doi:10.1038/s44320-025-00152-y)
Supplement: Supplementary file 5 — Table EV5 [file 44320_2025_152_MOESM5_ESM.docx]

# Table EV5. Integrated analysis of the original tumour, PDX and tumouroids from WGS, TSO500 and RNAseq assays for validation of key molecular characteristics. Related to Figure 3.

| **Disease** | **Patient** | **Gene** | **Variation** | **Description** | **Status** | **Original Patient Sample** | | | **PDX Bioprinted Sample** | | | | | | | **PDX RNAseq** | | | |
| --- | --- | --- | --- | --- | --- | --- | --- | --- | --- | --- | --- | --- | --- | --- | --- | --- | --- | --- | --- |
|  |  |  |  |  |  | **WGS** | **CNV** | **SNV VAF** | **TSO500** | **RNA** | **CNV** | **Expression** | **SV** | **TSO500 SNV VAF** | **RNA SNV VAF** | **RNA** | **Expression** | **SV** | **RNA SNV VAF** |
| NB | zccs373 | MYCN |  | CNV | pathogenic | Y | 201.51 |  | Y | Y | 73.6 | 1803.48 |  |  |  | Y | 1399.05 |  |  |
| EWS | zccs207 | EWSR1::FLI1 |  | SV | pathogenic | Y |  |  | Y | Y |  |  | high in-frame |  |  | Y |  | high in-frame |  |
|  |  | STAG2 | NM_006603.4(STAG2):c.3395T>G (p.Leu1132Ter) | SNV | likely pathogenic | Y |  | 43.24% | Y | Y |  |  |  | 63.10% | 98.70% | Y |  |  | 99.30% |
|  |  | TERT | NM_198253.2(TERT):c.-57A>C | SNV | likely pathogenic | Y |  | 65.09% | Y | Y |  | 2 |  | 32.35% | Increased Expression from Promoter Mutation | Y | 3.78 |  | Increased Expression from Promoter Mutation |
|  |  | TP53 | NM_00546.5(TP53):c.577C>T (p.His193Tyr) | SNV | pathogenic | Y |  | 85.37% | Y | Y |  |  |  | 99.84% | 100% | Y |  |  | 100% |
| OST | zccs225 | NUDT21::TP53 |  | SV | reportable? | Y |  |  | Y | Y |  |  | high in-frame |  |  | Y |  | high in-frame |  |

NB = Neuroblastoma

EWS = Ewing Sarcoma

OST = Osteosarcoma

Y = Yes

WGS = Whole Genome Sequencing

CNV = Copy Number Variant

SNV VAF = Single Nucleotide Variation Variant Allele Frequency

SV = Structural Variant
