# Supplementary material for: High-throughput 3D engineered paediatric tumour models for precision medicine
Source: Mol Syst Biol. 2025 Oct 1;21(12):1748–77. doi: 10.1038/s44320-025-00152-y (PMC12673126; doi:10.1038/s44320-025-00152-y)
Supplement: Supplementary file 6 — Table EV6 [file 44320_2025_152_MOESM6_ESM.docx]

# Table EV6. Drug library information, related to Figure 6.

| **Drug** | **Synonyms** | **Category** | **Drug Class** | **Target(s)** |
| --- | --- | --- | --- | --- |
| Afuresertib | GSK-2110183; GSK2110183 | Targeted | PI3K-AKT-mTOR signalling | AKT1; AKT2; AKT3 |
| Alectinib | Alecensa; RG7853; AF802; CH5424802; CH 5424802; RO5424802 | Targeted | Receptor tyrosine kinase signalling | ALK |
| Alisertib | MLN8237 | Targeted | Aurora-PLK signalling | AURKA |
| Alpelisib | BYL-719; BYL719 | Targeted | PI3K-AKT-mTOR signalling | PI3Ka |
| Bleomycin | Bleomycin sulfate; Blexane; NSC125066; Blenoxane | Chemotherapeutic | DNA structure and function | Free radical-promoting agent |
| Buparlisib | BKM120; NVP-BKM120 | Targeted | PI3K-AKT-mTOR signalling | PI3Ka; PI3Kb; PI3Kd; PI3Kg |
| Busulfan | Myleran; Busulphan; Sulphabutin; Myelosan; Leucosulfan; Busulfex | Chemotherapeutic | DNA synthesis | DNA dialkylating agent (N7-guanine residues) |
| Cabozantinib | XL184; XL-184; BMS-907351; Cometriq | Targeted | Receptor tyrosine kinase signalling | c-Met; VEGFR2; AXL; RET; c-Kit; Flt3 |
| Carboplatin | NSC 241240 | Chemotherapeutic | DNA structure and function | DNA crosslinker |
| Carfilzomib | PR-171 | Targeted | Proteasome function | Proteasome |
| Ceritinib | LDK378 | Targeted | Receptor tyrosine kinase signalling | ALK |
| Crenolanib | CP-868596 | Targeted | Receptor tyrosine kinase signalling | PDGFRa; PDGFRb; Flt3 |
| Crizotinib | PF-02341066; PF02341066; PF 02341066; Xalkori | Targeted | Receptor tyrosine kinase signalling | c-Met; ALK; ROS1 |
| Dabrafenib | GSK2118436 | Targeted | MAPK-ERK signalling | BRAFV600E |
| Dactinomycin | Actinomycin D | Chemotherapeutic | RNA synthesis | dsDNA intercalator |
| Dinaciclib | SCH727965; SCH-727965; SCH 727965; MK-7965; PS-095760 | Targeted | Cell cycle-checkpoint signalling | CDK1; CDK2; CDK5; CDK9 |
| Doxorubicin | Doxorubicin hydrochloride; Adriamycin; Adriacin; Adriblastina; Adriblastin | Chemotherapeutic | DNA replication | DNA topoisomerase II inhibitor |
| Epirubicin | Epirubicin hydrochloride; 4'-epidoxorubicin hydrochloride | Chemotherapeutic | DNA replication | DNA topoisomerase II inhibitor |
| Fexagratinib | AZD4547 | Targeted | Receptor tyrosine kinase signalling | FGFR1; FGFR2; FGFR3; FGFR4; KDR |
| Gefitinib | ZD1839; ZD-1839 | Targeted | Receptor tyrosine kinase signalling | EGFR |
| Gemcitabine | Gemcitabine hydrochloride; Gemzar; LY-188011 | Chemotherapeutic | Nucleic acid synthesis or utilization | Antimetabolite (pyrimidine analogue) |
| GENZ-644282 | genz644282; Genz 644282 | Chemotherapeutic | DNA replication | DNA topoisomerase I inhibitor |
| Irinotecan | Irinotecan hydrochloride; (+)-Irinotecan; CPT-11 | Chemotherapeutic | DNA replication | DNA topoisomerase I inhibitor |
| Lapatinib | Tyverb; GW-572016, GSK572016; | Targeted | Receptor tyrosine kinase signalling | EGFR; HER2 |
| Larotrectinib | Larotrectinib sulfate; LOXO-101; ARRY-470 | Targeted | Receptor tyrosine kinase signalling | TrkA; TrkB; TrkC |
| Lomustine | CCNU | Chemotherapeutic | DNA structure and function | DNA alkylating agent (O6/N7-guanine residues) |
| Melphalan | Melphalan hydrochloride; Alkeran; Sarcolysin; L-PAM | Chemotherapeutic | DNA structure and function | DNA alkylating agent (N7-guanine residues) |
| Mitomycin C | Ametycine | Chemotherapeutic | DNA structure and function | DNA alkylating agent (N2-guanine residues) |
| Nintedanib | BIBF 1120; Intedanib | Targeted | Receptor tyrosine kinase signalling | VEGFR1; VEGFR2; VEGFR3; FGFR1; FGFR2; FGFR3; PDGFRa; PDGFRb |
| Palbociclib | PD0332991; PD-0332991; PF-00080665-73 | Targeted | Cell cycle-checkpoint signalling | CDK4; CDK6 |
| Panobinostat | LBH589 | Targeted | Epigenetic regulation | pan-HDAC |
| Paxalisib | GDC-0084; RG7666 | Targeted | PI3K-AKT-mTOR signalling | PI3Ka; PI3Kb; PI3Kd; PI3Kg; mTOR |
| Pinometostat | EPZ-5676; EPZ5676; EPZ 5676 | Targeted | Epigenetic regulation | DOT1L |
| PRI-724 |  | Targeted | Wnt signalling | bCatenin-CBP |
| Regorafenib | BAY 73-4506; Stivarga | Targeted | Multitargeted | VEGFR1; VEGFR2; VEGFR3; PDGFRb; c-Kit; RET; Raf-1 |
| Ruxolitinib | INCB018424; INCB-18424; Jakafi | Targeted | JAK-STAT signalling | JAK1; JAK2 |
| SN-38 |  | Chemotherapeutic | DNA replication | DNA topoisomerase I inhibitor |
| Sorafenib | BAY 43-9006; Nexavar; 284461-73-0 | Targeted | Multitargeted | Raf-1; B-Raf; VEGFR-2 |
| Talazoparib | BMN 673; BMN-673; LT-673 | Targeted | DNA damage signalling | PARP1; PARP2 |
| Temozolomide | TMZ; Temodar; Temodal; Temcad; SCH 52365 | Chemotherapeutic | DNA structure and function | DNA alkylating agent (O6/N7-guanine residues) |
| Thiotepa | Thio-TEPA; thioplex; tiofosfamid | Chemotherapeutic | DNA structure and function | DNA alkylating agent (N7-guanine residues) |
| Topotecan | Topotecan hydrochloride; K&F 104864-A; SKF 104864A; SKFS 104864A; NSC 609669; nogitecan hydrochloride | Chemotherapeutic | DNA replication | DNA topoisomerase I inhibitor |
| Trametinib | GSK-1120212; GSK1120212; JTP-74057; Mekinist | Targeted | MAPK-ERK signalling | MEK1; MEK2 |
| Vemurafenib | RG7204; R7204; RO5185426; PLX4032 | Targeted | MAPK-ERK signalling | BRAFV600E |
| Venetoclax | ABT199; ABT-199; GDC-0199 | Targeted | Apoptotic signalling | BCL-2 |
| Vincristine | Vincristine sulfate; Leurocristine sulfate; 22-oxovincaleukoblastine sulfate | Chemotherapeutic | Mitosis | Microtubule destabiliser |
| Volasertib | BI6727; BI 6727; BI-6727 | Targeted | Aurora-PLK signalling | PLK1 |
| Voxtalisib | SAR 245409; SAR245409; XL765; XL-765 | Targeted | PI3K-AKT-mTOR signalling | PI3Ka; PI3Kb; PI3Kd; PI3Kg; DNA-PK; mTORC1; mTORC2 |
