# Supplementary material for: High-throughput 3D engineered paediatric tumour models for precision medicine
Source: Mol Syst Biol. 2025 Oct 1;21(12):1748–77. doi: 10.1038/s44320-025-00152-y (PMC12673126; doi:10.1038/s44320-025-00152-y)
Supplement: Supplementary file 7 — Table EV7 [file 44320_2025_152_MOESM7_ESM.docx]

# Table EV7. AUC and IC50 values for ZERO vs 3D bioprinting HTP drug screen.

|  | **zccs373** | | | | **zccs207** | | | | **zccs225** | | | |
| --- | --- | --- | --- | --- | --- | --- | --- | --- | --- | --- | --- | --- |
|  | **ZERO** | | **3D bioprinting** | | **ZERO** | | **3D bioprinting** | | **ZERO** | | **3D bioprinting** | |
| **Drug Name** | **AUC** | **IC50** | **AUC** | **IC50** | **AUC** | **IC50** | **AUC** | **IC50** | **AUC** | **IC50** | **AUC** | **IC50** |
| Thiotepa | 476.82 | 5.00 | 483.42 | 5.00 | 471.25 | 5.00 | 410.81 | 5.00 | 486.4 | 5.00 | 494.94 | 5.00 |
| Temozolomide | 499.10 | 5.00 | 500.00 | 5.00 | 500.00 | 5.00 | 500.00 | 5.00 | 493.94 | 5.00 | NA | NA |
| Busulfan | 498.27 | 5.00 | 500.00 | 5.00 | 500.00 | 5.00 | 500.00 | 5.00 | 494.37 | 5.00 | NA | NA |
| Lomustine | 500.00 | 5.00 | 483.01 | 5.00 | 500.00 | 5.00 | 493.49 | 5.00 | 475.98 | 5.00 | 500.00 | 5.00 |
| Gemcitabine hydrochloride | 129.06 | 0.00 | 277.01 | 0.03 | 220.95 | 0.01 | 288.43 | 0.06 | 396.31 | 5.00 | NA | NA |
| Mitomycin C | 274.47 | 0.07 | 361.28 | 0.32 | 365.63 | 0.40 | 353.96 | 0.29 | 452.7 | 3.81 | NA | NA |
| Carboplatin | 499.75 | 5.00 | 500.00 | 5.00 | 489.46 | 5.00 | 500.00 | 5.00 | 487.59 | 5.00 | 500.00 | 5.00 |
| Gefitinib | 494.77 | 5.00 | 500.00 | 5.00 | 488.11 | 5.00 | 422.29 | 5.00 | 491.39 | 5.00 | 482.83 | 5.00 |
| Crizotinib | 409.16 | 0.71 | 463.54 | 4.81 | 434.20 | 1.27 | 473.31 | 5.00 | 410.84 | 1.01 | NA | NA |
| Topotecan hydrochloride | 302.67 | 0.08 | 366.42 | 0.20 | 341.33 | 0.17 | 371.44 | 0.10 | 411.25 | 1.30 | 431.13 | 0.73 |
| Sorafenib | 483.75 | 5.00 | 497.55 | 5.00 | 478.77 | 5.00 | 408.06 | 5.00 | 472.66 | 5.00 | NA | NA |
| Vemurafenib | 487.98 | 5.00 | 500.00 | 5.00 | 473.92 | 5.00 | 437.99 | 5.00 | 487.31 | 5.00 | NA | NA |
| Doxorubicin hydrochloride | 405.58 | 0.69 | 462.32 | 4.60 | 452.08 | 3.37 | 375.09 | 2.84 | 457.12 | 5.00 | NA | NA |
| Lapatinib | 464.24 | 5.00 | 484.25 | 5.00 | 467.59 | 5.00 | 500.00 | 5.00 | 460.37 | 5.00 | NA | NA |
| Irinotecan hydrochloride | 420.21 | 1.00 | 442.27 | 2.32 | 269.69 | 0.04 | 373.46 | 0.31 | 476.22 | 5.00 | NA | NA |
| Vincristine sulfate | 242.74 | 0.04 | 351.63 | 0.12 | 281.81 | 0.07 | 353.20 | 0.33 | 362.81 | 0.80 | NA | NA |
| Actinomycin D | 32.45 | 0.00 | 96.22 | 0.00 | 180.11 | 0.00 | 232.07 | 0.01 | 199.12 | 0.00 | NA | NA |
| Bleomycin sulfate | 416.76 | 0.86 | 470.98 | 5.00 | 486.90 | 5.00 | 500.00 | 5.00 | 485.92 | 5.00 | NA | NA |
| Carfilzomib | 226.64 | 0.04 | 353.28 | 0.22 | 255.19 | 0.06 | 294.40 | 0.07 | 316.84 | 0.12 | NA | NA |
| Melphalan | 461.96 | 4.42 | 494.52 | 5.00 | 479.81 | 5.00 | 401.44 | 5.00 | 489.55 | 5.00 | 500.00 | 5.00 |
| Regorafenib | 494.21 | 5.00 | 497.09 | 5.00 | 485.17 | 5.00 | 448.17 | 5.00 | 469.36 | 5.00 | 467.50 | 5.00 |
| Cabozantinib | 500.00 | 5.00 | 500.00 | 5.00 | 474.41 | 5.00 | 483.85 | 5.00 | 462.15 | 5.00 | NA | NA |
| Epirubicin hydrochloride | 471.63 | 5.00 | 500.00 | 5.00 | 484.07 | 5.00 | 492.88 | 5.00 | 491.12 | 5.00 | 500.00 | 5.00 |
| Trametinib | 476.15 | 5.00 | 500.00 | 5.00 | 500.00 | 5.00 | 436.15 | 5.00 | 499.99 | 5.00 | 467.78 | NA |
| Dabrafenib | 450.77 | 3.83 | 464.42 | 4.74 | 469.72 | 5.00 | 500.00 | 5.00 | 490.1 | 5.00 | NA | NA |
| Ruxolitinib | 500.00 | 5.00 | 500.00 | 5.00 | 500.00 | 5.00 | 454.12 | 5.00 | 500 | 5.00 | NA | NA |
| Venetoclax | 274.98 | 0.07 | 351.12 | 0.07 | 487.04 | 5.00 | 403.62 | 5.00 | 492 | 5.00 | NA | NA |
| GENZ-644282 | 127.81 | 0.00 | 275.12 | 0.01 | 134.87 | 0.00 | 194.07 | 0.00 | 255.11 | 0.06 | NA | NA |
| Palbociclib | 448.44 | 5.00 | 487.49 | 5.00 | 460.96 | 5.00 | 499.88 | 5.00 | 490.03 | 5.00 | NA | NA |
| Ceritinib | 383.33 | 0.53 | 388.13 | 0.57 | 364.93 | 0.50 | 357.84 | 0.52 | 353.05 | 0.35 | 366.10 | 0.47 |
| Alisertib | 326.38 | 0.11 | 390.38 | 0.20 | 448.16 | 5.00 | 487.21 | 5.00 | 471.77 | 5.00 | 480.47 | NA |
| Buparlisib | 391.18 | 0.57 | 420.60 | 0.69 | 429.03 | 0.88 | 377.50 | 4.58 | 420.02 | 1.08 | 451.29 | 1.14 |
| Crenolanib | 439.58 | 2.13 | 457.76 | 3.93 | 461.23 | 4.59 | 500.00 | 5.00 | 460.03 | 4.33 | NA | NA |
| Dinaciclib | 208.70 | 0.02 | 222.17 | 0.02 | 320.59 | 0.05 | 345.63 | 0.06 | 308.08 | 0.01 | 279.64 | 0.02 |
| Pinometostat | 500.00 | 5.00 | 500.00 | 5.00 | 500.00 | 5.00 | 458.38 | 5.00 | 492.13 | 5.00 | NA | NA |
| Afuresertib | 477.71 | 5.00 | 488.48 | 5.00 | 458.67 | 5.00 | 387.60 | 5.00 | 415.42 | 1.16 | 460.74 | NA |
| Talazoparib | 279.48 | 0.09 | 357.14 | 0.42 | 316.93 | 0.12 | 371.23 | 0.17 | 472.85 | 5.00 | NA | NA |
| Panobinostat | 228.96 | 0.04 | 304.49 | 0.06 | 322.50 | 0.17 | 318.77 | 0.14 | 288.79 | 0.09 | 299.16 | 0.07 |
| Volasertib | 222.02 | 0.04 | 261.43 | 0.05 | 251.15 | 0.02 | 318.77 | 0.06 | 321.58 | 0.09 | 343.19 | 0.14 |
| Alectinib | 420.95 | 0.93 | 436.02 | 4.10 | 451.39 | 5.00 | 473.47 | 5.00 | 469.82 | 5.00 | 480.07 | NA |
| Voxtalisib | 467.84 | 5.00 | 495.35 | 5.00 | 458.26 | 5.00 | 443.74 | 3.29 | 448.01 | 3.67 | 466.92 | 3.95 |
| Alpelisib | 476.74 | 5.00 | 485.13 | 5.00 | 465.35 | 5.00 | 445.92 | 5.00 | 433.65 | 5.00 | NA | NA |
| Nintedanib | 460.25 | 5.00 | 488.39 | 5.00 | 492.07 | 5.00 | 463.88 | 5.00 | 432.46 | 2.33 | 417.15 | 1.83 |
| PRI-724 | 483.21 | 5.00 | 500.00 | 5.00 | 474.75 | 5.00 | 469.45 | 5.00 | 447.8 | 3.61 | 468.14 | 5.00 |
| AZD4547 | 480.29 | 5.00 | 470.00 | 5.00 | 472.02 | 5.00 | 418.65 | 5.00 | 465.93 | 4.80 | 471.05 | 5.00 |
| SN-38 | 83.07 | 0.00 | 224.74 | 0.00 | 126.68 | 0.00 | 185.15 | 0.00 | 338.04 | 0.08 | 350.74 | 0.20 |
| Paxalisib | 413.07 | 0.79 | 479.26 | 5.00 | 404.80 | 1.31 | 453.81 | 1.41 | 392.29 | 0.88 | 398.30 | 0.97 |
| Larotrectinib sulfate | 500.00 | 5.00 | 500.00 | 5.00 | 500.00 | 5.00 | 413.86 | 5.00 | 478.74 | 5.00 | 494.74 | 5.00 |

NA = Not available
