# Supplementary material for: High-throughput 3D engineered paediatric tumour models for precision medicine
Source: Mol Syst Biol. 2025 Oct 1;21(12):1748–77. doi: 10.1038/s44320-025-00152-y (PMC12673126; doi:10.1038/s44320-025-00152-y)
Supplement: Supplementary file 8 — Table EV8 [file 44320_2025_152_MOESM8_ESM.docx]

# Table EV8. Quality control metrics for HTP drug screen

| **Sample ID** | **Disease type** | **Pearson correlation coefficient (r)** | **Robust Z'prime P1** | **Robust Z'prime P2** | **QC status** |
| --- | --- | --- | --- | --- | --- |
| zccs373 | Neuroblastoma | 0.92 | 0.83 | 0.69 | pass |
| zccs154 | Neuroblastoma | 0.82 | 0.66 | 0.55 | pass |
| zccs207 | Ewing Sarcoma | 0.88 | 0.48 | 0.3 | pass |
| zccs227 | Ewing Sarcoma | 0.93 | 0.40 | 0.63 | pass |
| zccs225 | Osteosarcoma | 0.86 | 0.67 | 0.72 | pass |
| zccs265 | Osteosarcoma | 0.85 | 0.78 | 0.83 | pass |
| zccs43 | Osteosarcoma | 0.57 | 0.06 | -0.73 | fail |

P1 = plate 1

P2 = plate 2
