# Supplementary material for: High-throughput 3D engineered paediatric tumour models for precision medicine
Source: Mol Syst Biol. 2025 Oct 1;21(12):1748–77. doi: 10.1038/s44320-025-00152-y (PMC12673126; doi:10.1038/s44320-025-00152-y)
Supplement: Supplementary file 9 — Table EV9 [file 44320_2025_152_MOESM9_ESM.docx]

# Table EV9. AUC and Log2[IC50] T-score values for an individual drug and sample, related to Figure 6.

|  | **AUC T-score** | | | | | | **log2[IC50] T-score** | | | | | |
| --- | --- | --- | --- | --- | --- | --- | --- | --- | --- | --- | --- | --- |
|  | **NB** | **NB** | **EWS** | **EWS** | **OST** | **OST** | **NB** | **NB** | **EWS** | **EWS** | **OST** | **OST** |
| **Drug** | **zccs154** | **zccs373** | **zccs207** | **zccs227** | **zccs225** | **zccs265** | **zccs154** | **zccs373** | **zccs207** | **zccs227** | **zccs225** | **zccs265** |
| Afuresertib | -0.07 | 1.40 | -0.85 | -1.18 | 0.93 | -0.23 | 0.62 | 0.62 | -0.69 | -1.74 | 0.62 | 0.58 |
| Alectinib | -0.95 | 0.87 | -1.39 | 0.29 | 1.15 | 0.03 | -0.49 | -0.54 | -1.44 | 1.07 | 1.07 | 0.32 |
| Alisertib | -0.03 | -1.86 | -0.07 | 0.24 | 0.86 | 0.87 | 0.41 | -2.04 | 0.41 | 0.41 | 0.41 | 0.41 |
| Alpelisib | -0.36 | -0.52 | -1.31 | 1.66 | 0.33 | 0.19 | -0.11 | -0.62 | -1.16 | 1.33 | 1.10 | -0.53 |
| Bleomycin sulfate | 0.05 | 0.67 | -1.04 | -1.32 | 0.40 | 1.25 | -0.20 | 0.54 | 0.54 | -1.95 | 0.54 | 0.54 |
| Buparlisib (BKM120) | 0.76 | -0.21 | 0.02 | -1.85 | 0.37 | 0.90 | 1.32 | -0.19 | -0.42 | -1.53 | -0.01 | 0.84 |
| Busulfan | 0.19 | 1.03 | -0.28 | -1.28 | -0.87 | 1.20 | 0.00 | 0.00 | 0.00 | 0.00 | 0.00 | 0.00 |
| Cabozantinib | -1.17 | 1.06 | -0.10 | -1.21 | 0.85 | 0.57 | 0.42 | 0.42 | 0.42 | -2.04 | 0.42 | 0.34 |
| Carboplatin | -0.31 | 0.70 | 0.18 | -1.88 | 0.62 | 0.70 | 0.41 | 0.41 | 0.41 | -2.04 | 0.41 | 0.41 |
| Carfilzomib | -1.93 | 0.35 | -0.07 | 0.88 | 0.19 | 0.58 | -1.93 | 0.68 | -0.13 | 0.77 | 0.44 | 0.17 |
| Ceritinib | 1.52 | 0.78 | -0.89 | -0.87 | 0.16 | -0.70 | 0.86 | 1.12 | -1.00 | -1.02 | 0.71 | -0.67 |
| Crenolanib | 0.86 | 0.08 | 0.51 | -1.96 | 0.14 | 0.37 | 0.60 | -0.04 | 0.58 | -1.98 | 0.31 | 0.53 |
| Crizotinib | 0.60 | 0.48 | -0.04 | -1.99 | 0.54 | 0.40 | 0.38 | 0.35 | 0.22 | -2.03 | 0.57 | 0.51 |
| Dabrafenib | -0.06 | 0.68 | -0.10 | -1.89 | 0.68 | 0.68 | 0.41 | 0.41 | 0.41 | -2.04 | 0.41 | 0.41 |
| Dactinomycin | -0.13 | -0.98 | -0.49 | 1.93 | -0.09 | -0.25 | -0.40 | -1.12 | 1.24 | 1.24 | -0.35 | -0.62 |
| Dinaciclib | 0.17 | 0.97 | 0.11 | -1.79 | -0.30 | 0.85 | 0.40 | 0.65 | 0.23 | -1.98 | 0.05 | 0.65 |
| Doxorubicin hydrochloride | -0.36 | 0.19 | -1.49 | -0.41 | 0.66 | 1.42 | 0.82 | -0.80 | -1.31 | 0.40 | NA | 0.90 |
| Epirubicin hydrochloride | -0.12 | 0.61 | -1.55 | -0.75 | 0.91 | 0.90 | 0.66 | 0.49 | -0.82 | -1.66 | 0.66 | 0.66 |
| Fexagratinib (AZD4547) | 0.86 | -0.42 | -0.84 | 1.61 | -0.75 | -0.47 | 0.75 | 0.75 | -1.23 | 0.75 | -1.31 | 0.30 |
| Gefitinib | -0.23 | 1.24 | -0.19 | -0.37 | -1.47 | 1.02 | 0.41 | 0.41 | 0.41 | -2.04 | 0.41 | 0.41 |
| Gemcitabine hydrochloride | -0.86 | -1.20 | -0.63 | 0.76 | 0.86 | 1.06 | -0.69 | -1.24 | -0.76 | 0.90 | 0.90 | 0.90 |
| GENZ-644282 | 0.27 | -1.14 | -0.91 | 1.61 | 0.38 | -0.21 | 0.34 | -1.15 | -0.91 | 1.57 | 0.43 | -0.28 |
| Irinotecan hydrochloride | 0.22 | -1.40 | -1.10 | 0.91 | 0.60 | 0.76 | 0.65 | -1.32 | -1.26 | 0.65 | 0.65 | 0.65 |
| Lapatinib | -0.18 | 1.31 | -1.52 | 0.57 | 0.46 | -0.64 | 0.41 | 0.41 | 0.41 | 0.41 | 0.41 | -2.04 |
| Larotrectinib sulfate | -0.76 | 0.33 | -0.90 | -0.52 | 0.05 | 1.80 | 0.41 | 0.41 | 0.41 | -2.04 | 0.41 | 0.41 |
| Lomustine | 0.88 | -0.34 | 0.63 | -1.20 | -1.05 | 1.09 | 0.00 | 0.00 | 0.00 | 0.00 | 0.00 | 0.00 |
| Melphalan hydrochloride | -1.15 | 0.31 | -1.36 | 0.52 | 0.69 | 0.99 | 0.41 | -2.04 | 0.41 | 0.41 | 0.41 | 0.41 |
| Mitomycin C | 0.28 | -1.50 | -0.93 | 0.37 | 0.65 | 1.13 | 0.00 | -1.42 | -0.80 | 1.12 | 0.08 | 1.04 |
| Nintedanib | 0.64 | 0.64 | -0.13 | -1.88 | 0.78 | -0.06 | -0.12 | 0.93 | -0.02 | -1.80 | 0.93 | 0.09 |
| Palbociclib | -0.10 | -0.26 | 0.31 | -1.36 | NA | 1.40 | 0.45 | 0.45 | 0.45 | -1.79 | NA | 0.45 |
| Panobinostat | -0.11 | -0.92 | -0.78 | 1.86 | 0.14 | -0.19 | -0.25 | -0.88 | -0.41 | 1.97 | -0.16 | -0.28 |
| Paxalisib (GDC-0084) | -0.98 | 1.25 | -1.41 | 0.19 | 0.38 | 0.57 | 0.31 | 1.60 | -1.48 | -0.37 | -0.14 | 0.07 |
| Pinometostat | -1.47 | 1.05 | 0.24 | -0.47 | -0.47 | 1.11 | 0.41 | 0.41 | 0.41 | -2.04 | 0.41 | 0.41 |
| PRI-724 | -0.03 | 0.99 | -0.07 | -1.88 | 0.51 | 0.48 | 0.56 | 0.56 | -0.25 | -1.93 | 0.56 | 0.51 |
| Regorafenib | 0.50 | -0.82 | -1.00 | 1.08 | 1.08 | -0.84 | 0.00 | 0.00 | 0.00 | 0.00 | 0.00 | 0.00 |
| Ruxolitinib (INCB018424) | 0.40 | 0.47 | -1.56 | -0.91 | 0.61 | 1.00 | 0.00 | 0.00 | 0.00 | 0.00 | 0.00 | 0.00 |
| SN-38 | -0.04 | -1.14 | -1.18 | 1.30 | 0.67 | 0.39 | 0.29 | -1.14 | -1.18 | 1.34 | 0.66 | 0.02 |
| Sorafenib | 0.59 | -0.14 | 0.54 | -1.96 | 0.43 | 0.55 | 0.41 | 0.41 | 0.41 | -2.04 | 0.41 | 0.41 |
| Talazoparib | -0.52 | -0.98 | -1.13 | 1.21 | 0.87 | 0.54 | 0.63 | -1.59 | -0.94 | 0.63 | 0.63 | 0.63 |
| Temozolomide | 0.46 | 0.45 | 0.42 | -2.04 | 0.44 | 0.27 | 0.41 | 0.41 | 0.41 | -2.04 | 0.41 | 0.41 |
| Thiotepa | 1.02 | 0.37 | -0.73 | -1.48 | -0.21 | 1.02 | 0.41 | 0.41 | 0.41 | -2.04 | 0.41 | 0.41 |
| Topotecan hydrochloride | -0.41 | -0.52 | -0.62 | -0.97 | 1.09 | 1.43 | 0.57 | -0.81 | -0.86 | -0.96 | 1.36 | 0.70 |
| Trametinib | -1.85 | 0.42 | -0.32 | 0.73 | 0.18 | 0.85 | 0.00 | 0.00 | 0.00 | 0.00 | 0.00 | 0.00 |
| Vemurafenib | 0.56 | 0.40 | 0.30 | -2.03 | 0.43 | 0.35 | 0.41 | 0.41 | 0.41 | -2.04 | 0.41 | 0.41 |
| Venetoclax | -0.28 | -1.75 | 0.01 | 0.89 | 0.11 | 1.02 | 0.58 | -1.84 | 0.58 | 0.58 | -0.50 | 0.58 |
| Vincristine sulfate | -0.92 | -0.39 | -1.31 | 0.88 | 0.91 | 0.84 | -0.83 | -0.82 | -1.04 | 0.57 | 1.06 | 1.06 |
| Volasertib | -0.57 | -0.83 | -1.25 | 0.93 | 1.00 | 0.73 | -0.49 | -0.96 | -1.03 | 1.17 | 1.17 | 0.13 |
| Voxtalisib | 1.20 | 0.01 | -0.33 | -1.72 | 0.16 | 0.68 | 0.57 | 0.57 | -2.00 | 0.57 | 0.12 | 0.18 |

NA = Not available
