# Supplementary figures and images for: High-throughput 3D engineered paediatric tumour models for precision medicine
Source: Mol Syst Biol. 2025 Oct 1;21(12):1748–77. doi: 10.1038/s44320-025-00152-y (PMC12673126; doi:10.1038/s44320-025-00152-y)

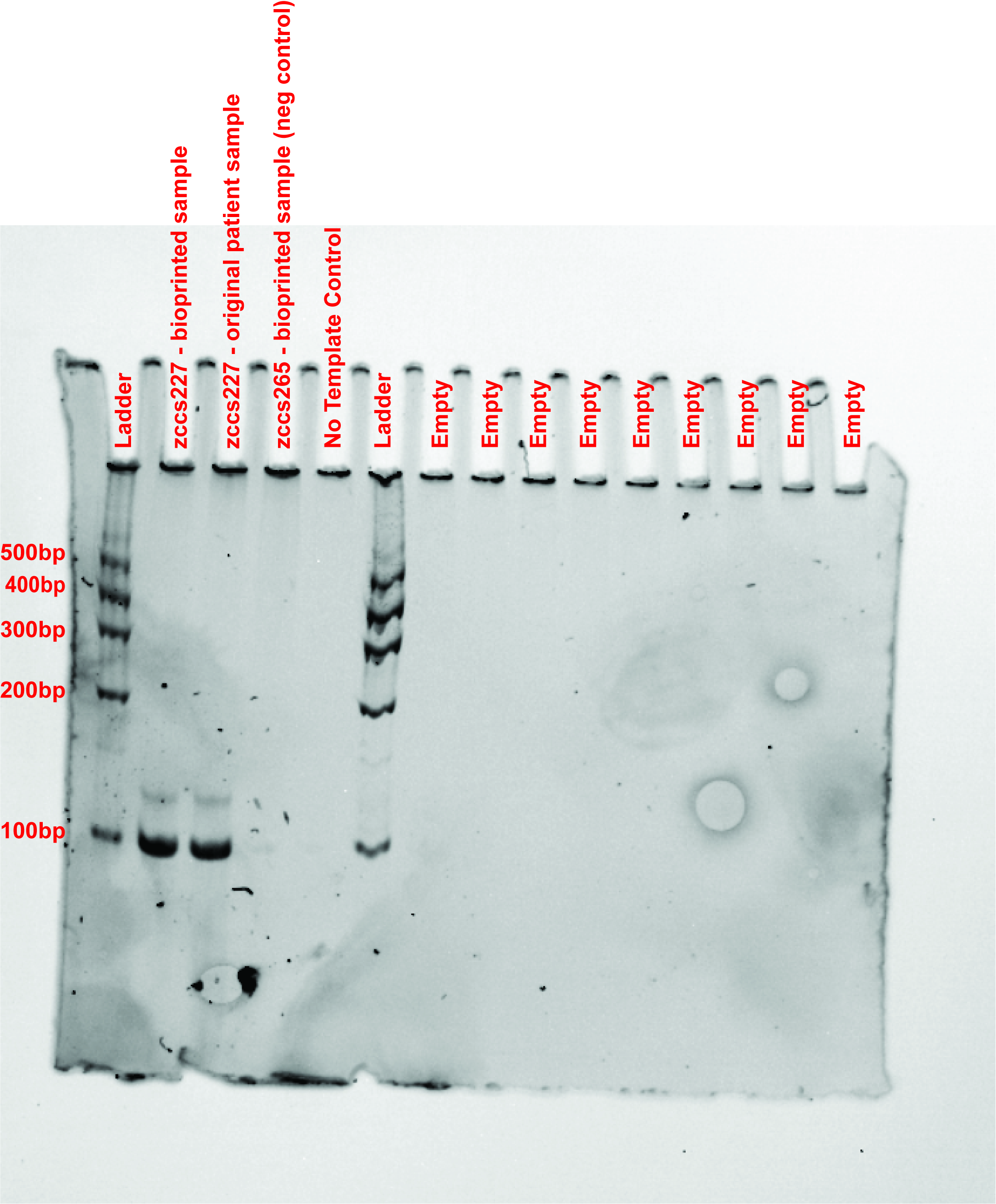

Supplement: Supplementary file 12 — Source data Fig. 3 [file 44320_2025_152_MOESM12_ESM.zip › Figure 3/3C/zccs227 PCR gel - Annotated.tif]

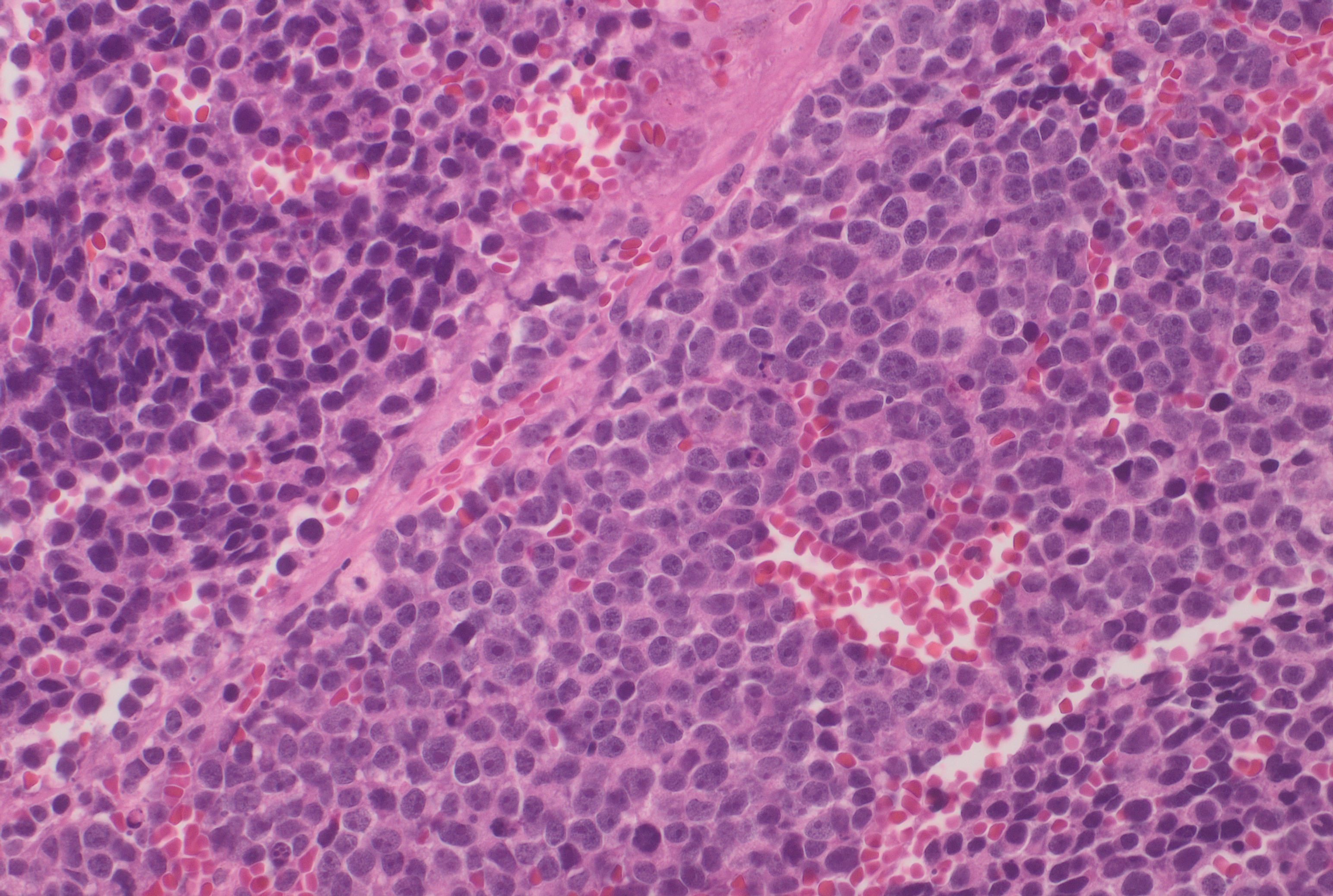

Supplement: Supplementary file 13 — Source data Fig. 4 [file 44320_2025_152_MOESM13_ESM.zip › Figure 4/4A/zccs373 Patient HE x40.tif]

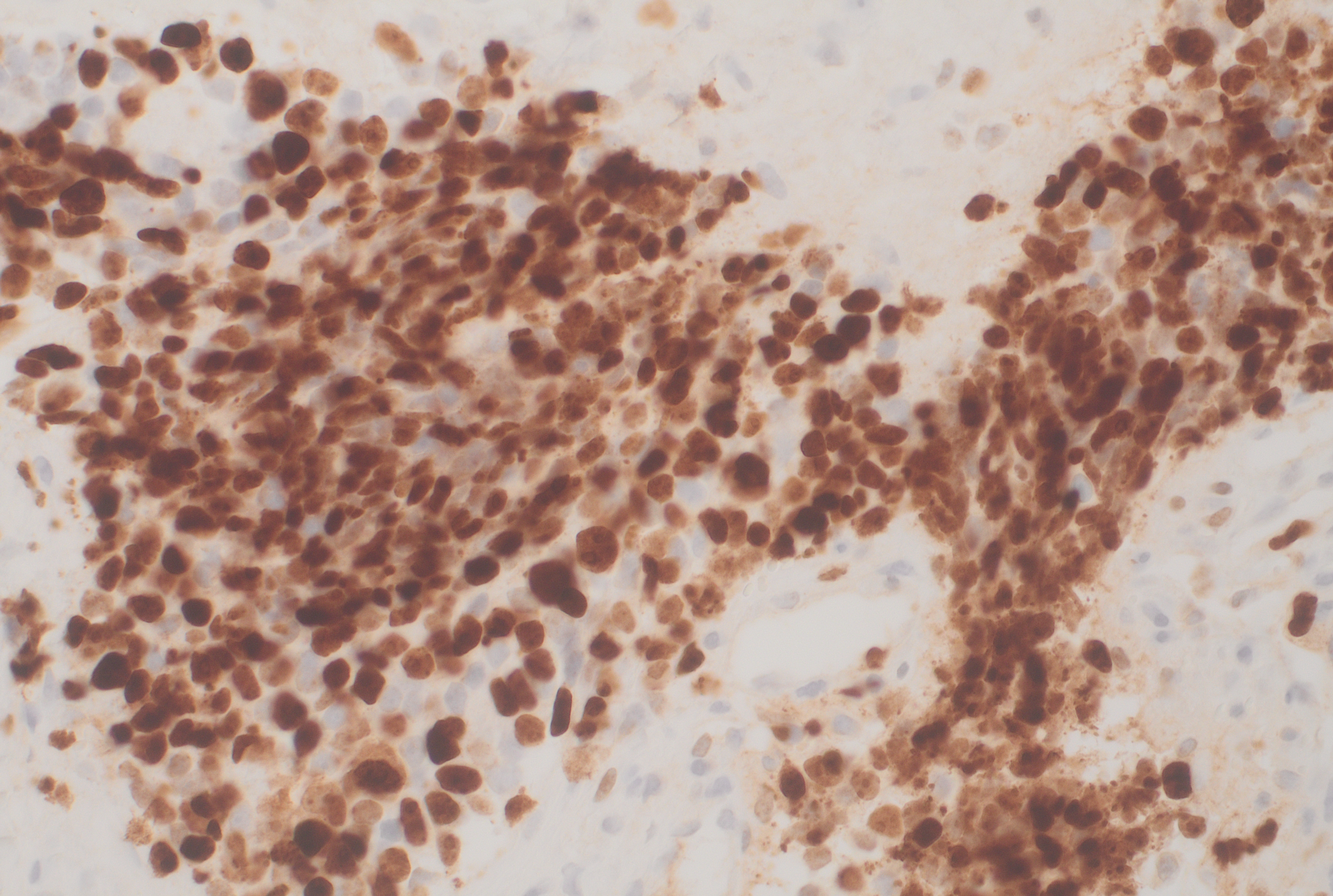

Supplement: Supplementary file 13 — Source data Fig. 4 [file 44320_2025_152_MOESM13_ESM.zip › Figure 4/4A/zccs373 Patient Ki67 x40.tif]

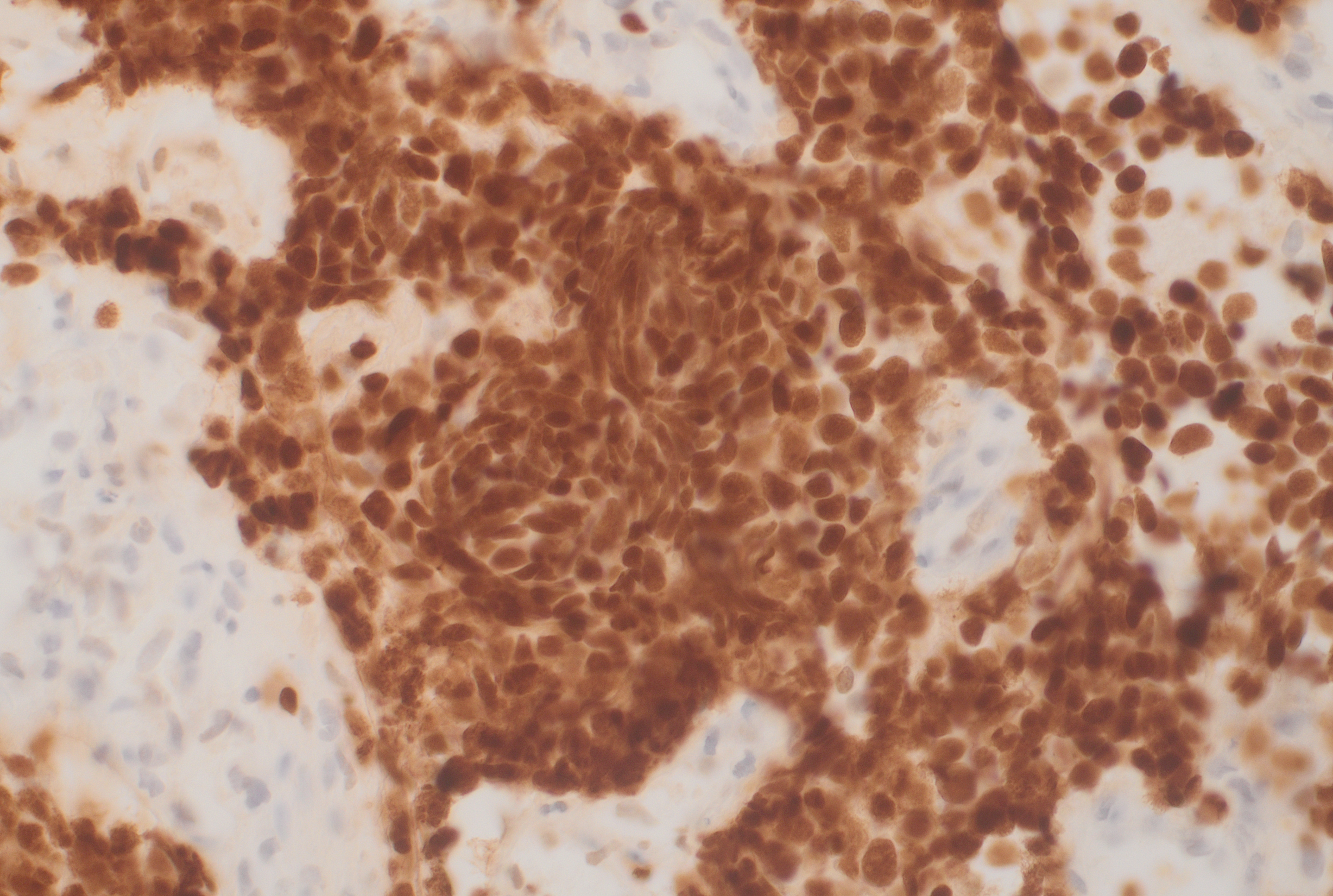

Supplement: Supplementary file 13 — Source data Fig. 4 [file 44320_2025_152_MOESM13_ESM.zip › Figure 4/4A/zccs373 Patient PHOX2B x40.tif]

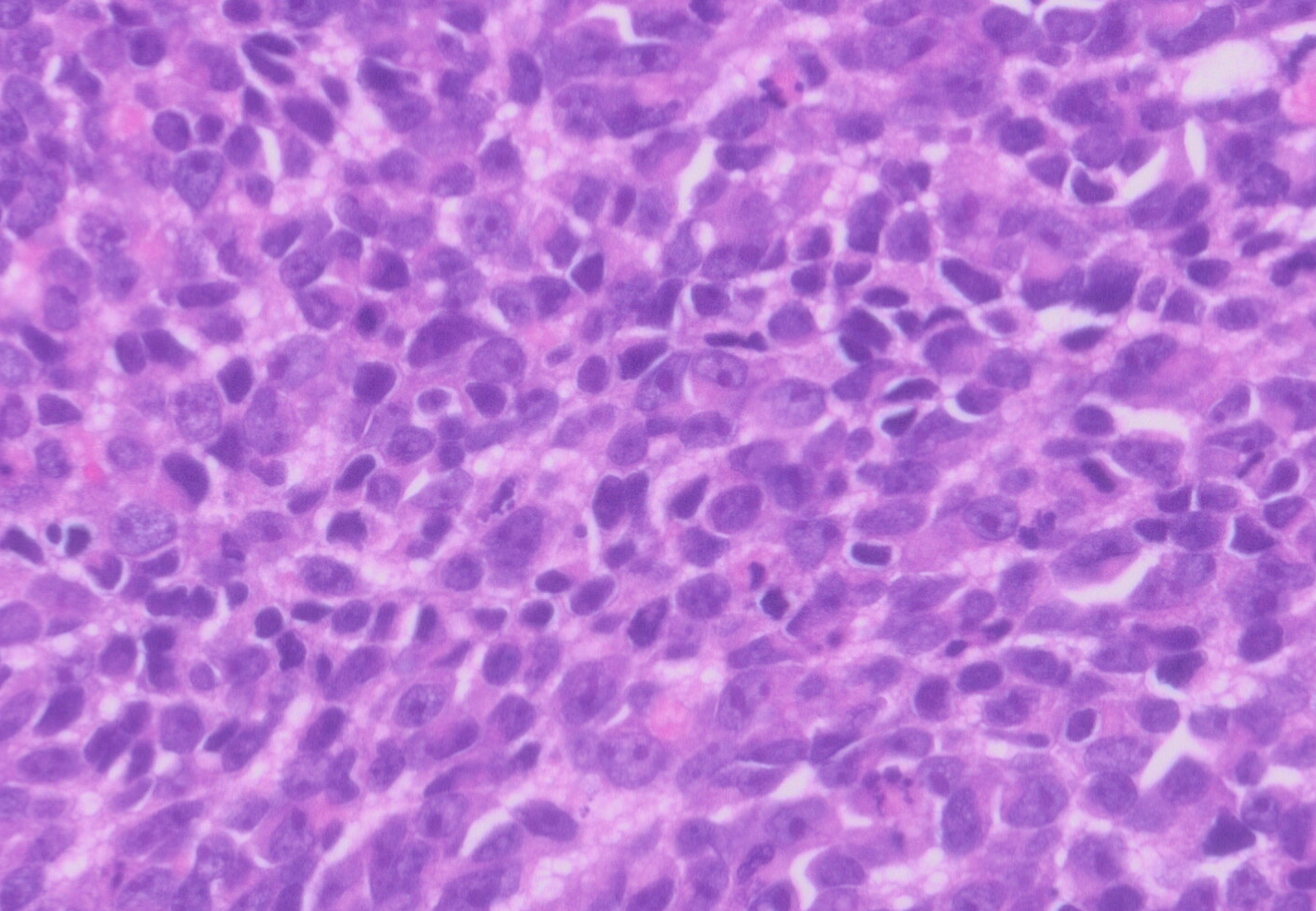

Supplement: Supplementary file 13 — Source data Fig. 4 [file 44320_2025_152_MOESM13_ESM.zip › Figure 4/4A/zccs373 PDX HE x40.tif]

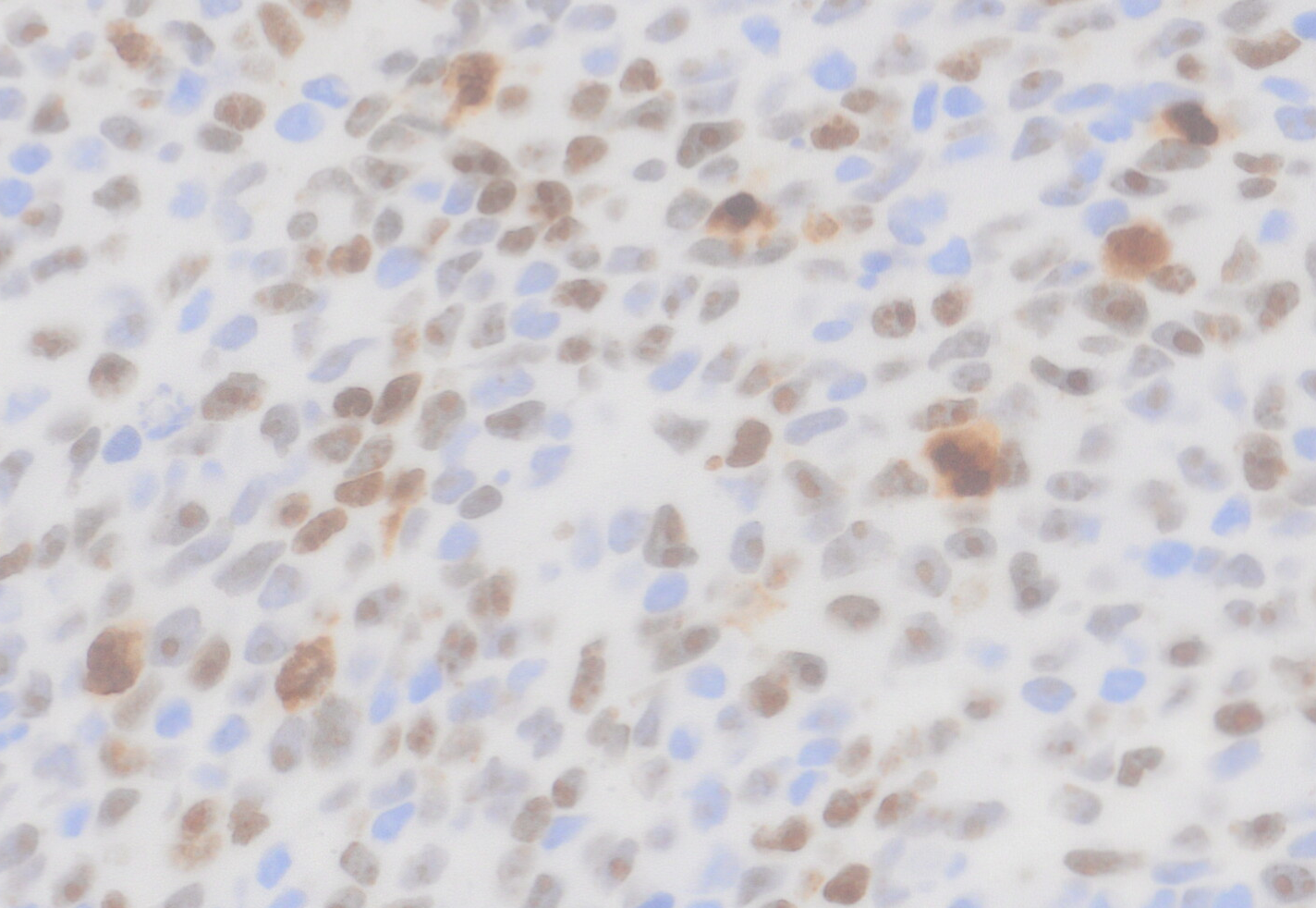

Supplement: Supplementary file 13 — Source data Fig. 4 [file 44320_2025_152_MOESM13_ESM.zip › Figure 4/4A/zccs373 PDX Ki67 x40.tif]

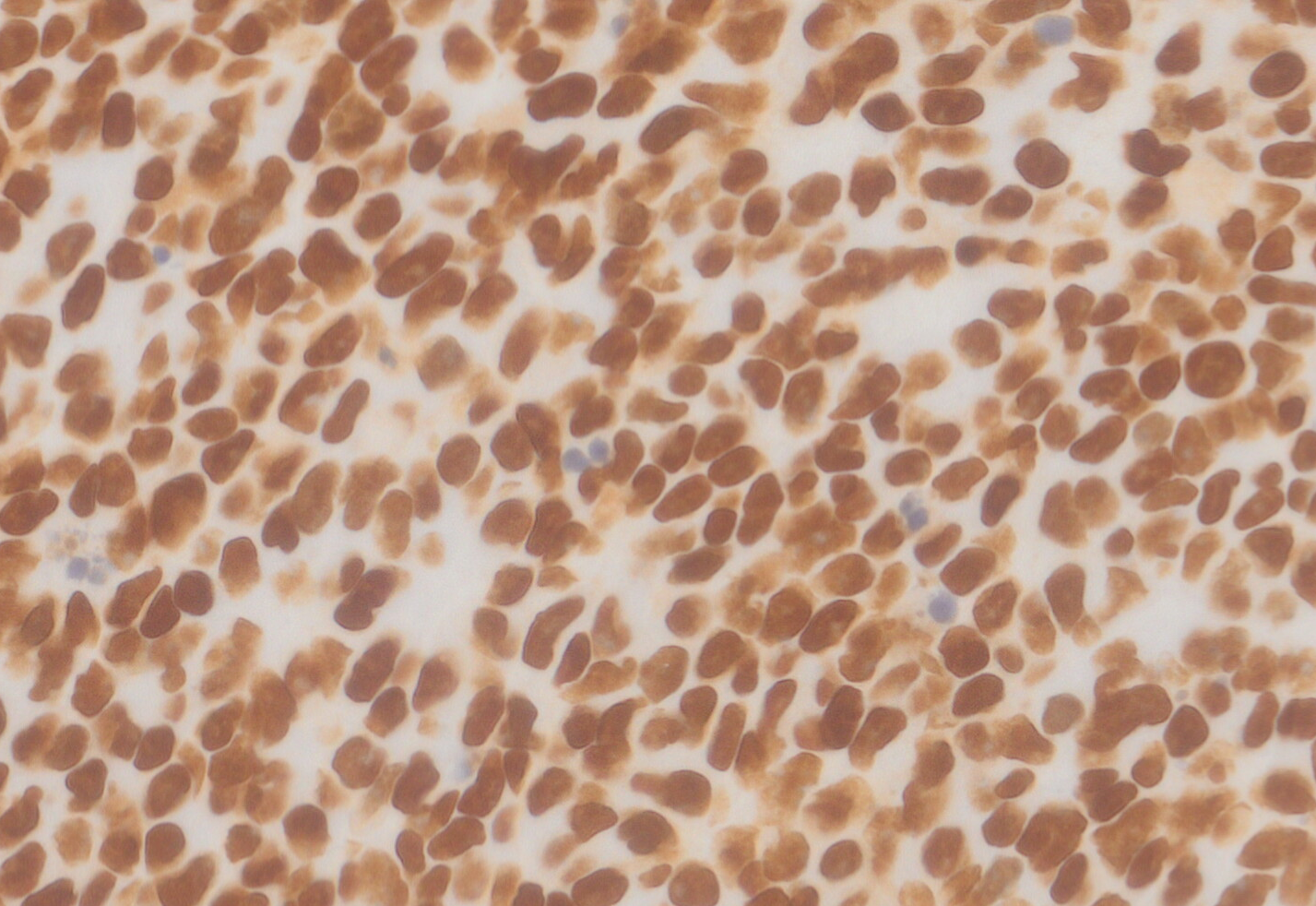

Supplement: Supplementary file 13 — Source data Fig. 4 [file 44320_2025_152_MOESM13_ESM.zip › Figure 4/4A/zccs373 PDX PHOX2B x40.tif]

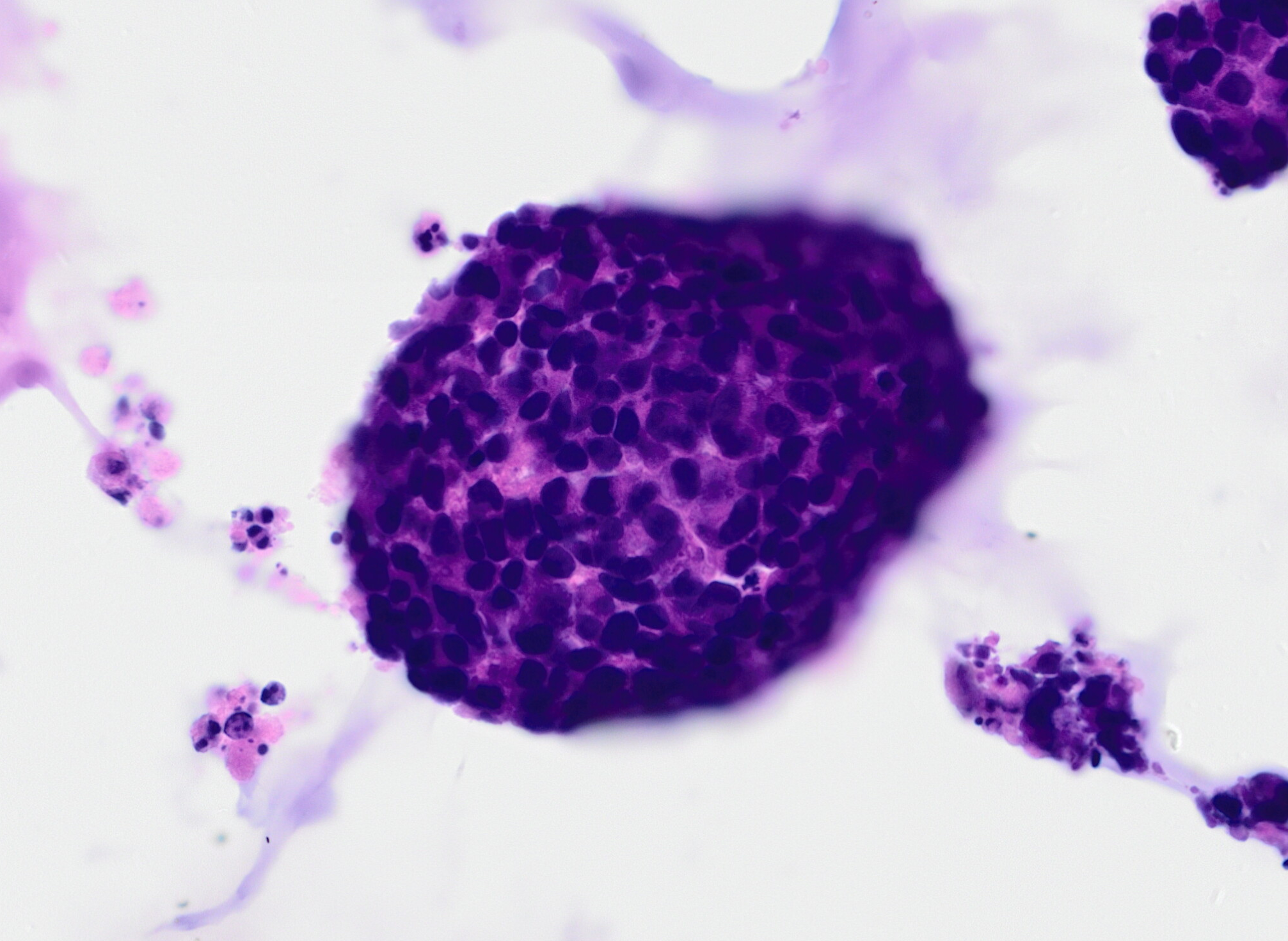

Supplement: Supplementary file 13 — Source data Fig. 4 [file 44320_2025_152_MOESM13_ESM.zip › Figure 4/4A/zccs373 Tumouroid HE x40.tif]

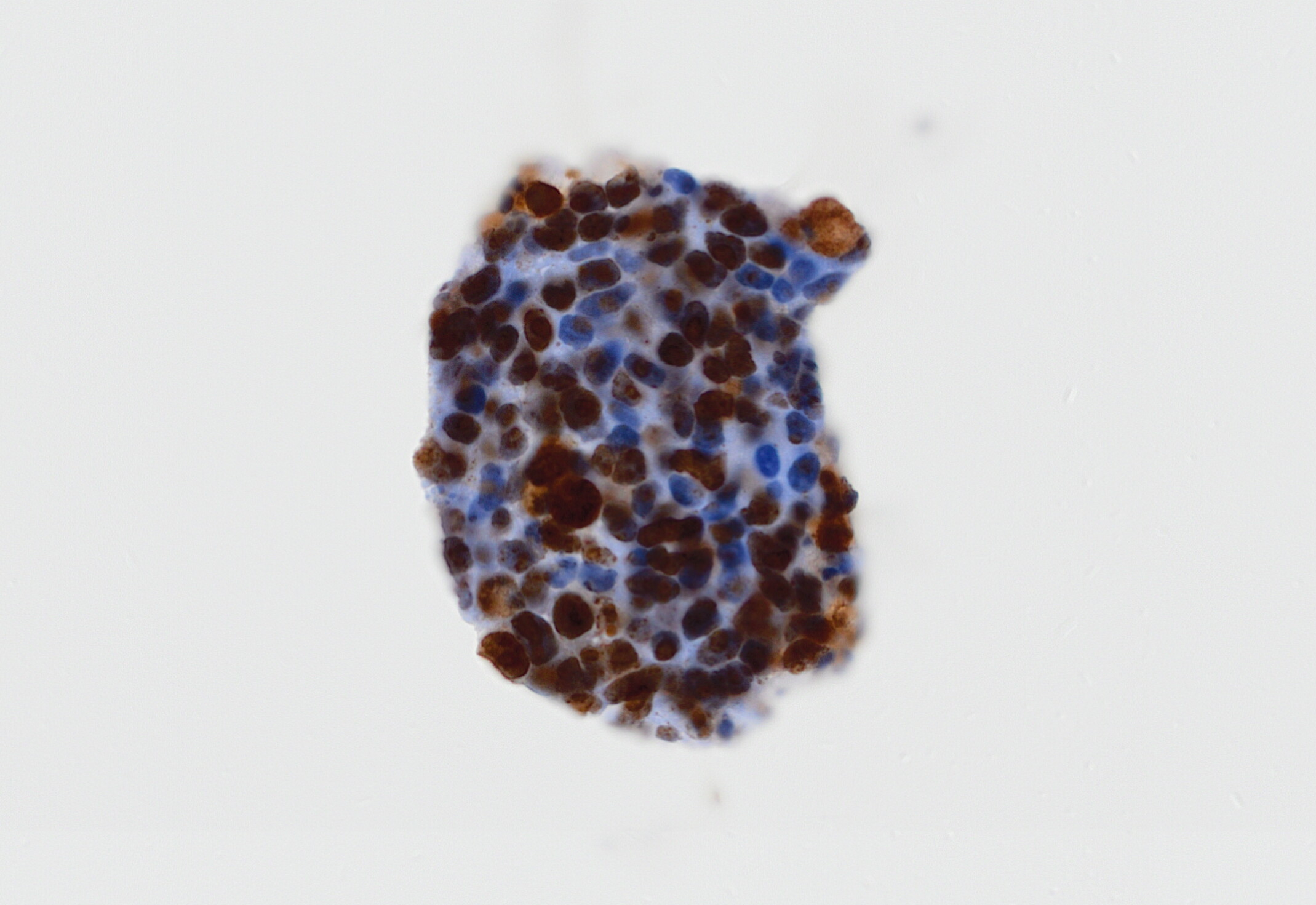

Supplement: Supplementary file 13 — Source data Fig. 4 [file 44320_2025_152_MOESM13_ESM.zip › Figure 4/4A/zccs373 Tumouroid Ki67 x40.tif]

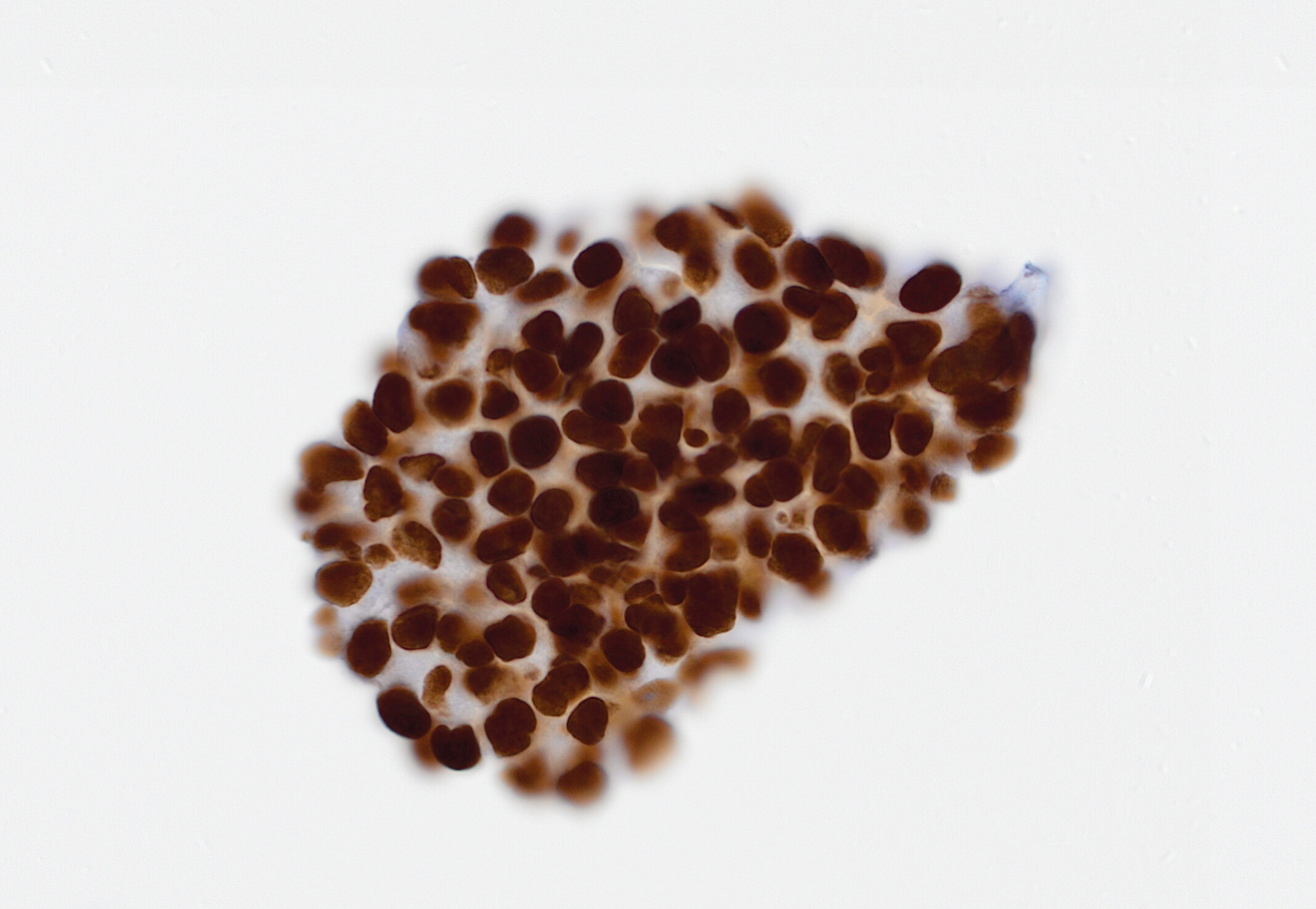

Supplement: Supplementary file 13 — Source data Fig. 4 [file 44320_2025_152_MOESM13_ESM.zip › Figure 4/4A/zccs373 Tumouroid PHOX2B x40.tif]

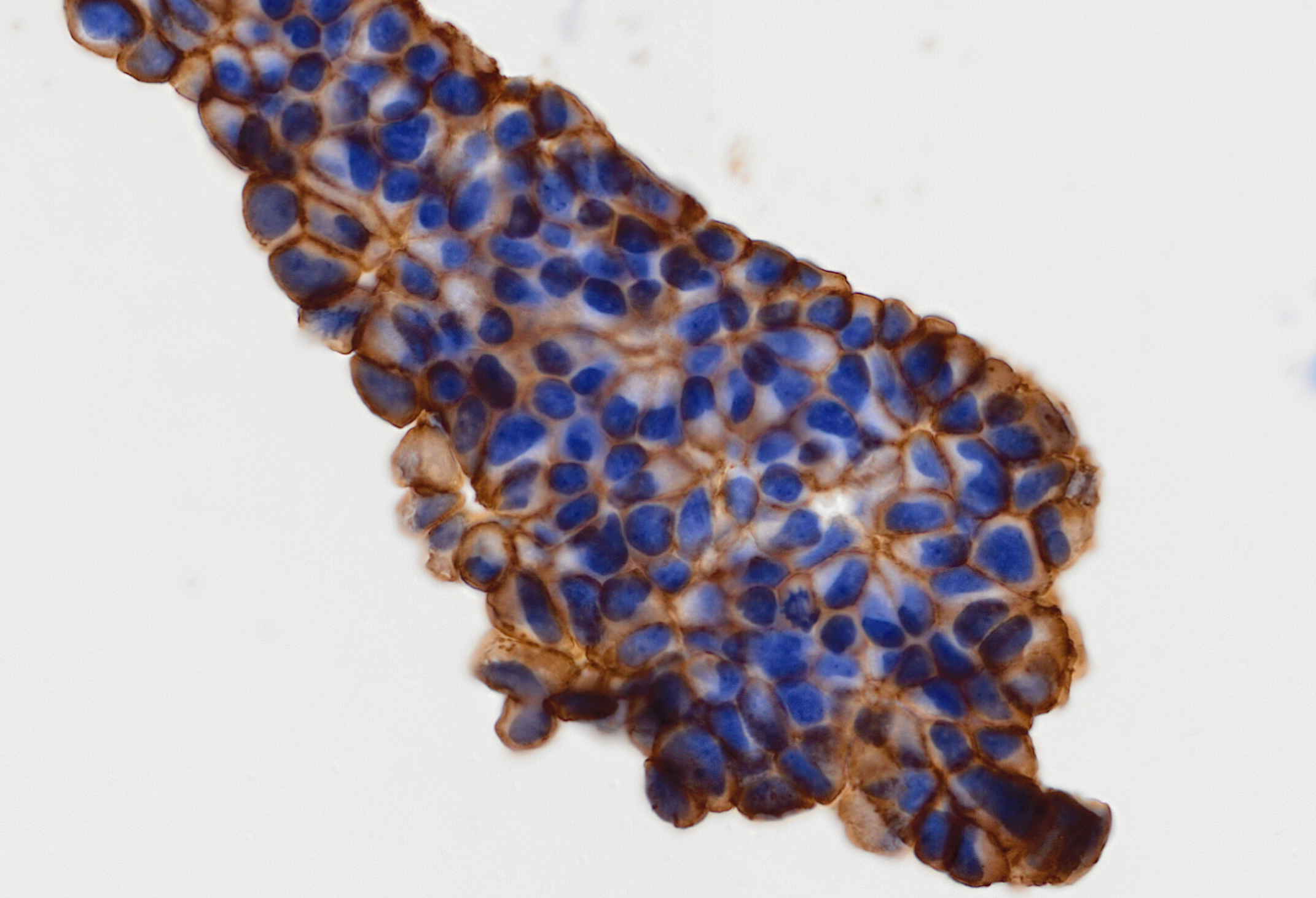

Supplement: Supplementary file 13 — Source data Fig. 4 [file 44320_2025_152_MOESM13_ESM.zip › Figure 4/4B/zccs207 Tumouroid CD99 x40.tif]

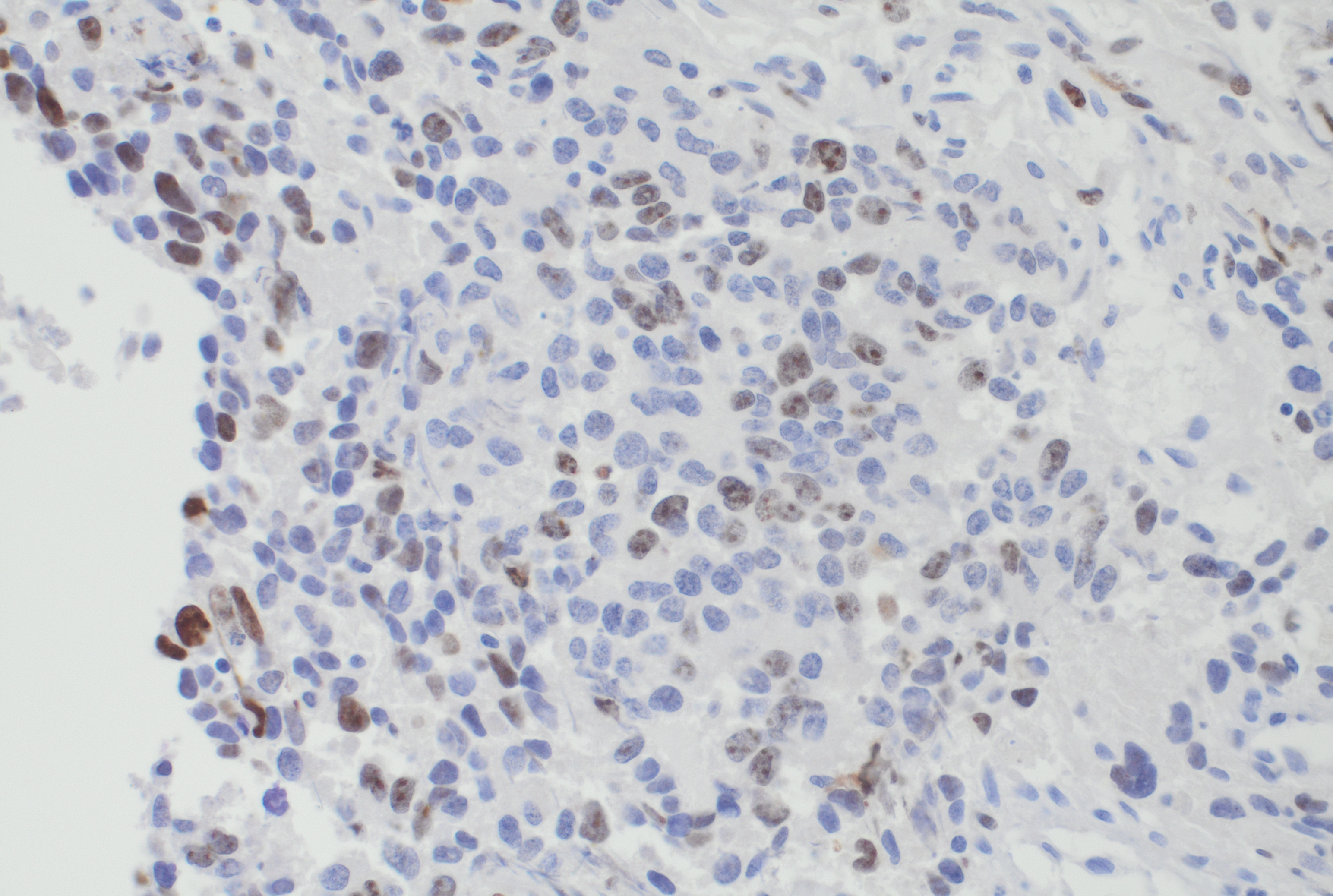

Supplement: Supplementary file 13 — Source data Fig. 4 [file 44320_2025_152_MOESM13_ESM.zip › Figure 4/4B/zccs207 Patient Ki67 x40.tif]

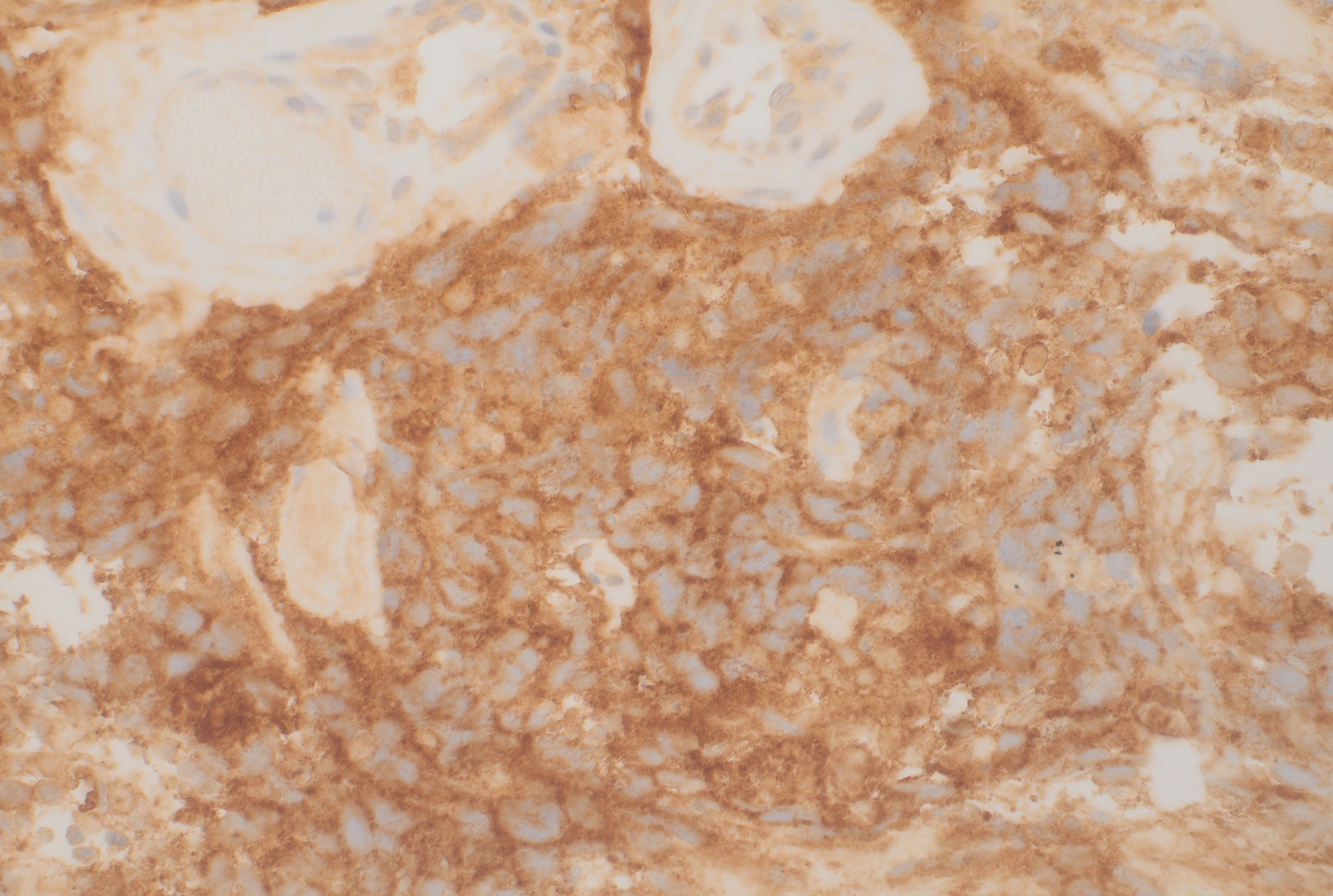

Supplement: Supplementary file 13 — Source data Fig. 4 [file 44320_2025_152_MOESM13_ESM.zip › Figure 4/4B/zccs207 Patient CD99 x40.tif]

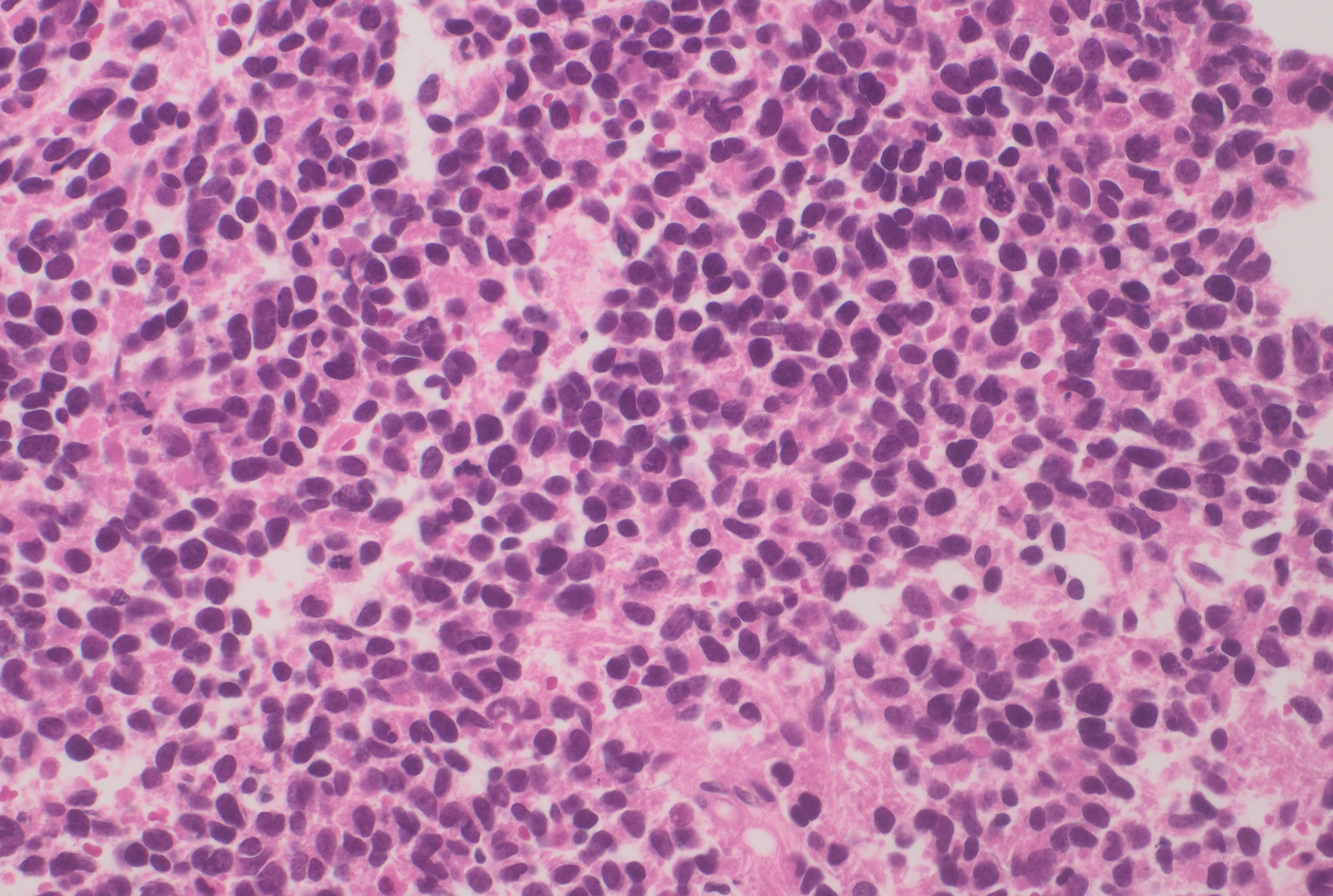

Supplement: Supplementary file 13 — Source data Fig. 4 [file 44320_2025_152_MOESM13_ESM.zip › Figure 4/4B/zccs207 Patient HE x40.tif]

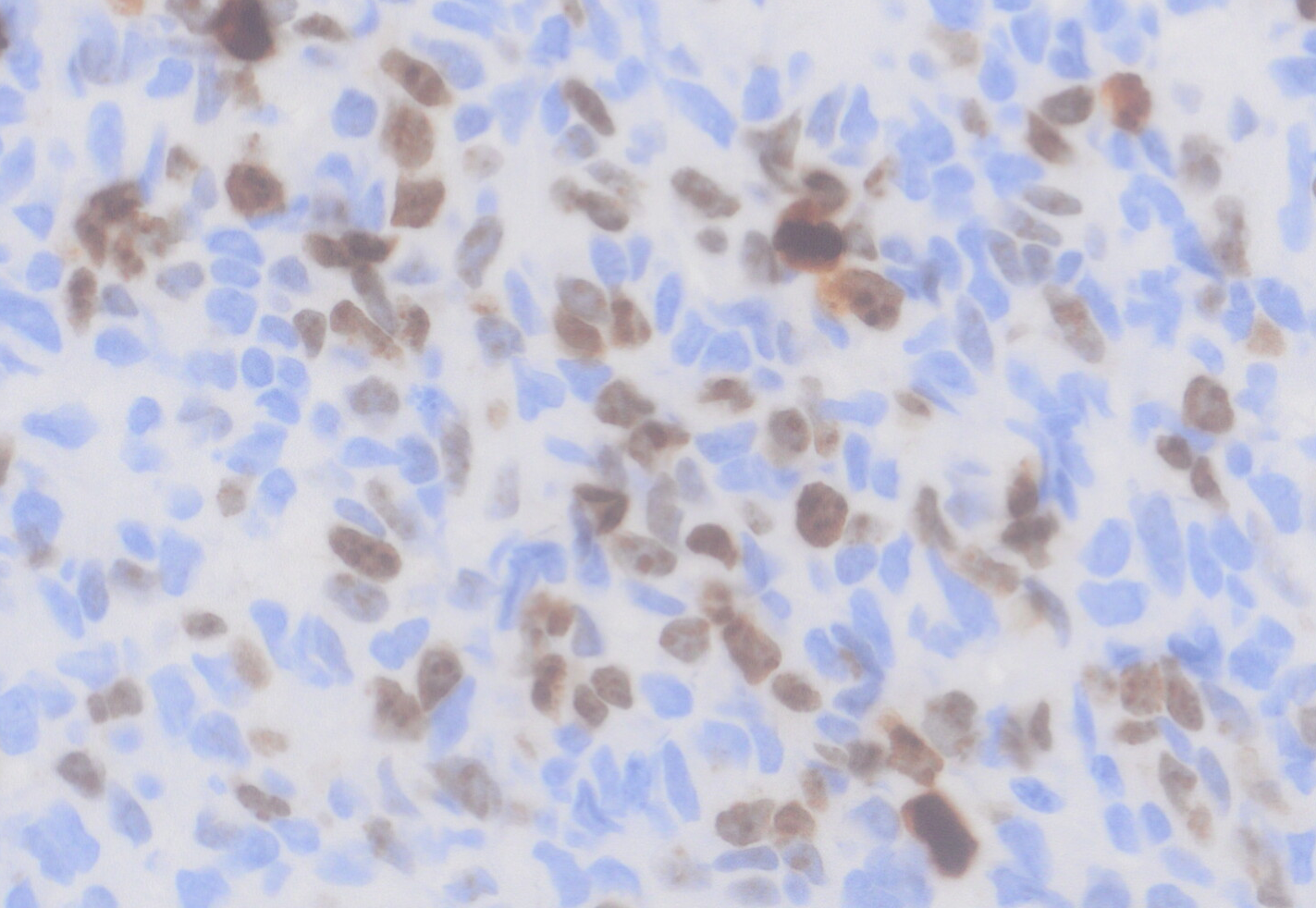

Supplement: Supplementary file 13 — Source data Fig. 4 [file 44320_2025_152_MOESM13_ESM.zip › Figure 4/4B/zccs207 PDX Ki67 x40.tif]

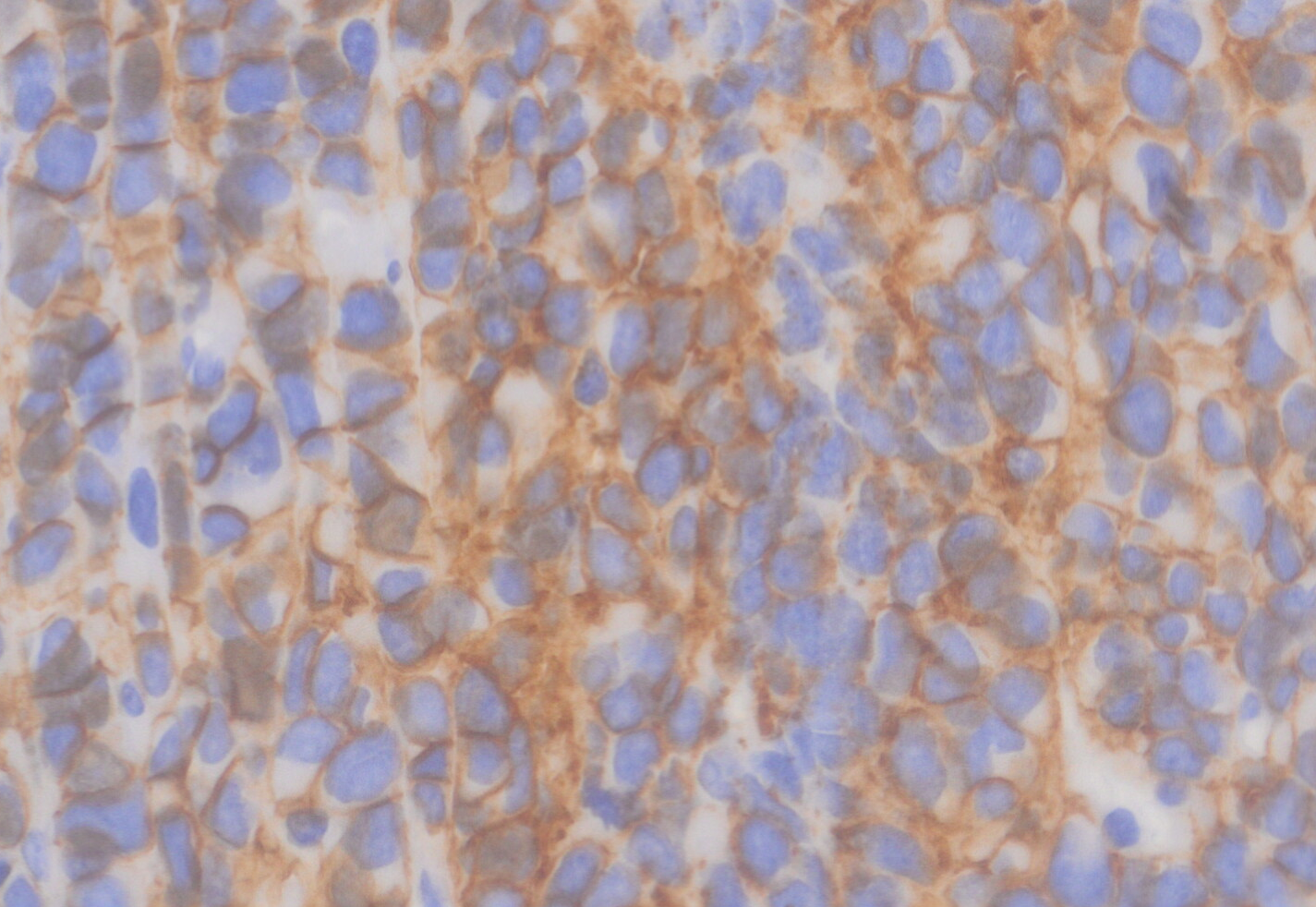

Supplement: Supplementary file 13 — Source data Fig. 4 [file 44320_2025_152_MOESM13_ESM.zip › Figure 4/4B/zccs207 PDX CD99 x40.tif]

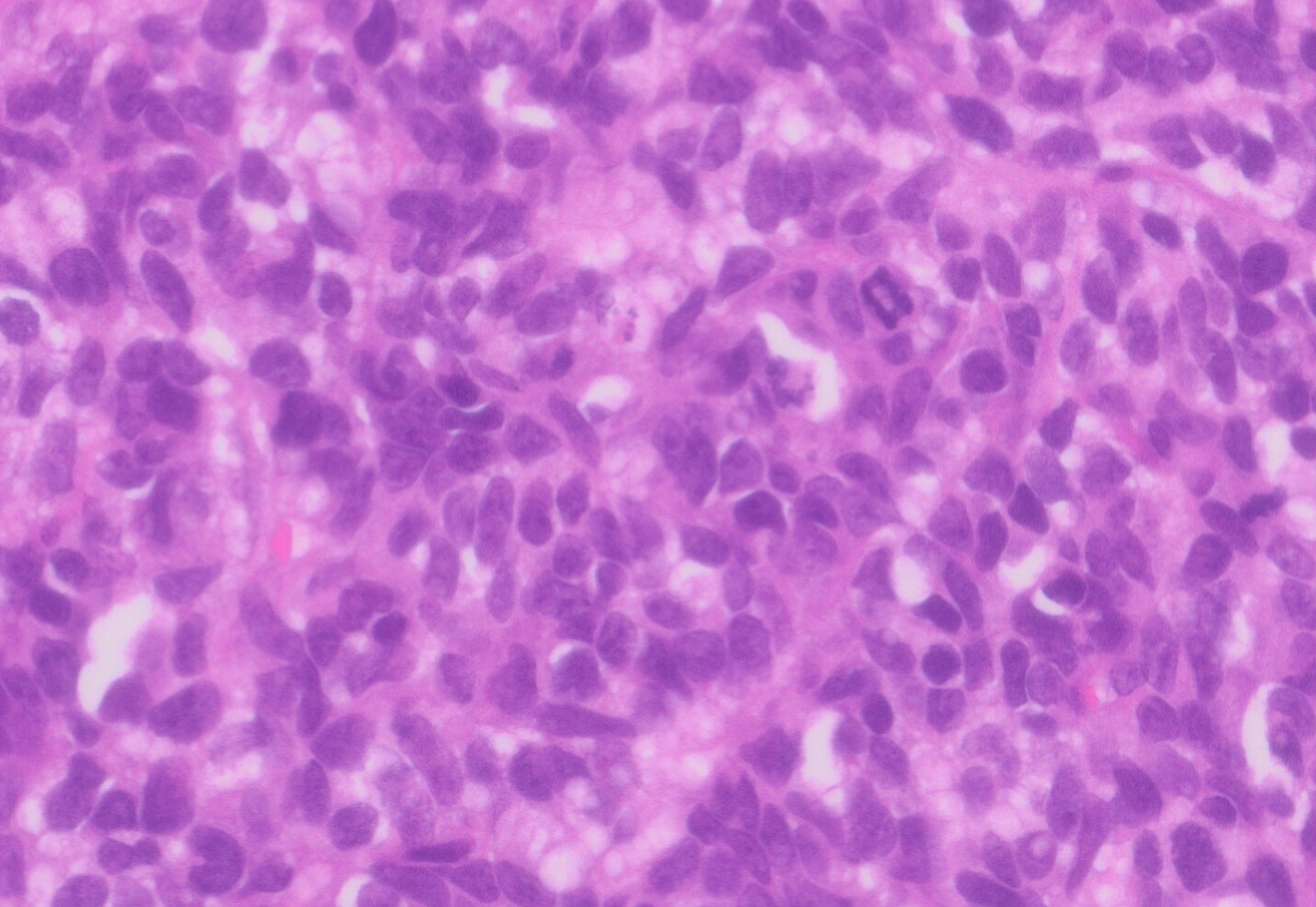

Supplement: Supplementary file 13 — Source data Fig. 4 [file 44320_2025_152_MOESM13_ESM.zip › Figure 4/4B/zccs207 PDX HE x40.tif]

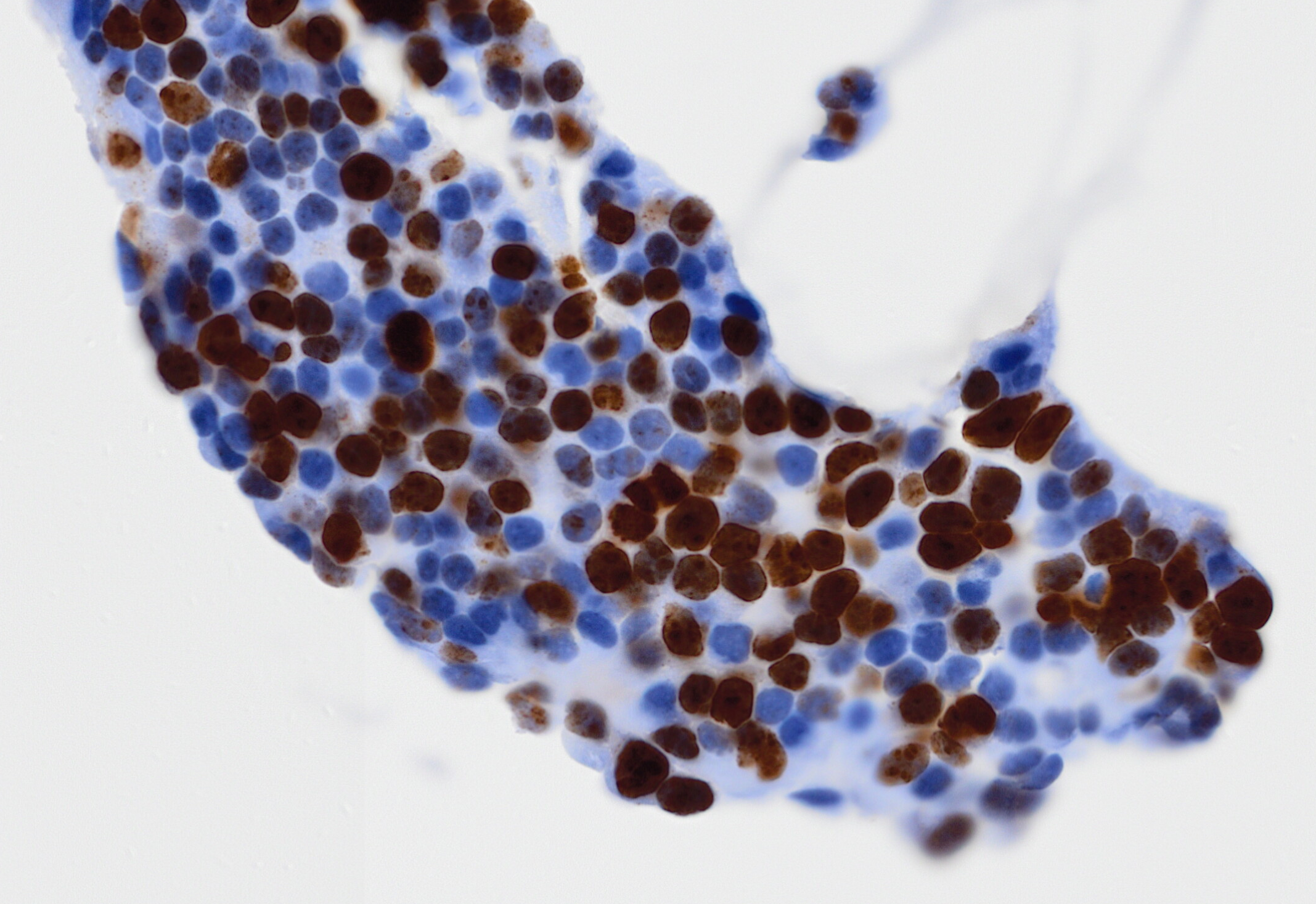

Supplement: Supplementary file 13 — Source data Fig. 4 [file 44320_2025_152_MOESM13_ESM.zip › Figure 4/4B/zccs207 Tumouroid Ki67 x40.tif]

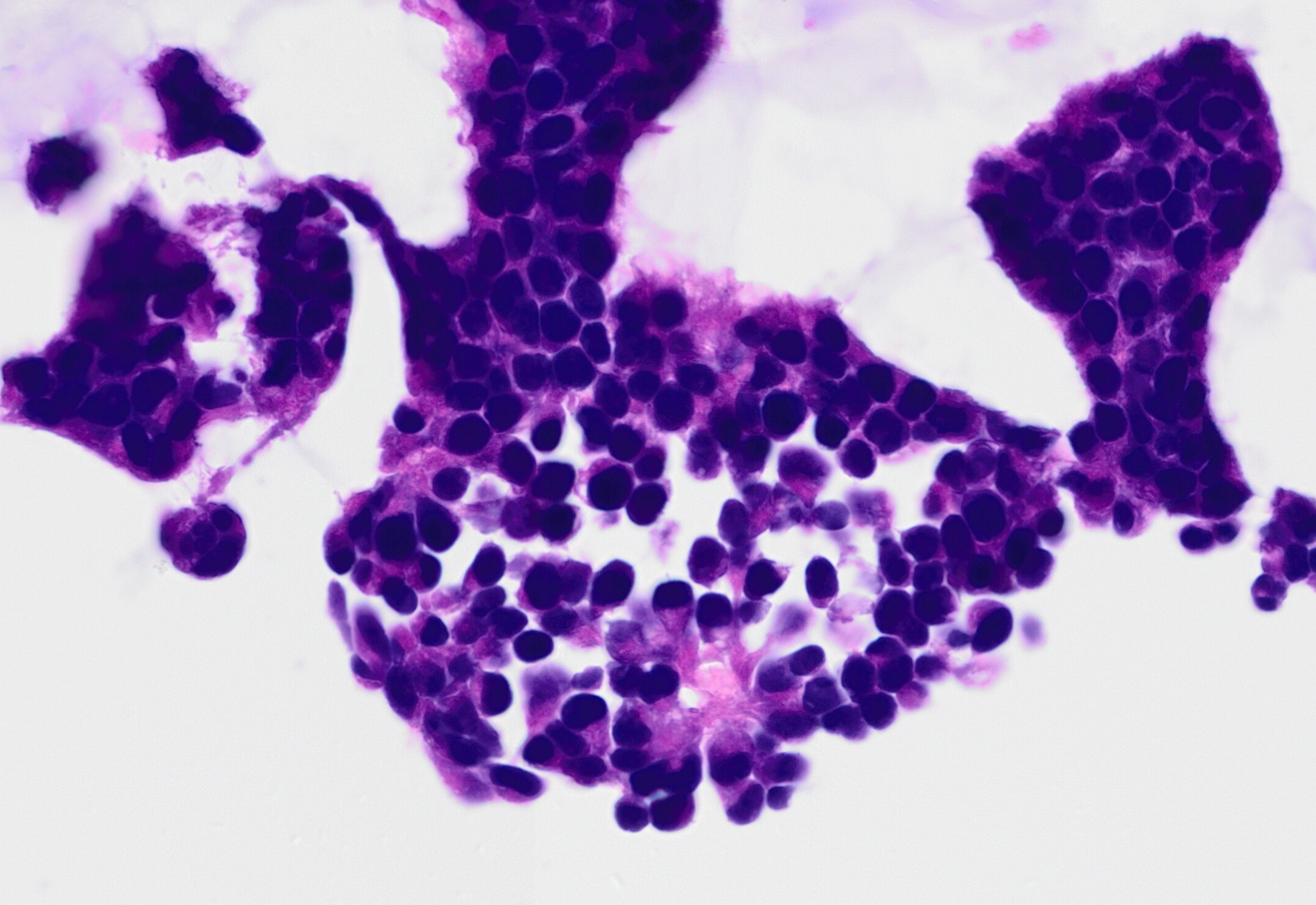

Supplement: Supplementary file 13 — Source data Fig. 4 [file 44320_2025_152_MOESM13_ESM.zip › Figure 4/4B/zccs207 Tumouroid HE x40.tif]

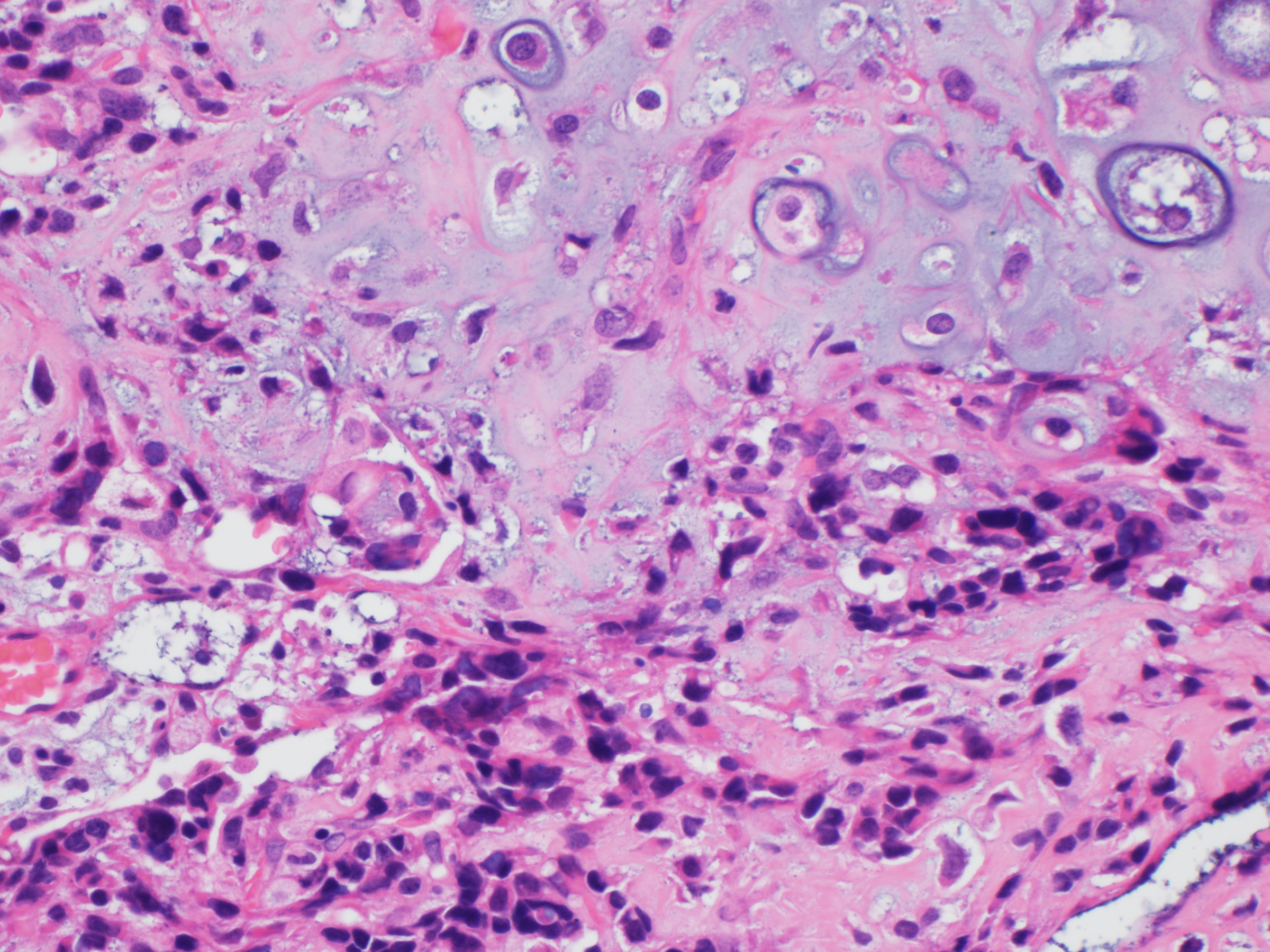

Supplement: Supplementary file 13 — Source data Fig. 4 [file 44320_2025_152_MOESM13_ESM.zip › Figure 4/4C/zccs225 Patient HE x40.tif]

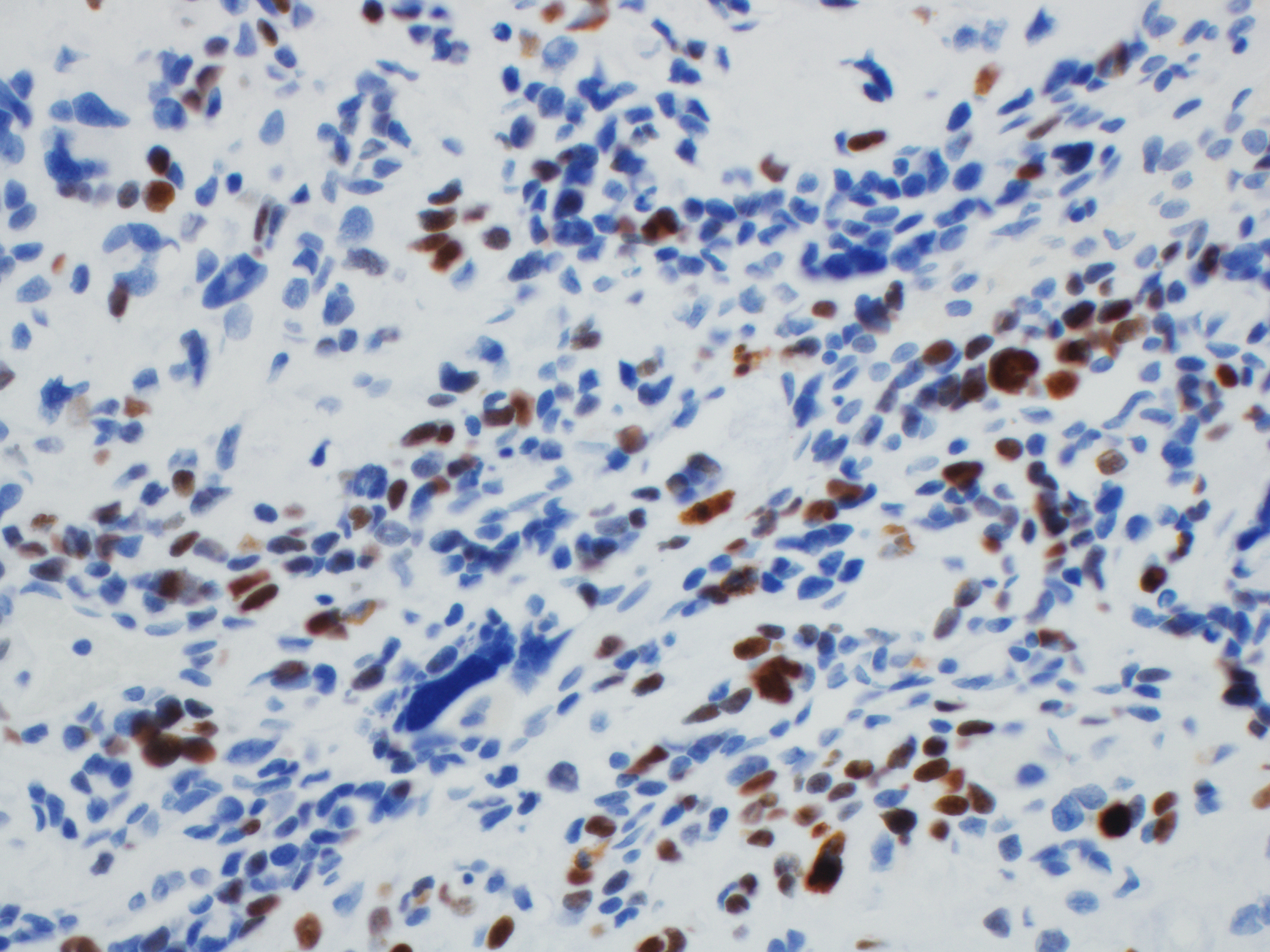

Supplement: Supplementary file 13 — Source data Fig. 4 [file 44320_2025_152_MOESM13_ESM.zip › Figure 4/4C/zccs225 Patient Ki67 x40.tif]

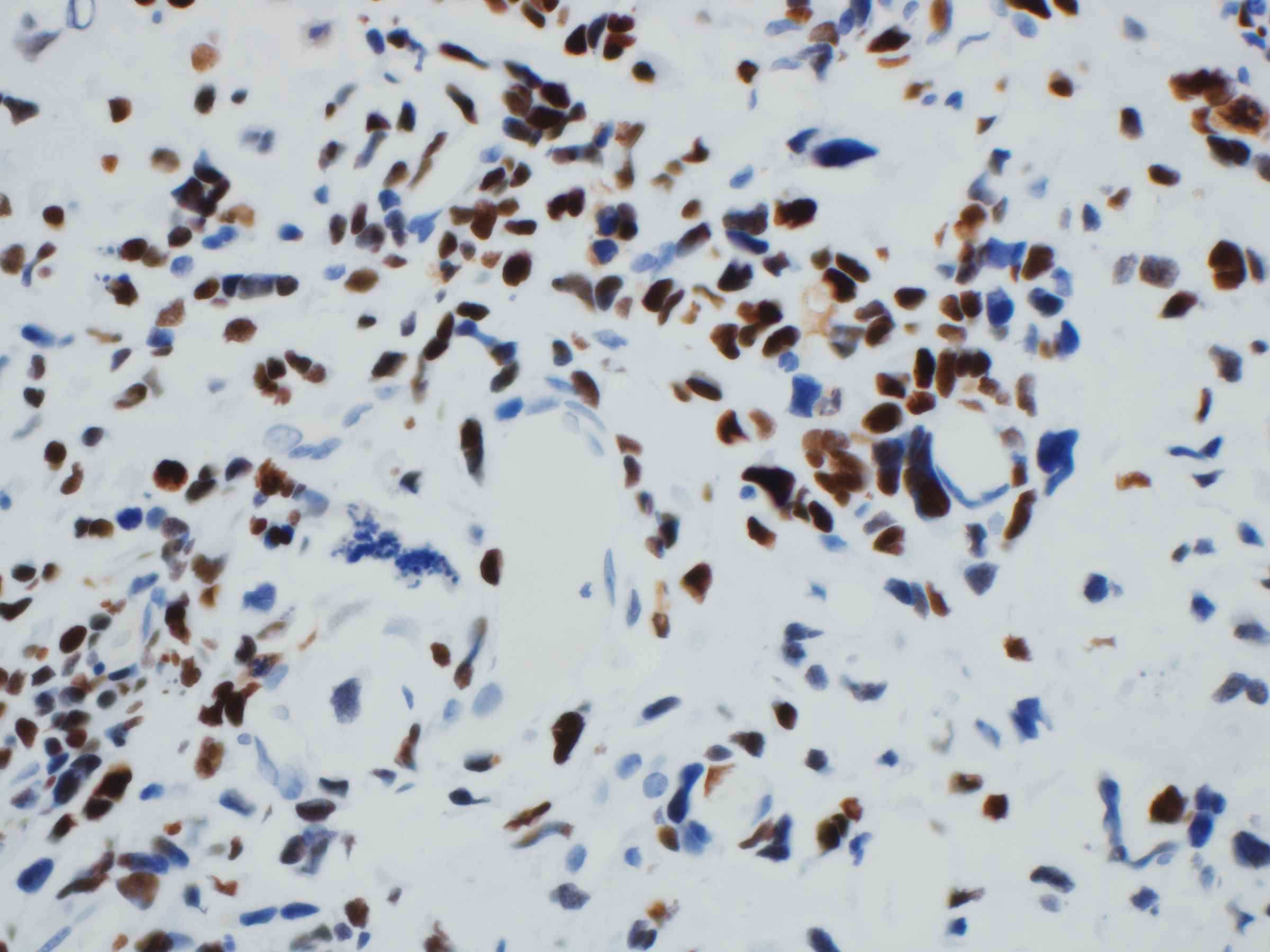

Supplement: Supplementary file 13 — Source data Fig. 4 [file 44320_2025_152_MOESM13_ESM.zip › Figure 4/4C/zccs225 Patient SATB2 x40.tif]

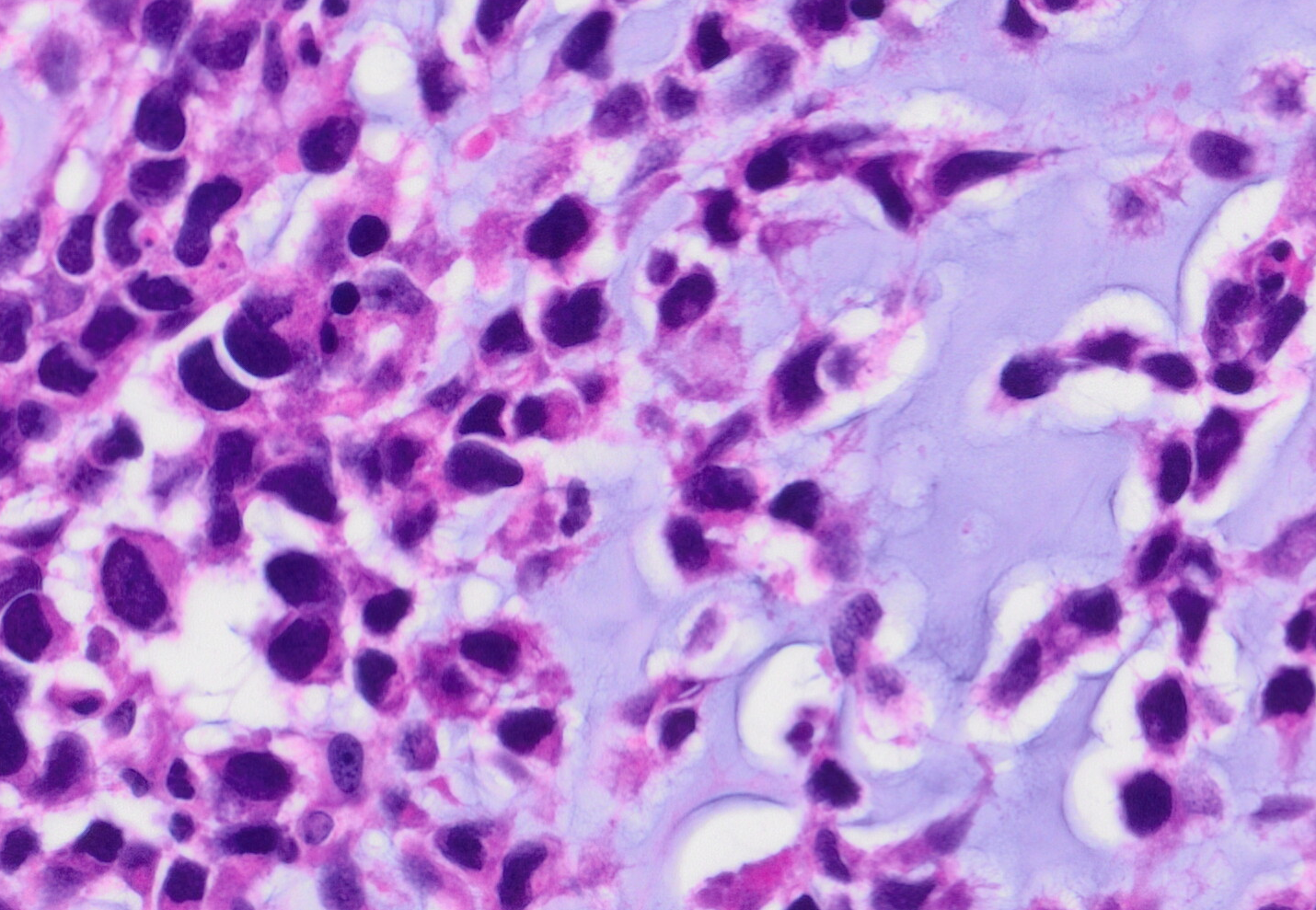

Supplement: Supplementary file 13 — Source data Fig. 4 [file 44320_2025_152_MOESM13_ESM.zip › Figure 4/4C/zccs225 PDX HE x40.tif]

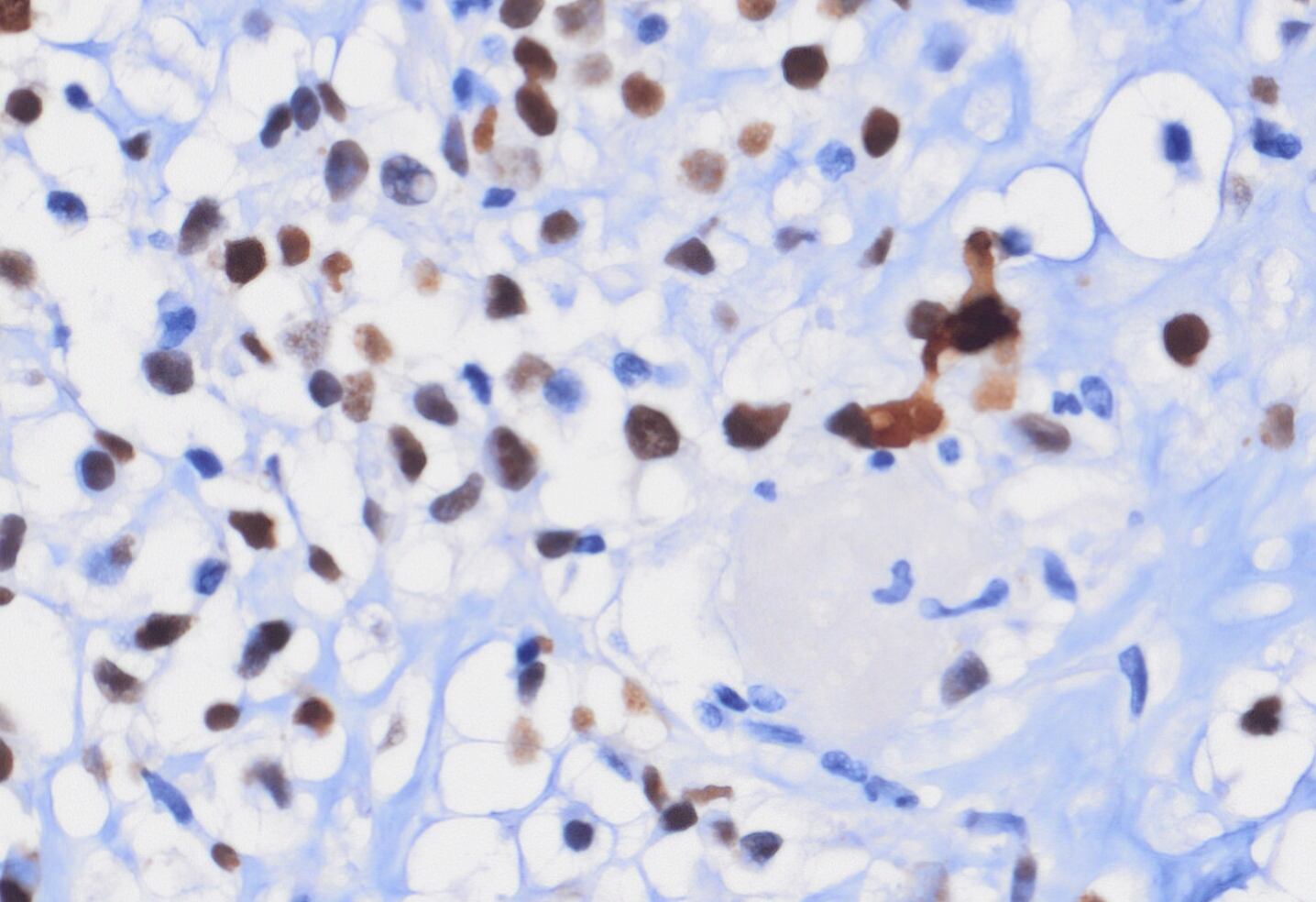

Supplement: Supplementary file 13 — Source data Fig. 4 [file 44320_2025_152_MOESM13_ESM.zip › Figure 4/4C/zccs225 PDX Ki67 x40.tif]

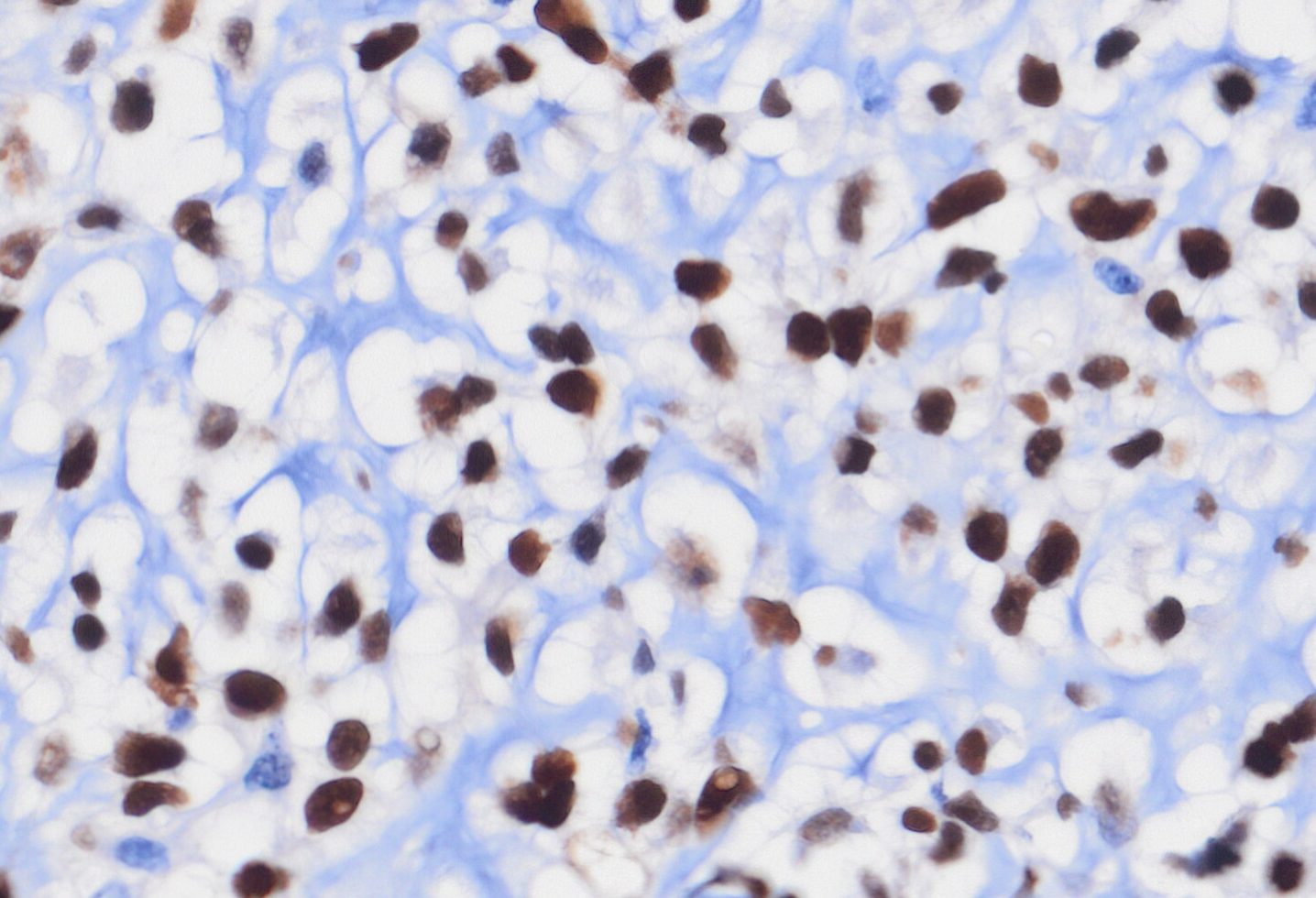

Supplement: Supplementary file 13 — Source data Fig. 4 [file 44320_2025_152_MOESM13_ESM.zip › Figure 4/4C/zccs225 PDX SATB2 x40.tif]

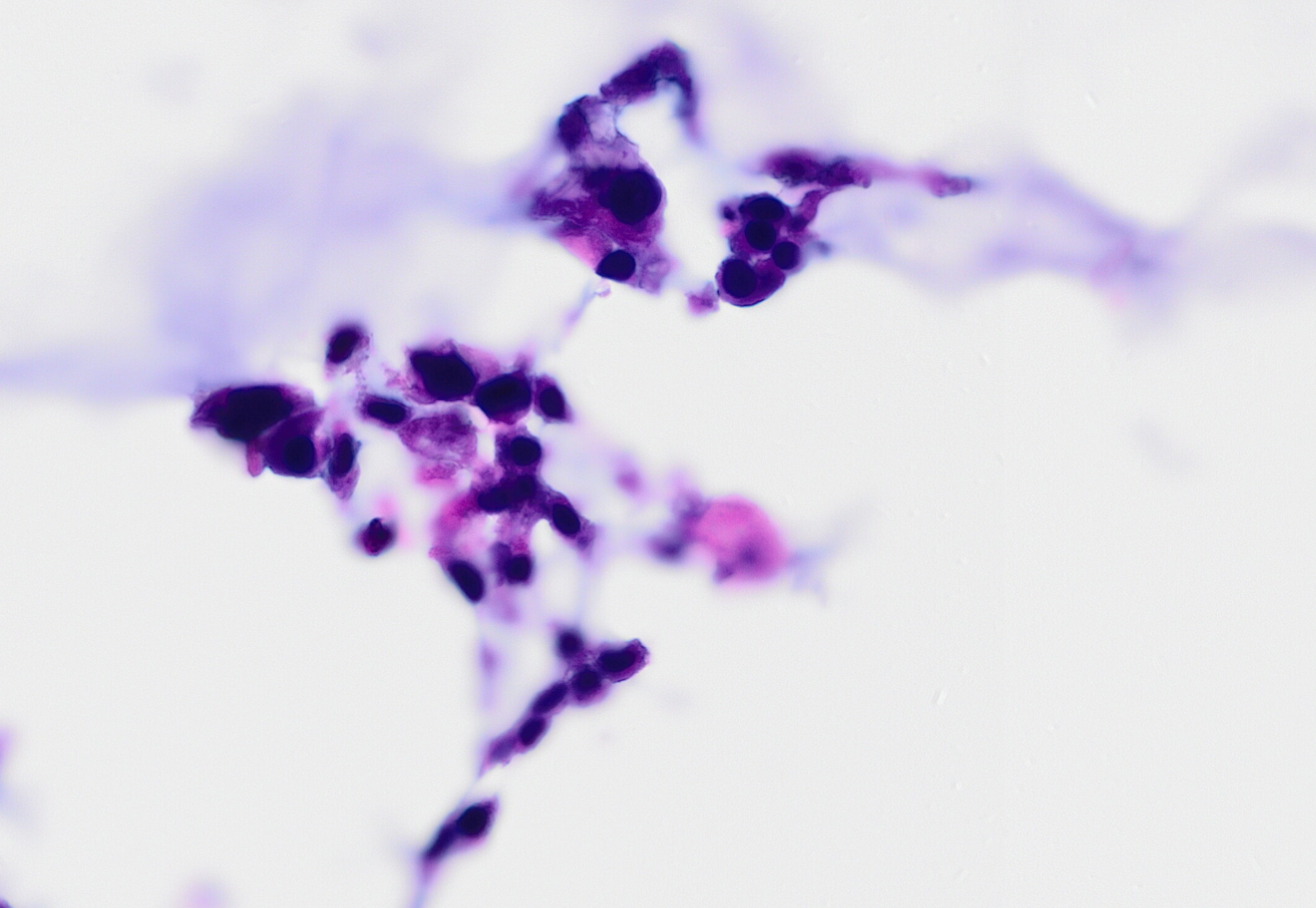

Supplement: Supplementary file 13 — Source data Fig. 4 [file 44320_2025_152_MOESM13_ESM.zip › Figure 4/4C/zccs225 Tumouroid HE x40.tif]

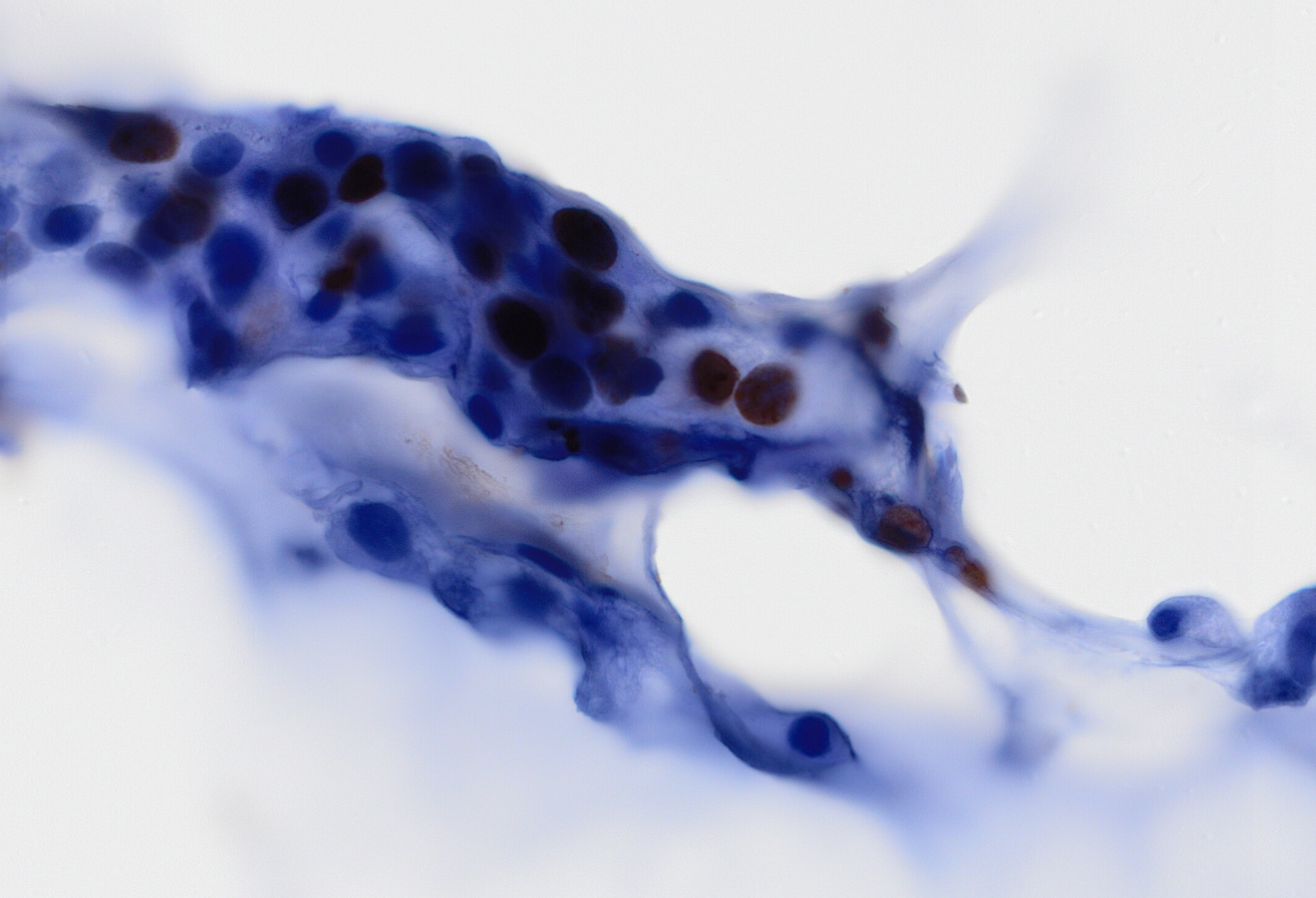

Supplement: Supplementary file 13 — Source data Fig. 4 [file 44320_2025_152_MOESM13_ESM.zip › Figure 4/4C/zccs225 Tumouroid Ki67 x40.tif]

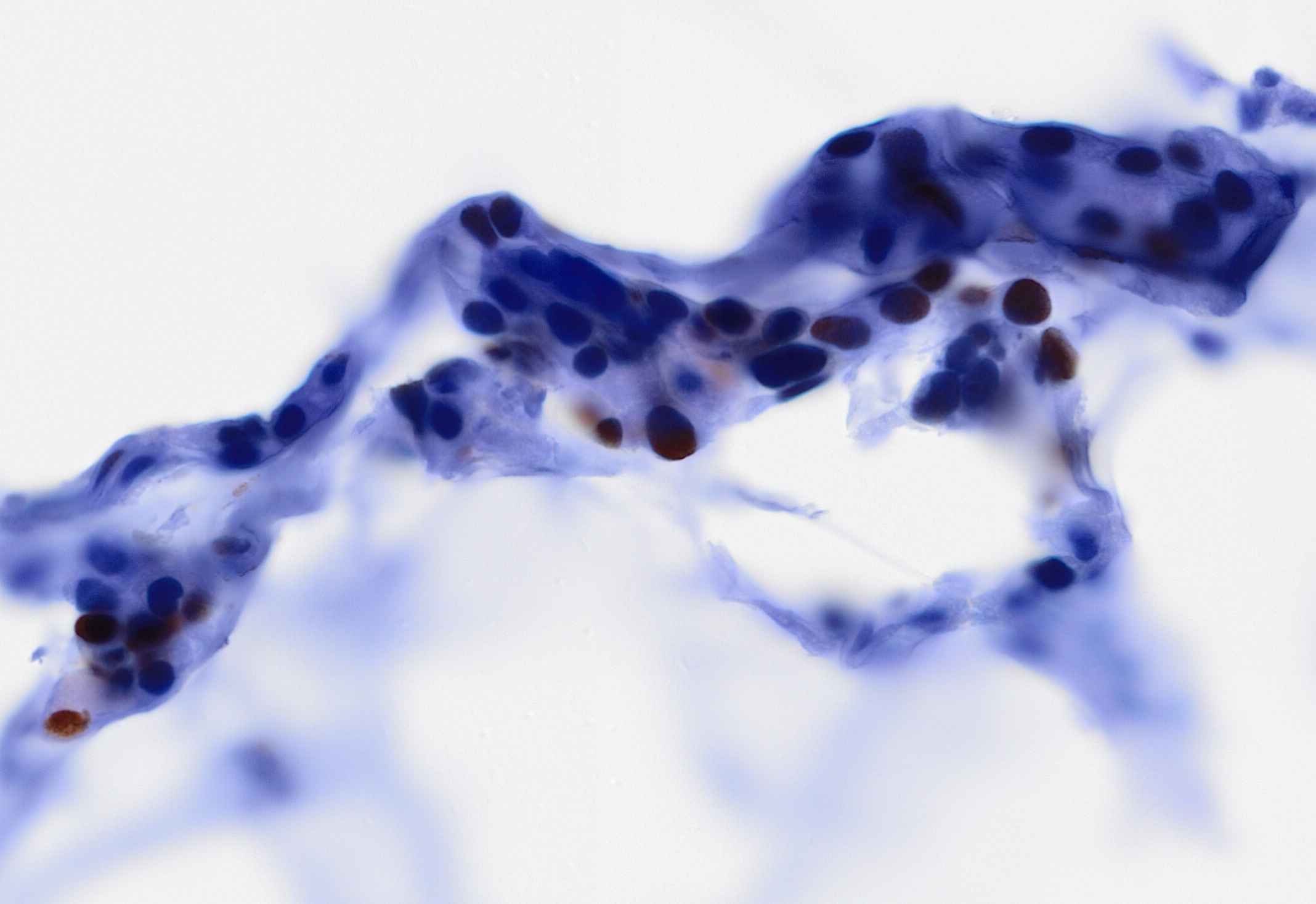

Supplement: Supplementary file 13 — Source data Fig. 4 [file 44320_2025_152_MOESM13_ESM.zip › Figure 4/4C/zccs225 Tumouroid SATB2 x40.tif]

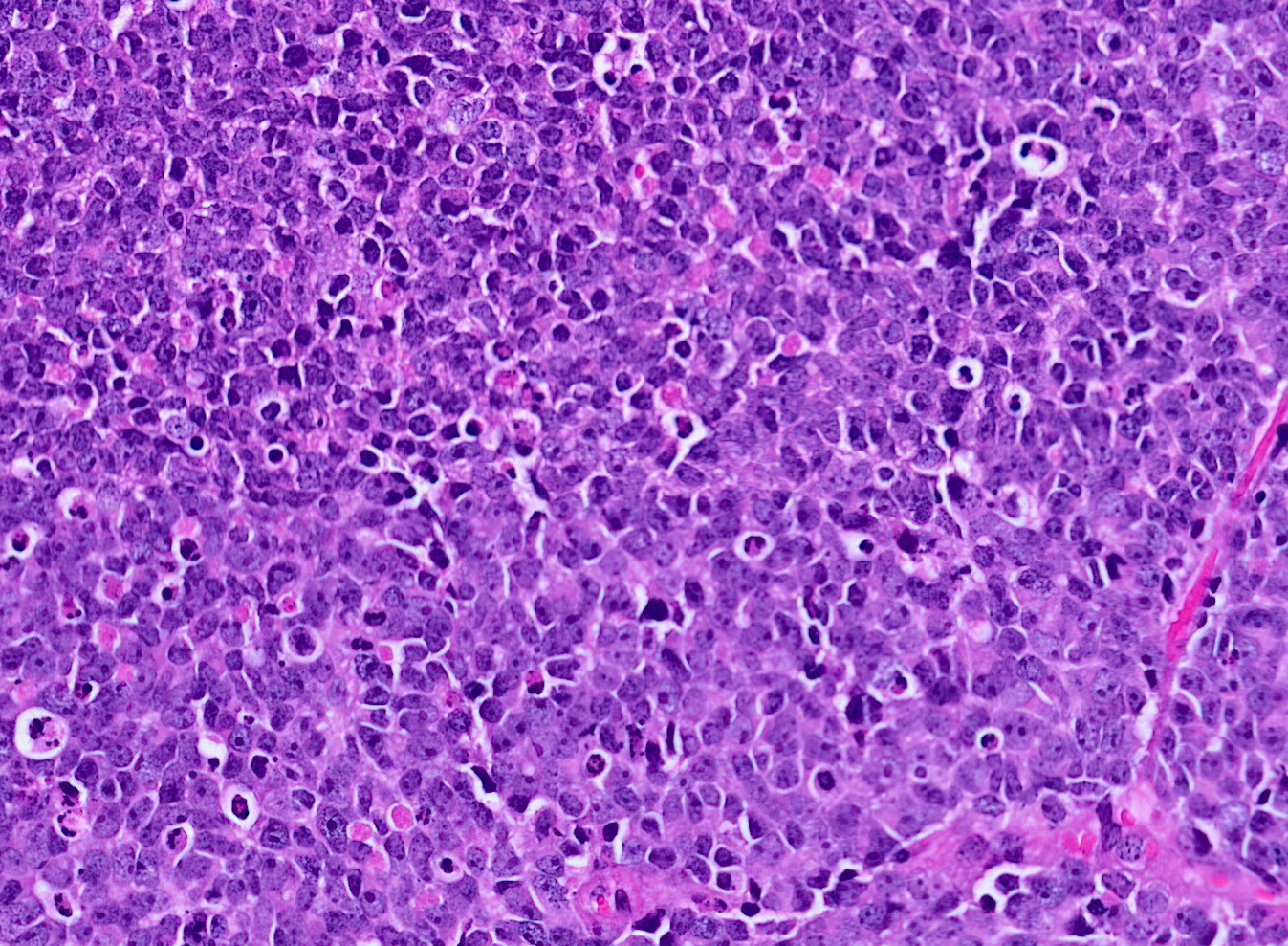

Supplement: Supplementary file 14 — Source data Fig. 5 [file 44320_2025_152_MOESM14_ESM.zip › Figure 5/5B/zccs373 Bioprinted HE x20.tif]

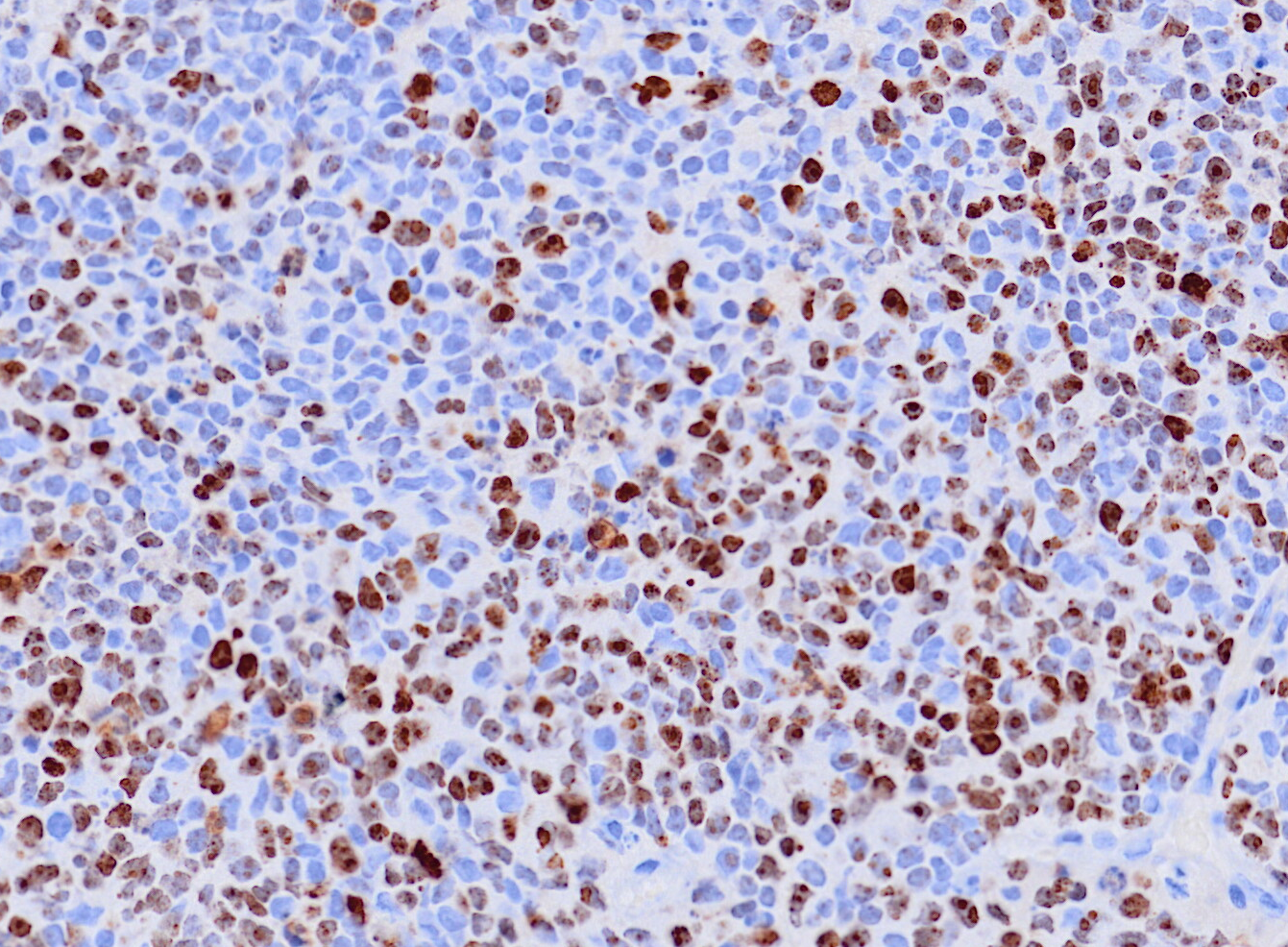

Supplement: Supplementary file 14 — Source data Fig. 5 [file 44320_2025_152_MOESM14_ESM.zip › Figure 5/5B/zccs373 Bioprinted Ki67 x20.tif]

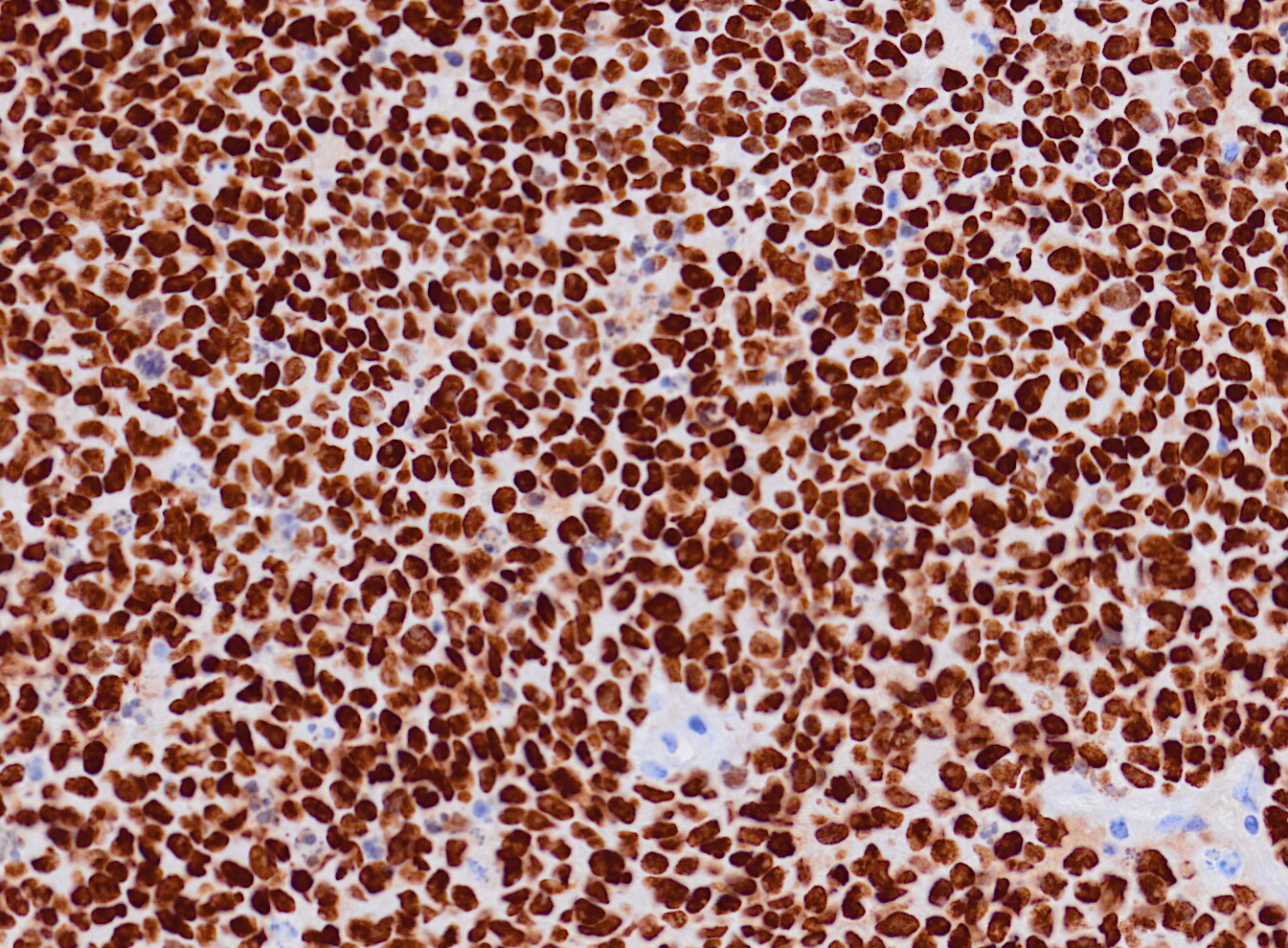

Supplement: Supplementary file 14 — Source data Fig. 5 [file 44320_2025_152_MOESM14_ESM.zip › Figure 5/5B/zccs373 Bioprinted PHOX2B x20.tif]

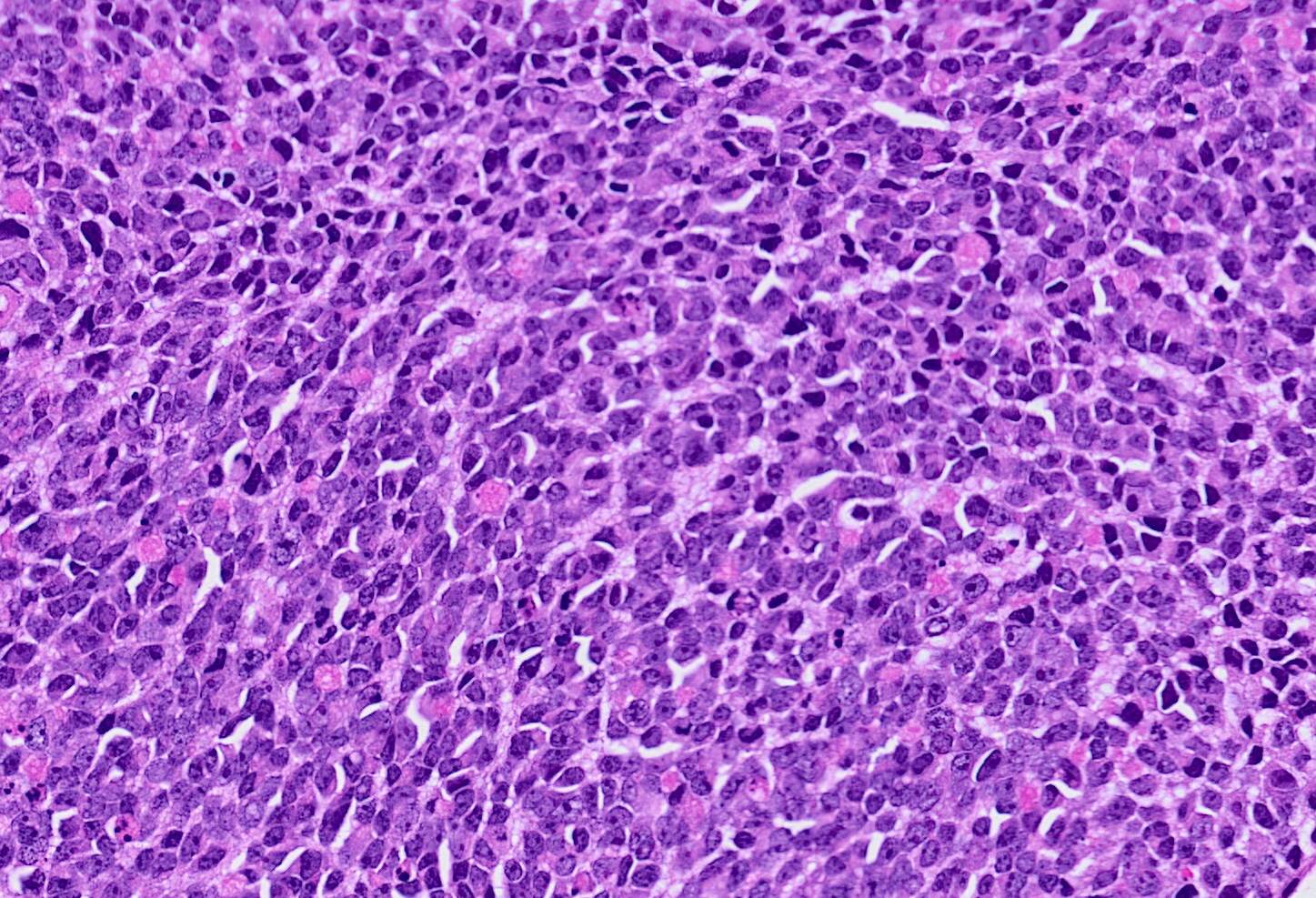

Supplement: Supplementary file 14 — Source data Fig. 5 [file 44320_2025_152_MOESM14_ESM.zip › Figure 5/5B/zccs373 Non-Bioprinted HE x20.tif]

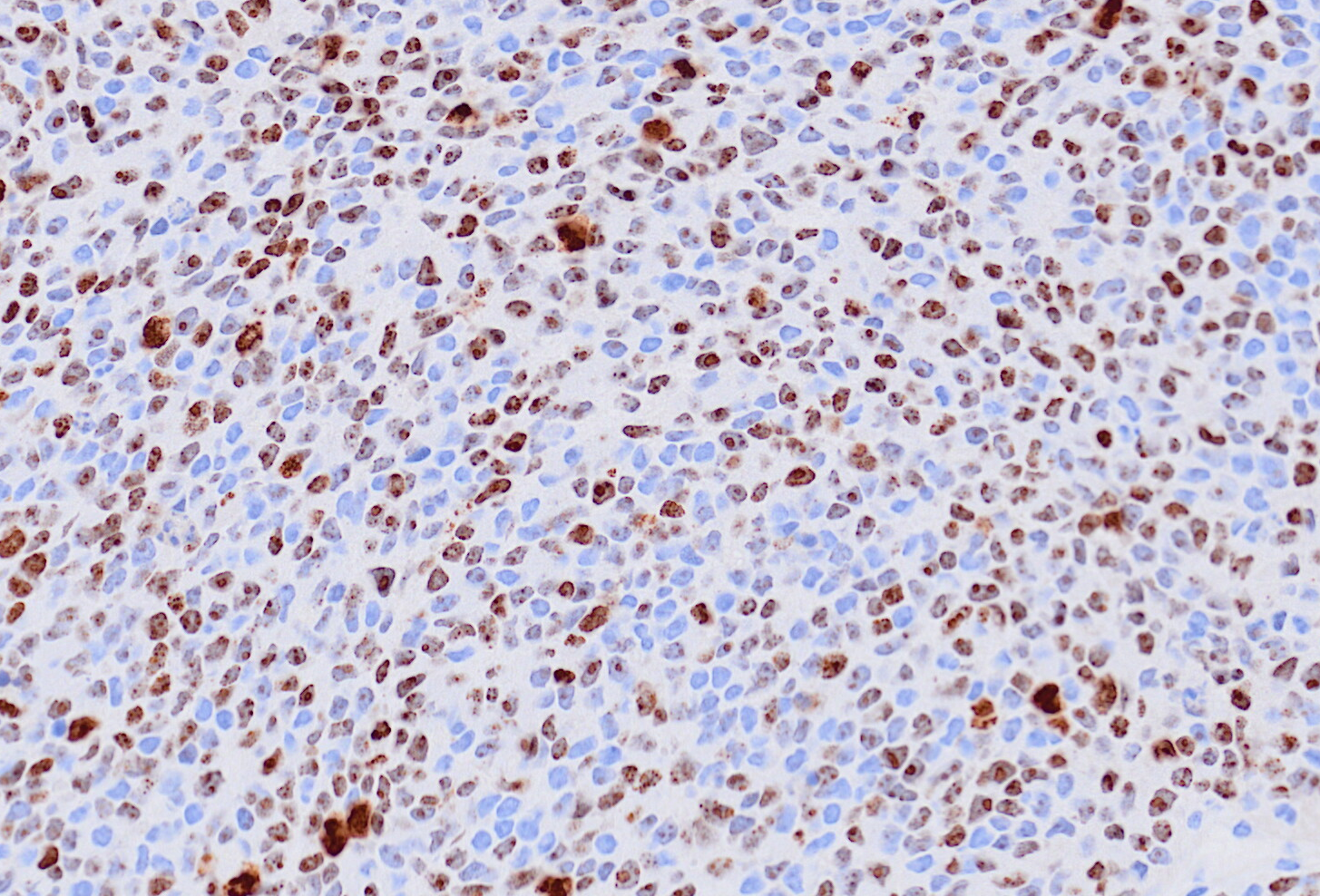

Supplement: Supplementary file 14 — Source data Fig. 5 [file 44320_2025_152_MOESM14_ESM.zip › Figure 5/5B/zccs373 Non-Bioprinted Ki67 x20.tif]

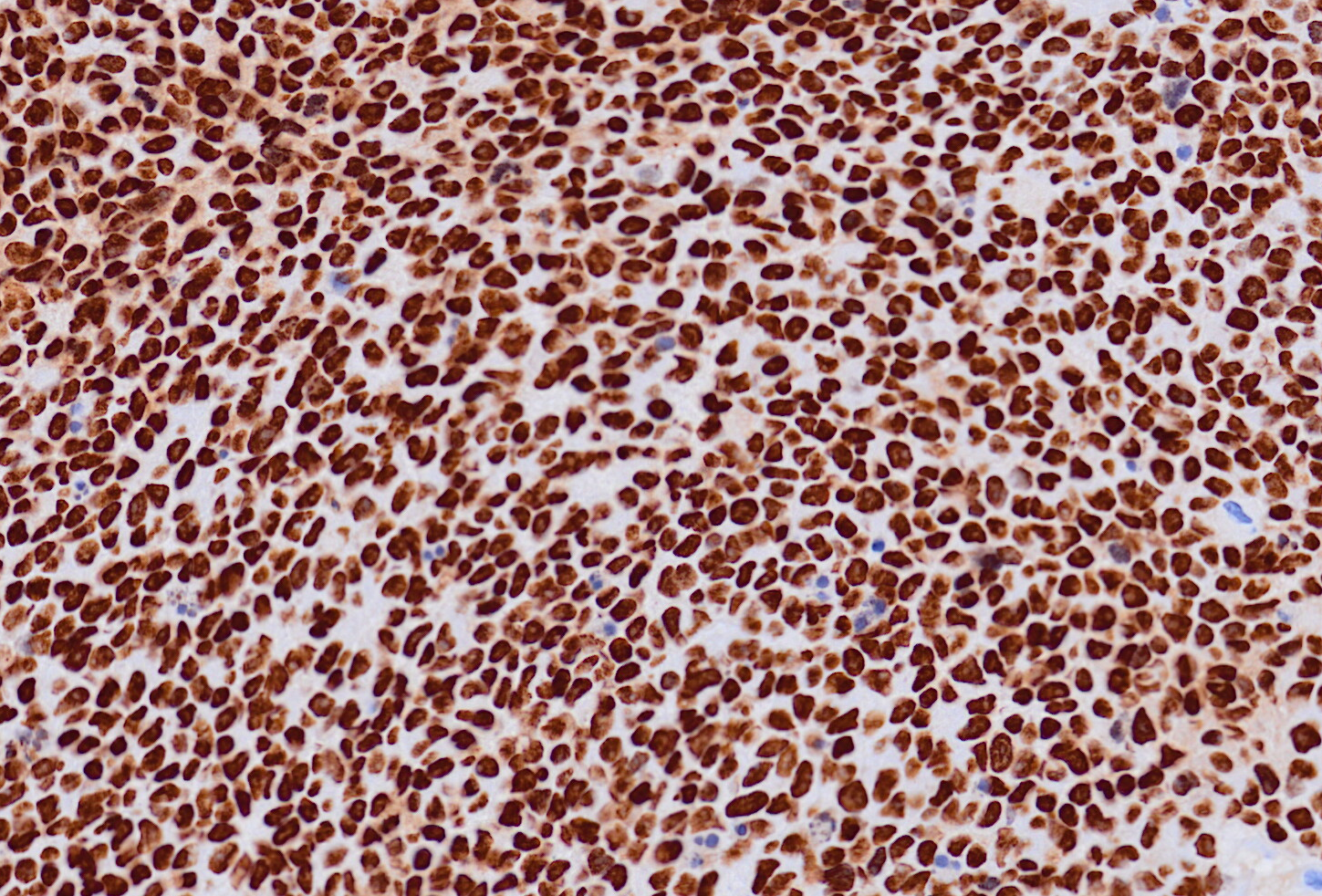

Supplement: Supplementary file 14 — Source data Fig. 5 [file 44320_2025_152_MOESM14_ESM.zip › Figure 5/5B/zccs373 Non-Bioprinted PHOX2B x20.tif]

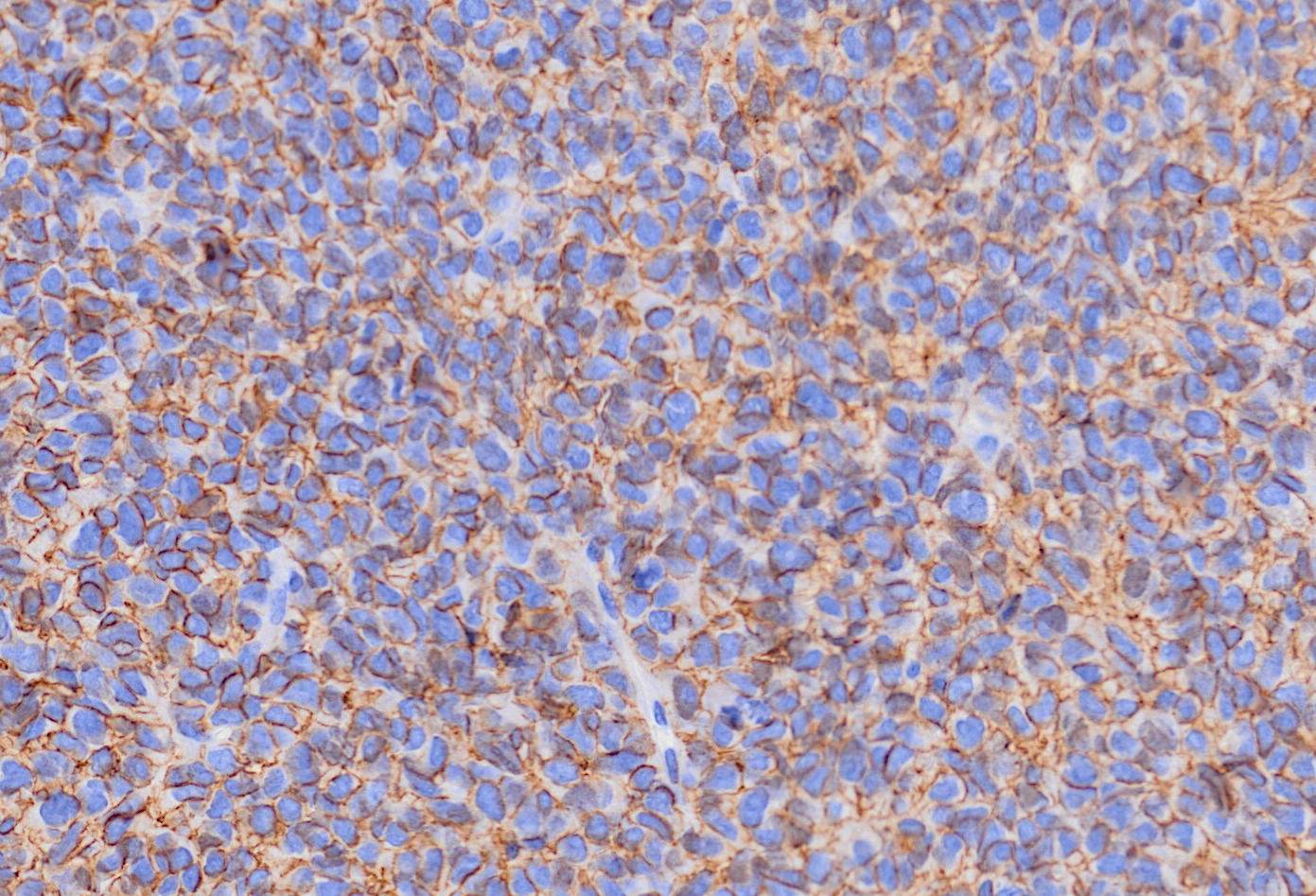

Supplement: Supplementary file 14 — Source data Fig. 5 [file 44320_2025_152_MOESM14_ESM.zip › Figure 5/5C/zccs207 Bioprinted CD99 x20.tif]

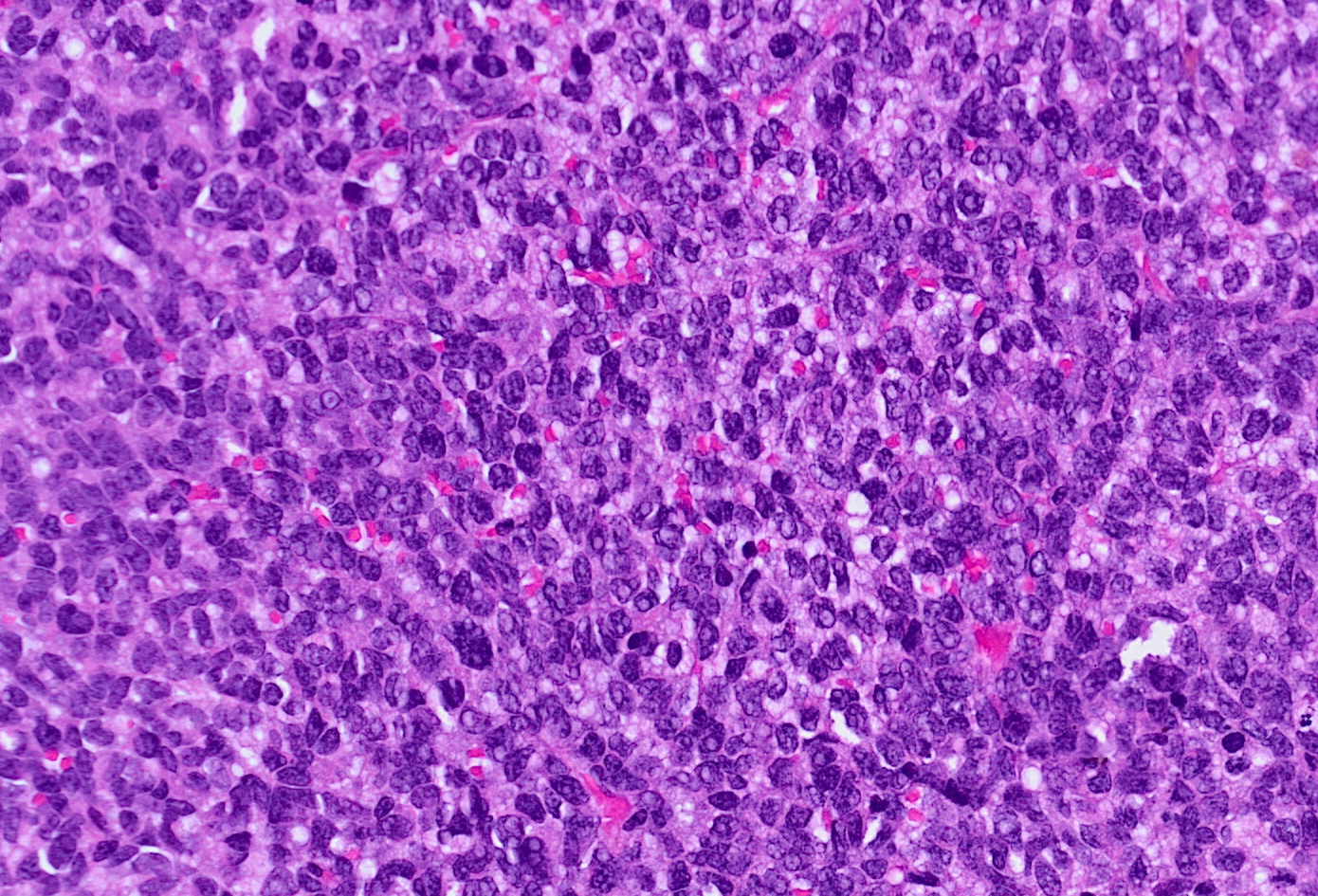

Supplement: Supplementary file 14 — Source data Fig. 5 [file 44320_2025_152_MOESM14_ESM.zip › Figure 5/5C/zccs207 Bioprinted HE x20.tif]

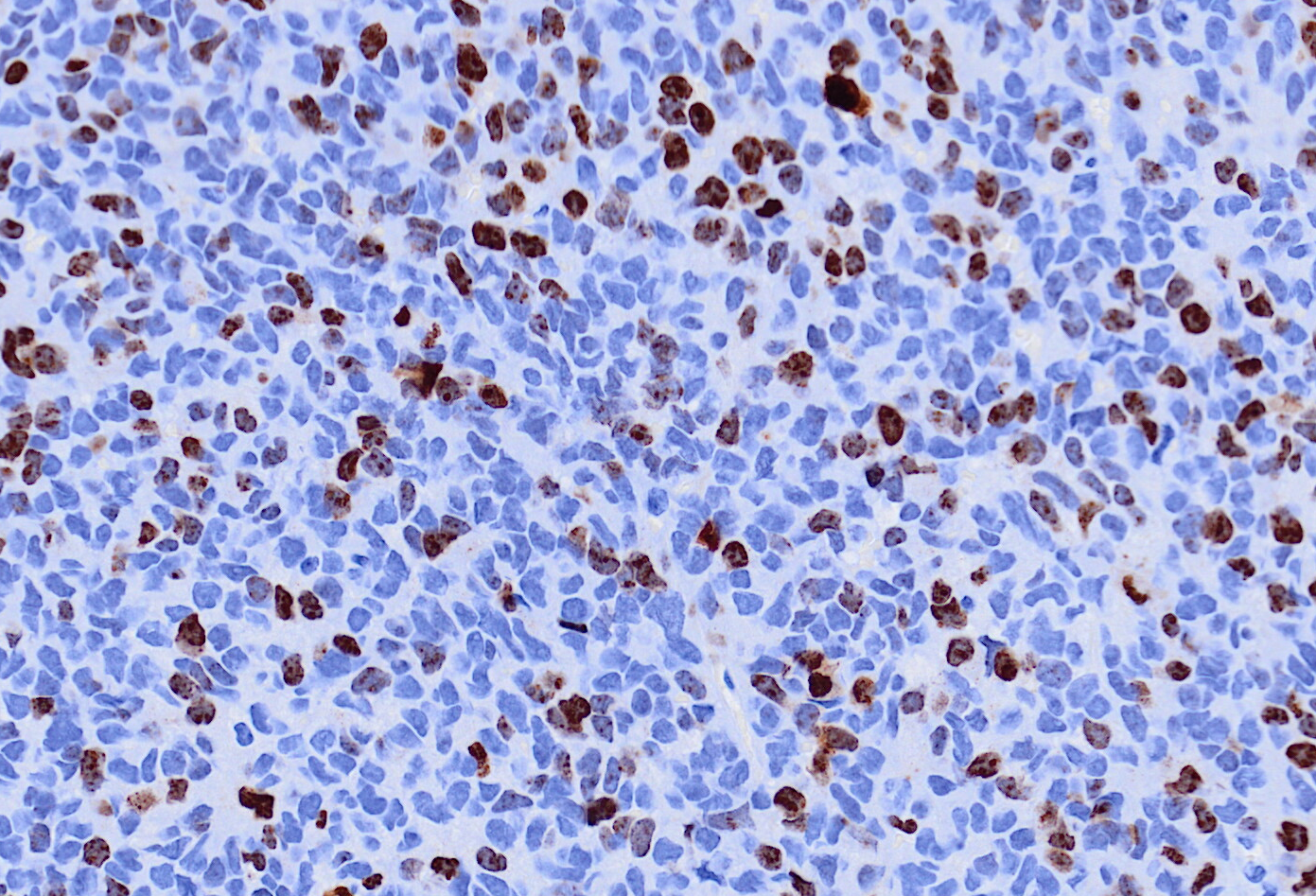

Supplement: Supplementary file 14 — Source data Fig. 5 [file 44320_2025_152_MOESM14_ESM.zip › Figure 5/5C/zccs207 Bioprinted Ki67 x20.tif]

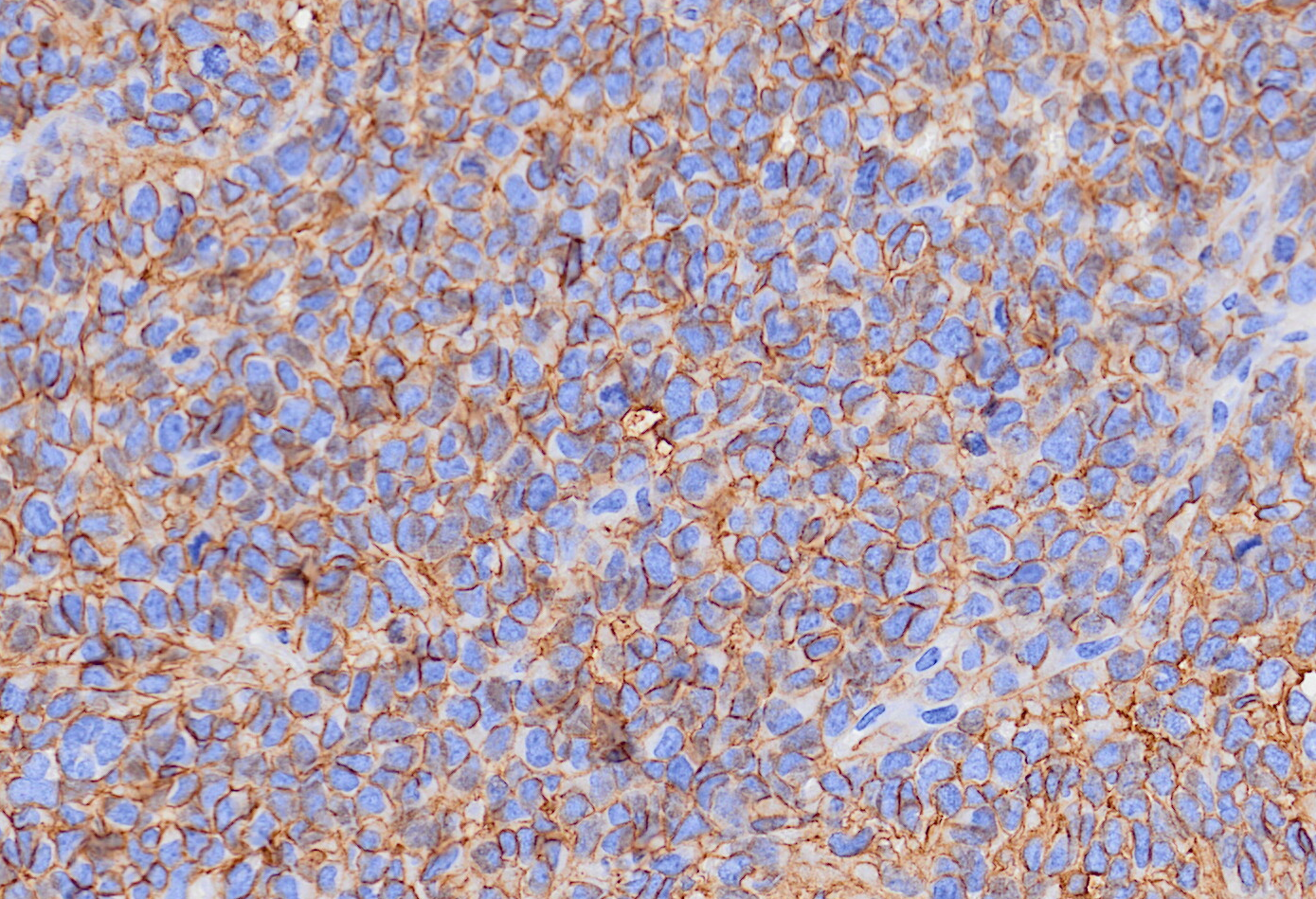

Supplement: Supplementary file 14 — Source data Fig. 5 [file 44320_2025_152_MOESM14_ESM.zip › Figure 5/5C/zccs207 Non-Bioprinted CD99 x20.tif]

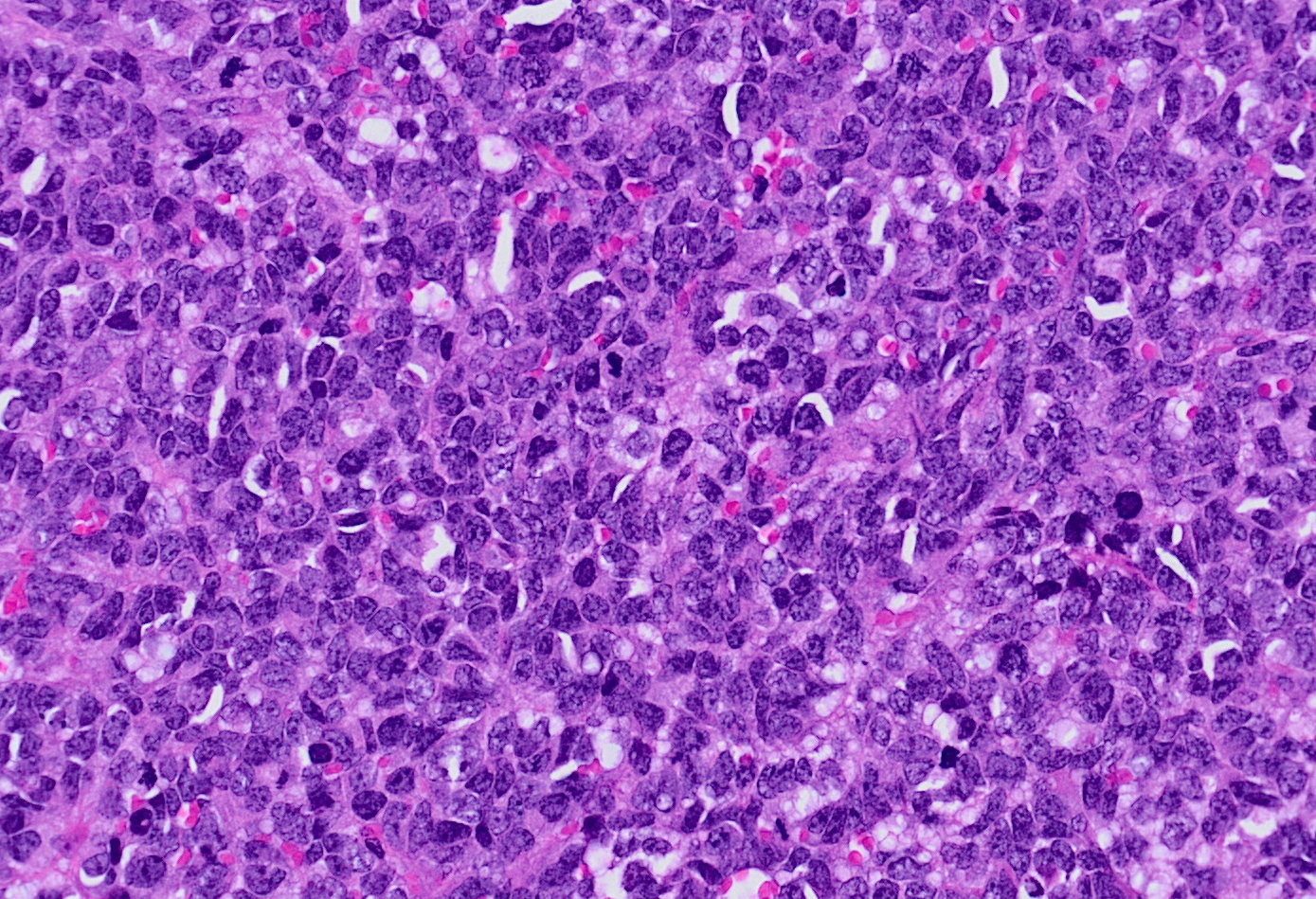

Supplement: Supplementary file 14 — Source data Fig. 5 [file 44320_2025_152_MOESM14_ESM.zip › Figure 5/5C/zccs207 Non-Bioprinted HE x20.tif]

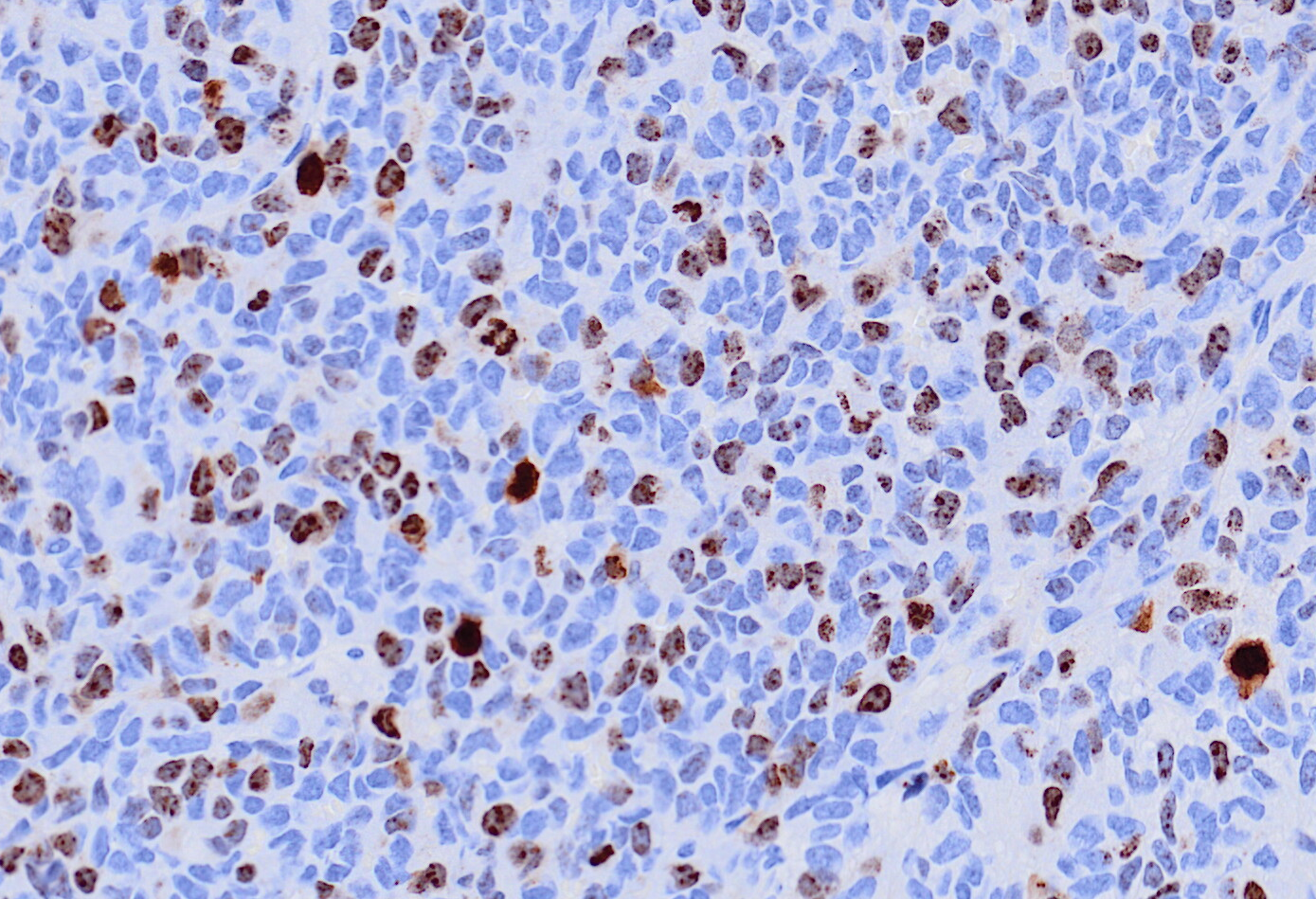

Supplement: Supplementary file 14 — Source data Fig. 5 [file 44320_2025_152_MOESM14_ESM.zip › Figure 5/5C/zccs207 Non-Bioprinted Ki67 x20.tif]

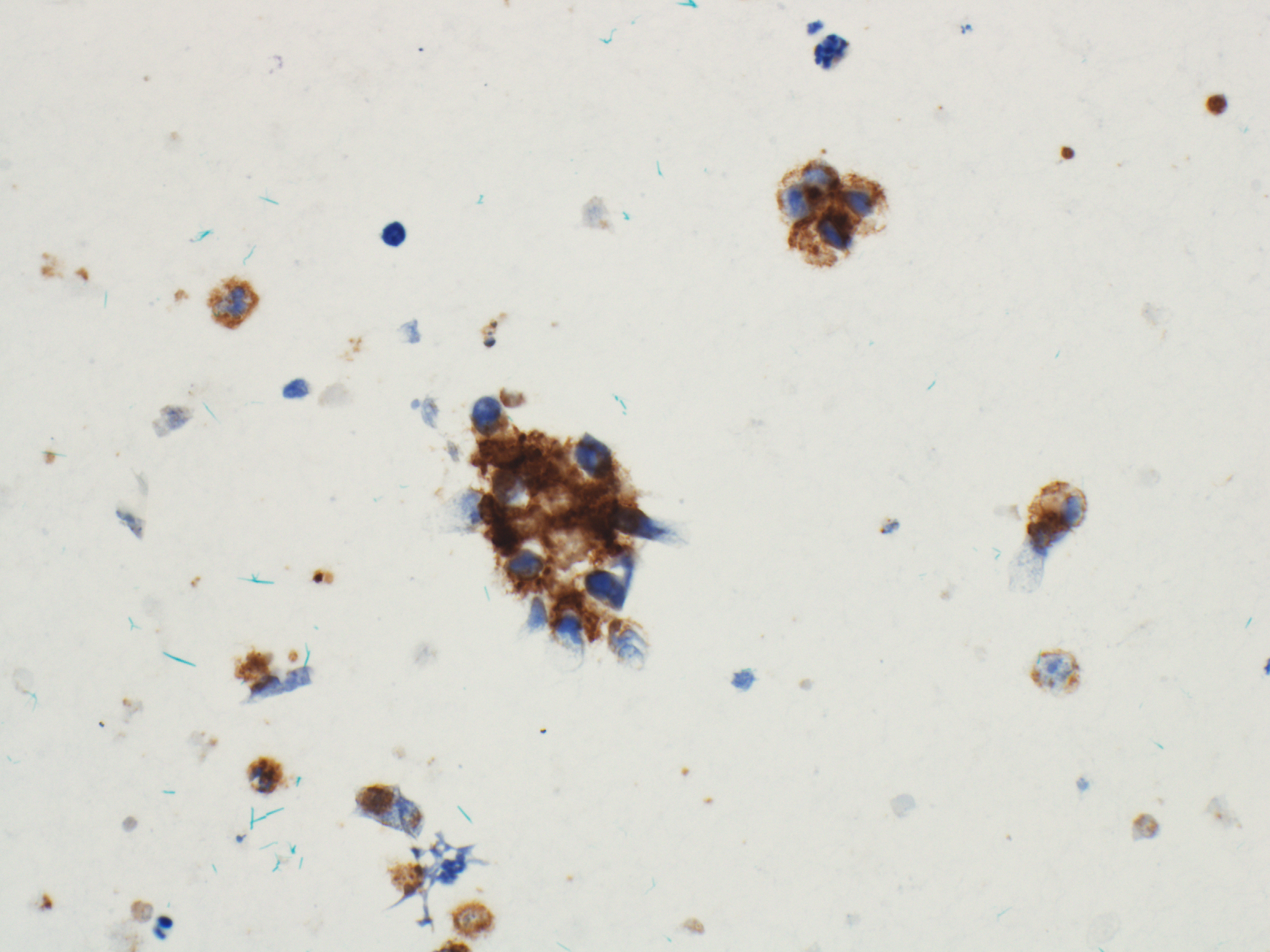

Supplement: Supplementary file 19 — Figure EV6 Source Data [file 44320_2025_152_MOESM19_ESM.zip › Supp Figure 6/Supp Fig 6F/zccs486 CD99 x60.tif]

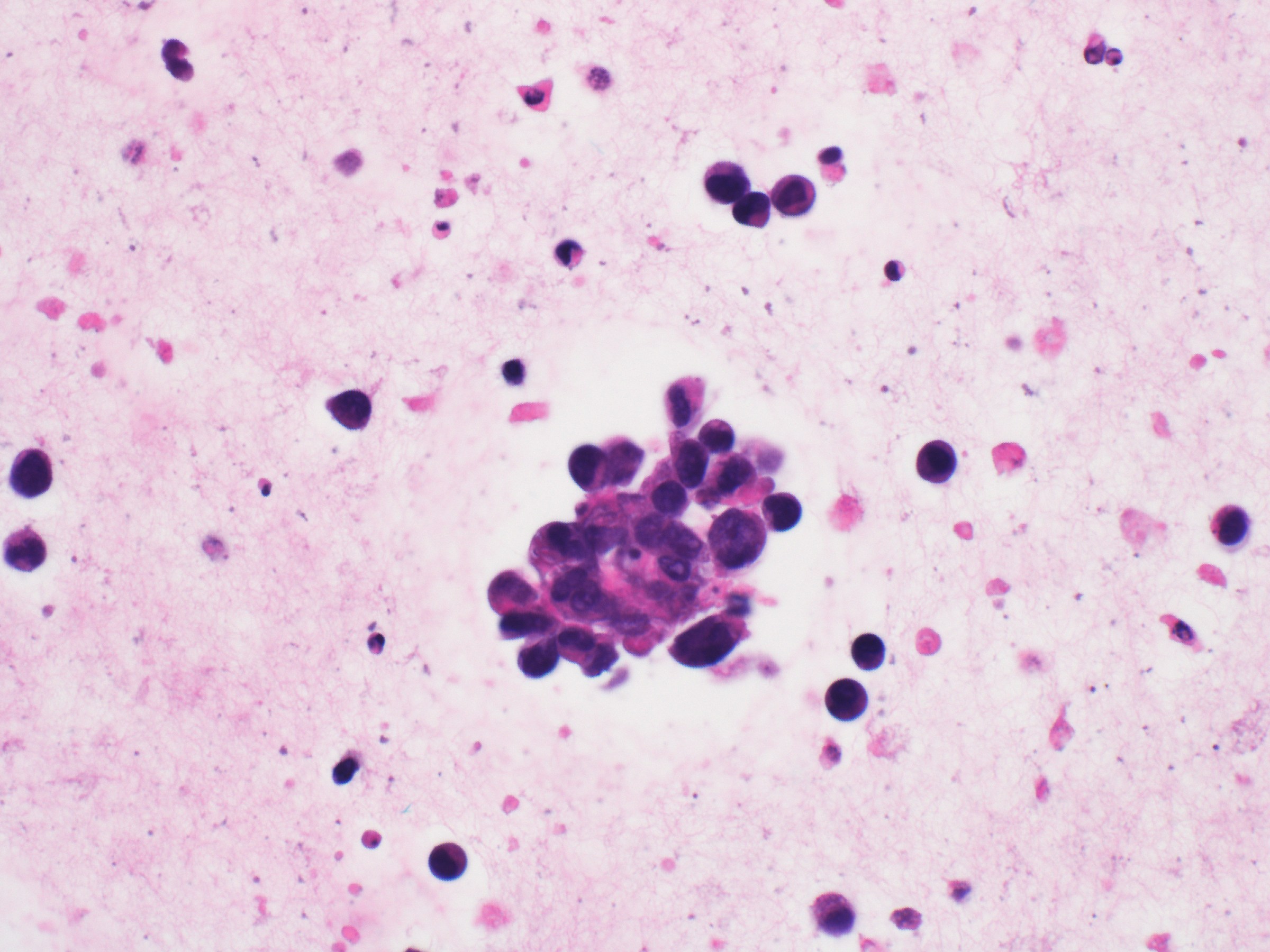

Supplement: Supplementary file 19 — Figure EV6 Source Data [file 44320_2025_152_MOESM19_ESM.zip › Supp Figure 6/Supp Fig 6F/zccs486 HE x60.tif]

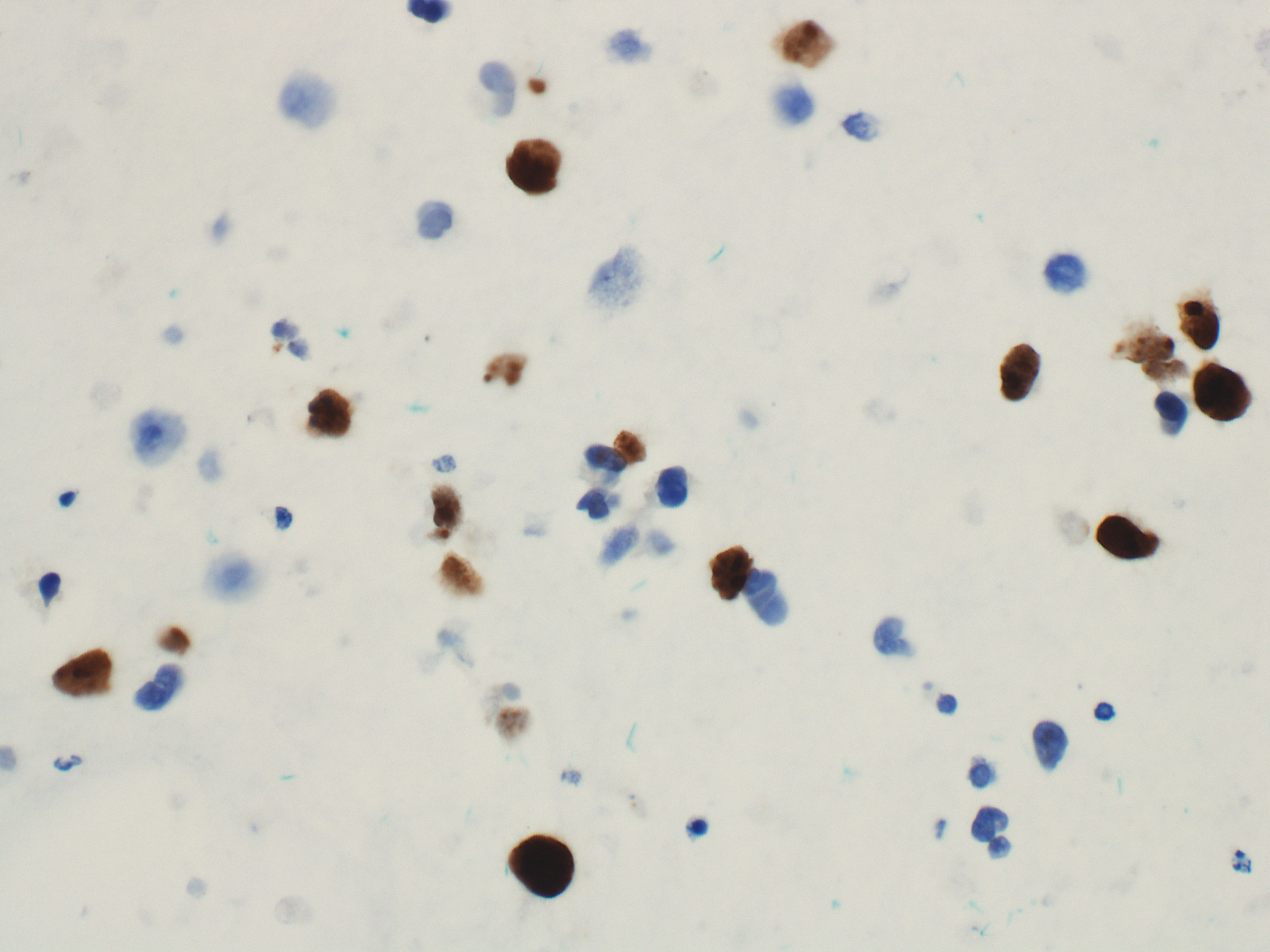

Supplement: Supplementary file 19 — Figure EV6 Source Data [file 44320_2025_152_MOESM19_ESM.zip › Supp Figure 6/Supp Fig 6F/zccs486 Ki67 x60.tif]

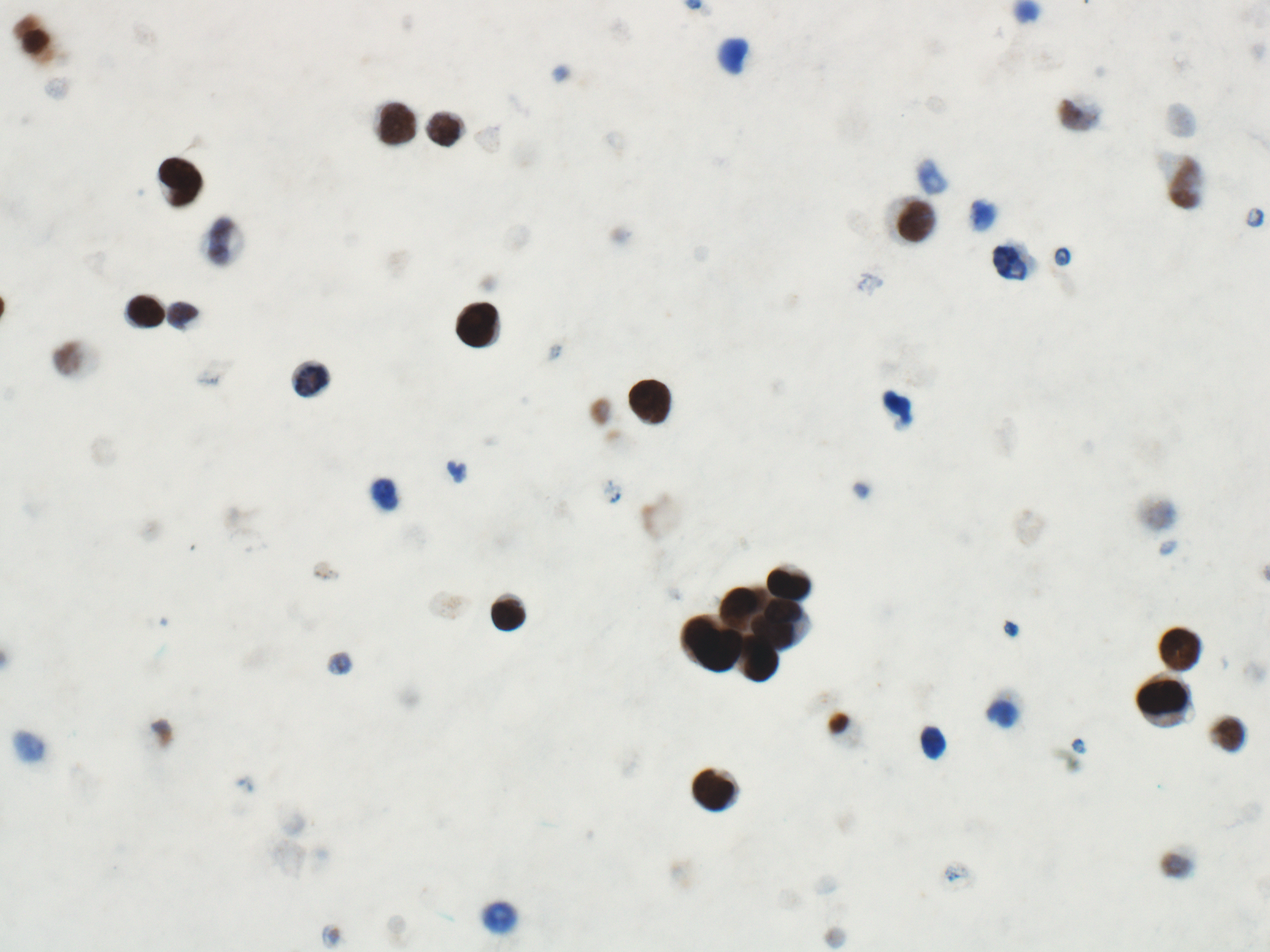

Supplement: Supplementary file 19 — Figure EV6 Source Data [file 44320_2025_152_MOESM19_ESM.zip › Supp Figure 6/Supp Fig 6F/zccs486 NKX2.2 x60.tif]
