# Supplementary material for: High-throughput 3D engineered paediatric tumour models for precision medicine
Source: Mol Syst Biol. 2025 Oct 1;21(12):1748–77. doi: 10.1038/s44320-025-00152-y (PMC12673126; doi:10.1038/s44320-025-00152-y)
Supplement: Supplementary file 20 — Expanded View Figures [file 44320_2025_152_MOESM20_ESM.pdf]

Expanded View Figures

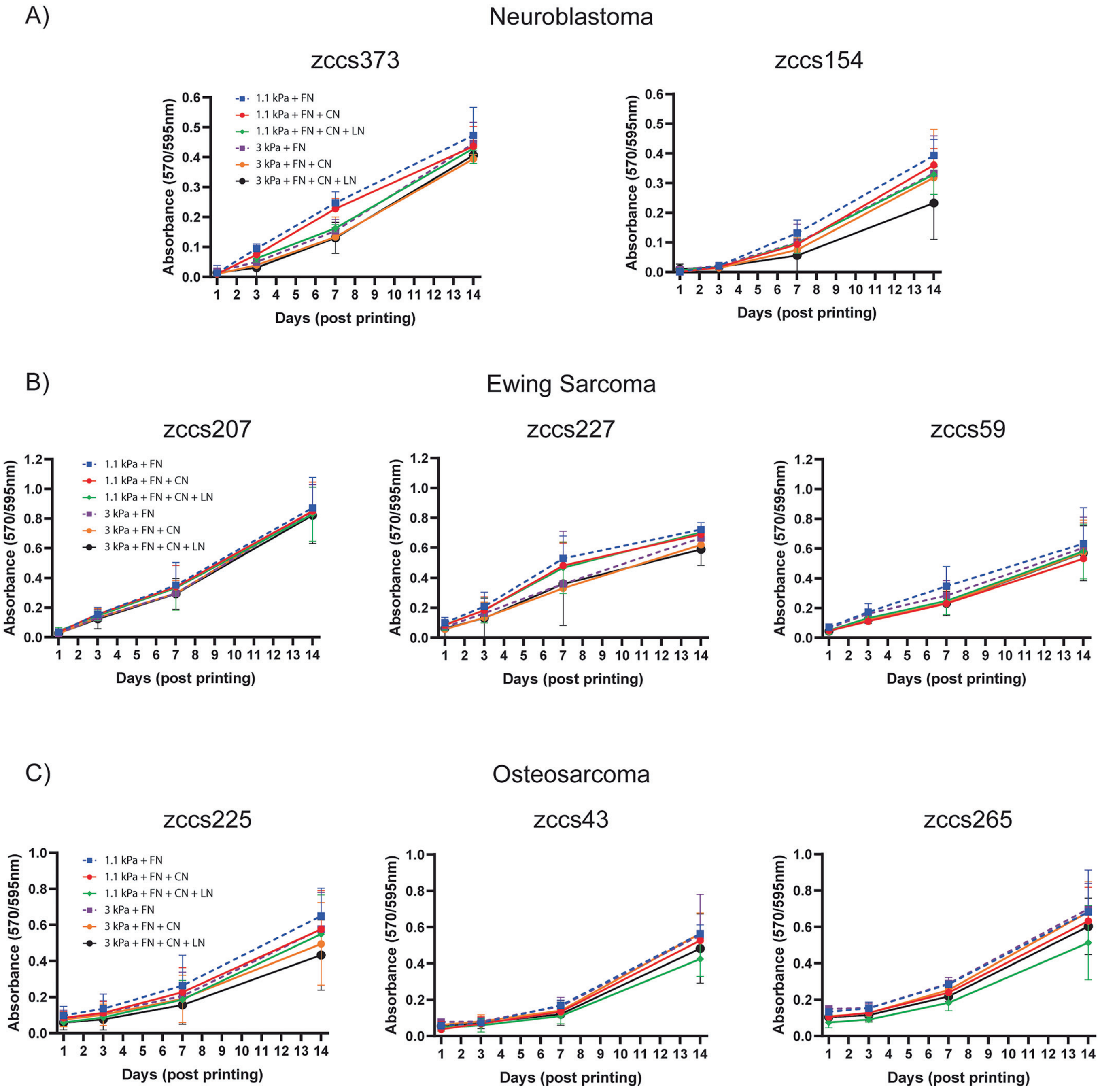

Figure EV1. Cell proliferation of 3D bioprinted PDX tumouroids.

Cell proliferation graphs of 3D bioprinted patient-derived PDX cells in the six hydrogel conditions. Each sample was bioprinted in either 1.1 kPa or 3 kPa hydrogels, containing fibronectin (FN) only, FN and collagen (CN), or FN, CN and laminin (LN) peptides. Proliferation rate was measured at day 1, 3, 7 and 14 post-printing. Graphs are separated into cancer types (**A**) neuroblastoma, (**B**) Ewing sarcoma and (**C**) osteosarcoma. Data are presented as mean ± SD, n.s. $P > 0.9$ (one-way ANOVA with Tukey's multiple comparisons test for each disease group). All experiments were repeated three times, except for zccs227, which had limited sample availability and is $n = 2$. Related to Fig. 2. Source data are available online for this figure.

 © The Author(s)

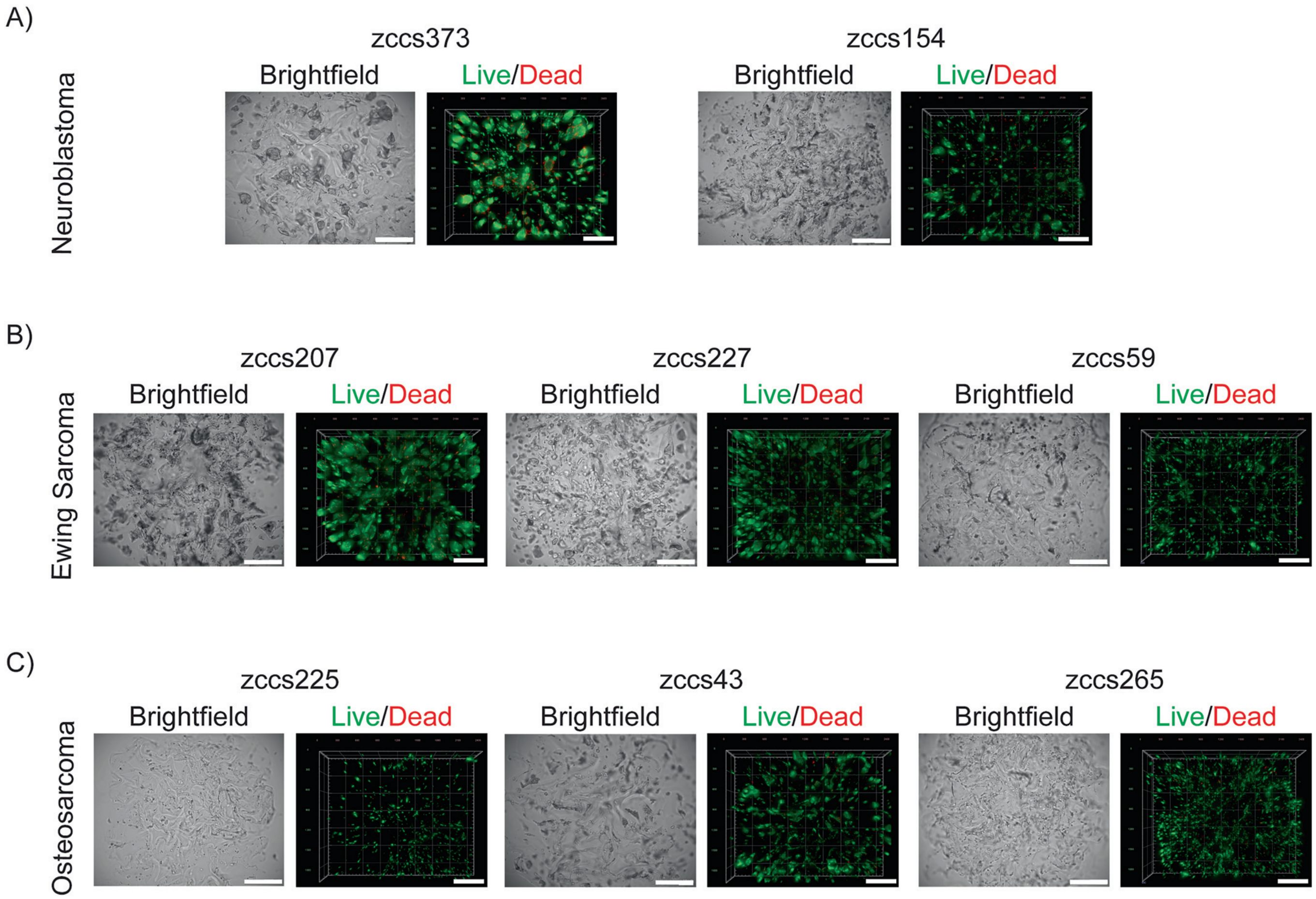

Figure EV2. Cell viability of 3D bioprinted PDX tumouroids.

Each sample was bioprinted in 3 kPa + FN + CN + LN hydrogels and cultured up to 14 days. Graphs are separated into cancer types (**A**) neuroblastoma, (**B**) Ewing sarcoma and (**C**) osteosarcoma. Cells were stained with calcein-AM (green; live)/ethidium homodimer 1 (red; dead) Live/Dead Assay. Z stack 3D images were taken at day 14 post-printing. Representative image shown for each sample in brightfield (left) and Live/Dead (right). Scale bars on all images are 500 μm. Related to Fig. 2.

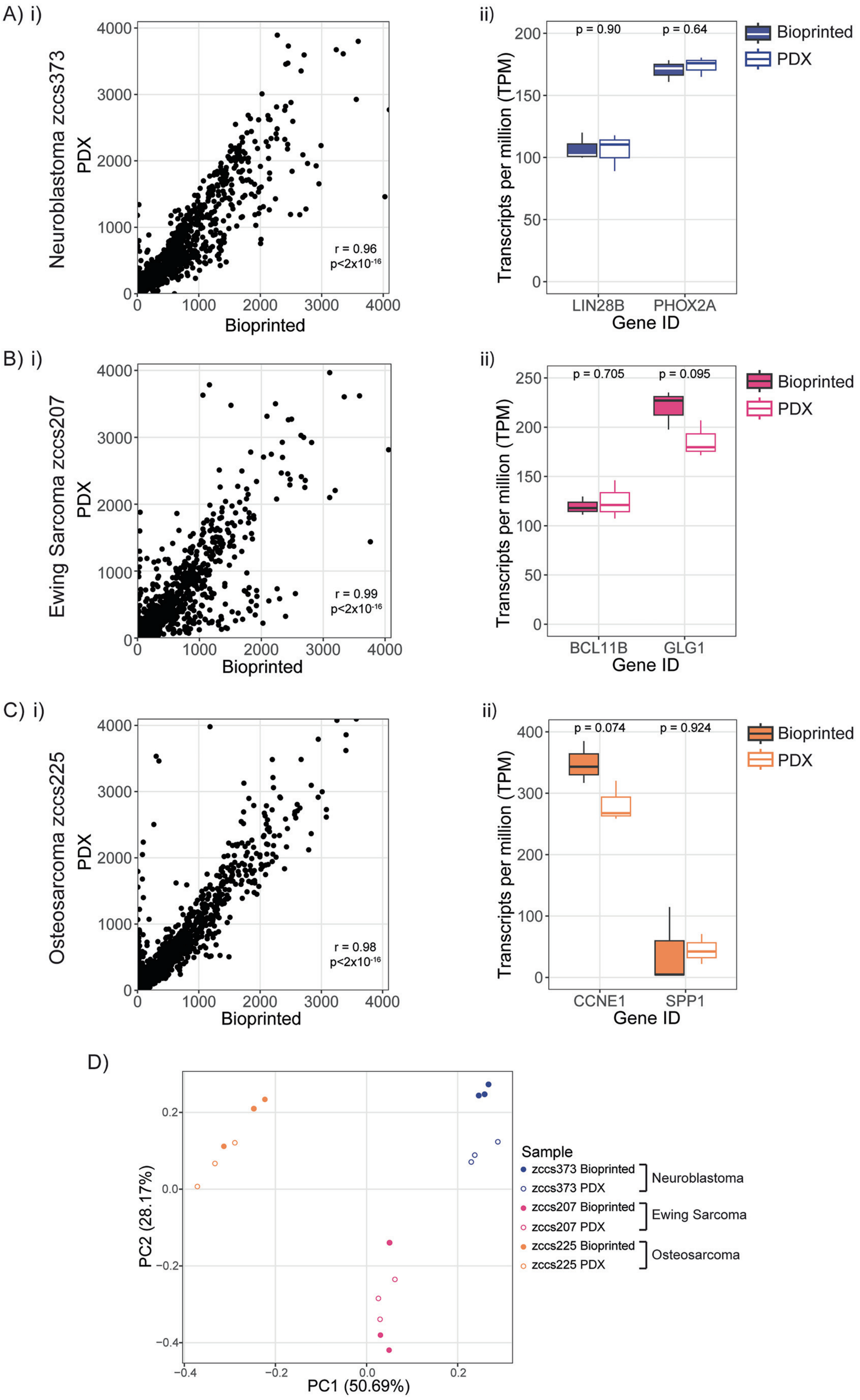
A) i)
Neuroblastoma zccs373
PDX
Bioprinted
r = 0.96
p<2x10-16
ii)
Transcripts per million (TPM)
p = 0.90
p = 0.64
LIN28B
PHOX2A
Gene ID
Bioprinted
PDX
B) i)
Ewing Sarcoma zccs207
PDX
Bioprinted
r = 0.99
p<2x10-16
ii)
p = 0.705
p = 0.095
BCL11B
GLG1
C) i)
Osteosarcoma zccs225
r = 0.98
p<2x10-16
ii)
p = 0.074
p = 0.924
CCNE1
SPP1
D)
PC2 (28.17%)
PC1 (50.69%)
Sample
zccs373 Bioprinted
zccs373 PDX
Neuroblastoma
zccs207 Bioprinted
zccs207 PDX
Ewing Sarcoma
zccs225 Bioprinted
zccs225 PDX
Osteosarcoma

 © The Author(s)

Figure EV3. Comparative RNAseq analysis of PDX cells and bioprinted PDX tumouroids.

(**A–Ci**) Scatterplot showing gene expression correlation between PDX and a matched bioprinted tumouroid sample for neuroblastoma zccs373, Ewing sarcoma zccs207 and osteosarcoma zccs225. Each dot represents a gene expression value pair in transcripts per million (TPM). Pearson's correlation analysis, *P* values (<2x10e-16) are indicated on corresponding plots. (**A–Cii**) Box plots showing individual transcript level (TPM) between bioprinted and PDX samples for LIN28B and PHOX2B in zcc373; BLC11B and GLG1 in zccs207 and CCNE1 and SPP1 in zccs225. Data are presented as median ±IQR; *P* values are indicated on corresponding plots. (**D**) Principal component analysis plot showing transcriptomic profiles of PDX and matched bioprinted samples across three tumour types. PC1 and PC2 account for 28.17% and 50.69% variance, respectively. Related to Fig. 3.

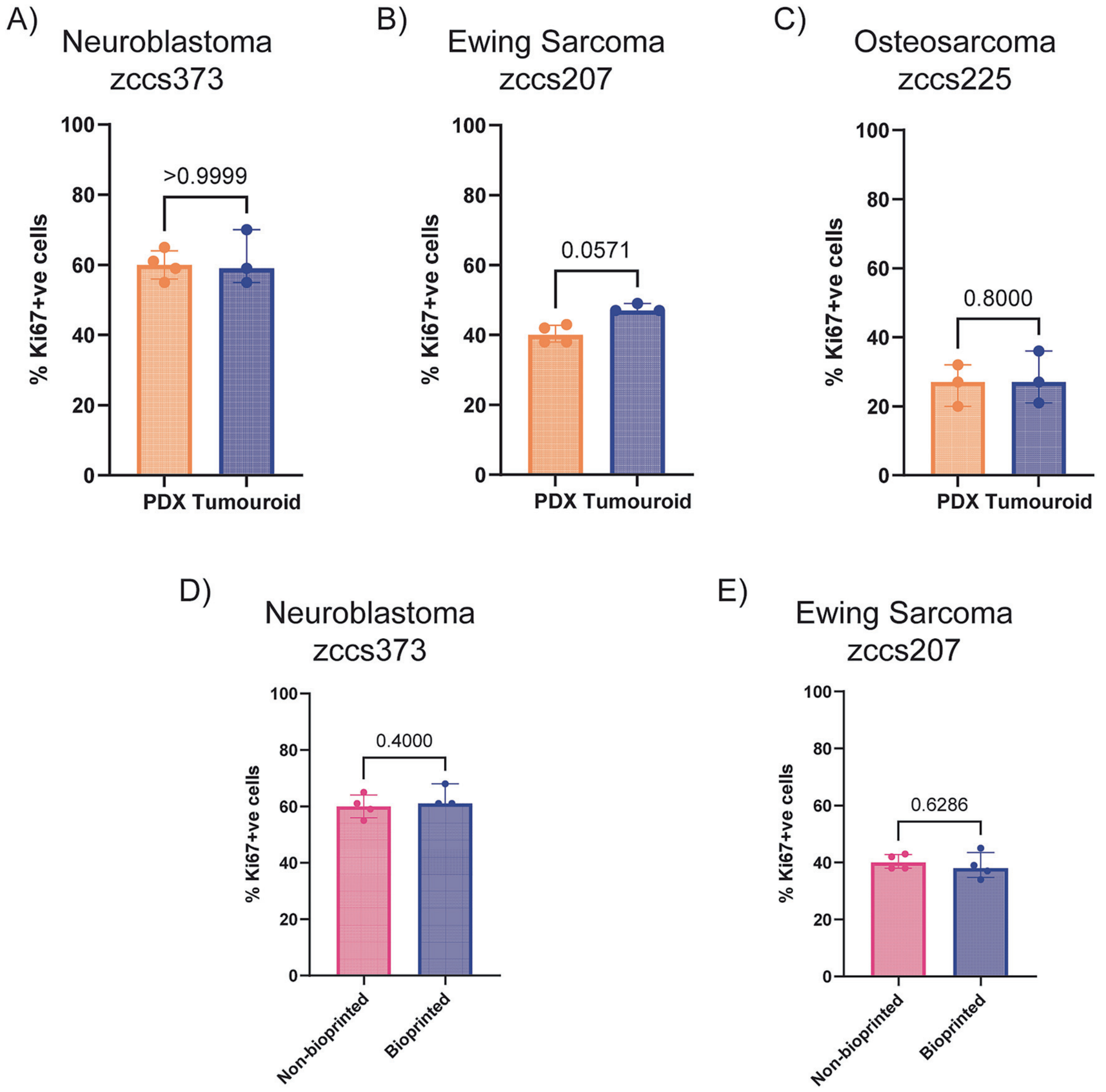

Figure EV4. Cellular proliferation remains unchanged after the bioprinting process in vitro and in vivo.

(**A–C**) Quantification of % Ki-67 positive cells in PDX tissues and bioprinted tumouroids from neuroblastoma zccs373, Ewing sarcoma zccs207 and osteosarcoma zccs225 samples. (**D**, **E**) Quantification of % Ki-67 positive cells in PDX tissues from non-bioprinted and bioprinted PDX in the tumourigenicity study. For PDX tumours, each dot represents an individual tumour (3 ROI averaged per tumour, $n = 3$–4 tumours). For PDX-derived tumouroids, each dot represents individual structures ($n = 3$). Data are presented as median ±IQR; Mann–Whitney *U* test; Adjusted *P* values are displayed in the figure panel. Related to Figs. 4 and 5. Source data are available online for this figure.

 © The Author(s)

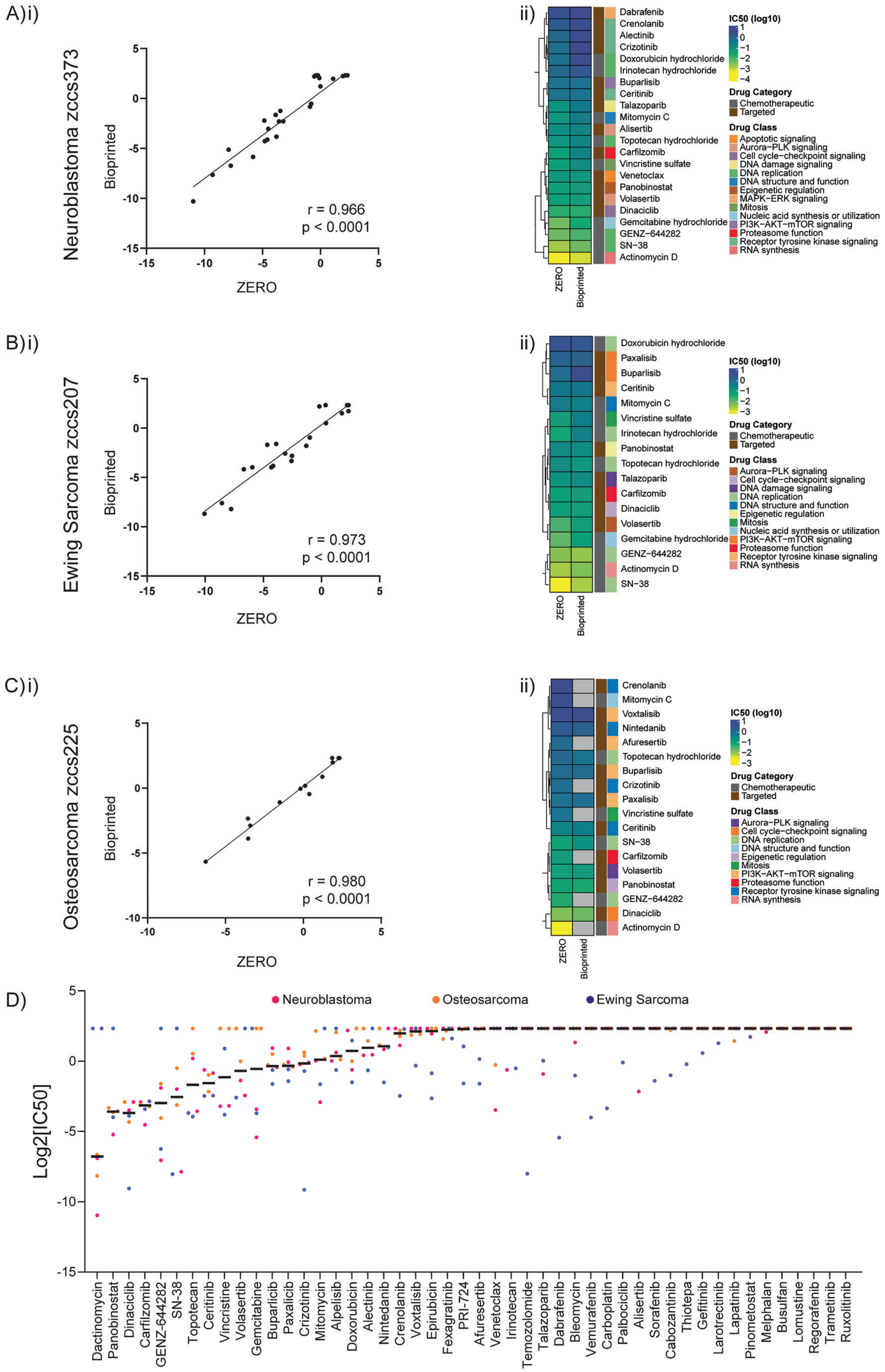
A)i)
Neuroblastoma zccs373
Bioprinted
ZERO
r = 0.966
p < 0.0001
ii)
Dabrafenib
Crenolanib
Alectinib
Crizotinib
Doxorubicin hydrochloride
Irinotecan hydrochloride
Buparlisib
Ceritinib
Talazoparib
Mitomycin C
Alisertib
Topotecan hydrochloride
Carfilzomib
Vincristine sulfate
Venetoclax
Panobinostat
Volasertib
Dinaciclib
Gemcitabine hydrochloride
GENZ−644282
SN−38
Actinomycin D
IC50 (log10)
Drug Category
Chemotherapeutic
Targeted
Drug Class
Apoptotic signaling
Aurora−PLK signaling
Cell cycle−checkpoint signaling
DNA damage signaling
DNA replication
DNA structure and function
Epigenetic regulation
MAPK−ERK signaling
Mitosis
Nucleic acid synthesis or utilization
PI3K−AKT−mTOR signaling
Proteasome function
Receptor tyrosine kinase signaling
RNA synthesis
B)i)
Ewing Sarcoma zccs207
r = 0.973
p < 0.0001
Paxalisib
C)i)
Osteosarcoma zccs225
r = 0.980
p < 0.0001
Voxtalisib
Nintedanib
Afuresertib
D)
Neuroblastoma
Osteosarcoma
Ewing Sarcoma
Log2[IC50]
Dactinomycin
Panobinostat
Dinaciclib
Carfilzomib
GENZ-644282
SN-38
Topotecan
Ceritinib
Vincristine
Volasertib
Gemcitabine
Buparlicib
Paxalicib
Crizotinib
Mitomycin
Alpelisib
Doxorubicin
Alectinib
Nintedanib
Crenolanib
Voxtalisib
Epirubicin
Fexagratinib
PRI-724
Afuresertib
Venetoclax
Irinotecan
Temozolomide
Talazoparib
Dabrafenib
Bleomycin
Vemurafenib
Carboplatin
Palbociclib
Alisertib
Sorafenib
Cabozantinib
Thiotepa
Gefitinib
Larotrectinib
Lapatinib
Pinometostat
Melphalan
Busulfan
Lomustine
Regorafenib
Trametinib
Ruxolitinib

Figure EV5. Comparison of HTP drug screening approaches between preclinical testing and 3D bioprinting workflow.

(**A–C**) (i) Correlation between ZERO and 3D bioprinting workflow for three PDX samples. Scatterplot with line of best fit. Each dot represents a log2[IC_{50}] value for a specific drug for both conditions. $r > 0.9$, Pearson's correlation analysis, P values (<0.0001) are indicated on corresponding plots. (**A–C**) (ii) Heatmap visualisation comparing log10[IC50] values for two approaches across chemotherapeutic and targeted drug classes. IC_{50} values > 5 were excluded to assist with visualisation. Grey boxes indicate unavailable IC_{50} values for zccs225 due to technical limitations at the time of the experiment. (**D**) Log2[IC_{50}] distribution across all samples for a 48-drug library. Drugs were ordered based on the lowest to highest median Log2[IC_{50}] values, followed by lowest quartile and then lowest detected Log2[IC_{50}] values. Related to Fig. 6. Source data are available online for this figure.

 © The Author(s)

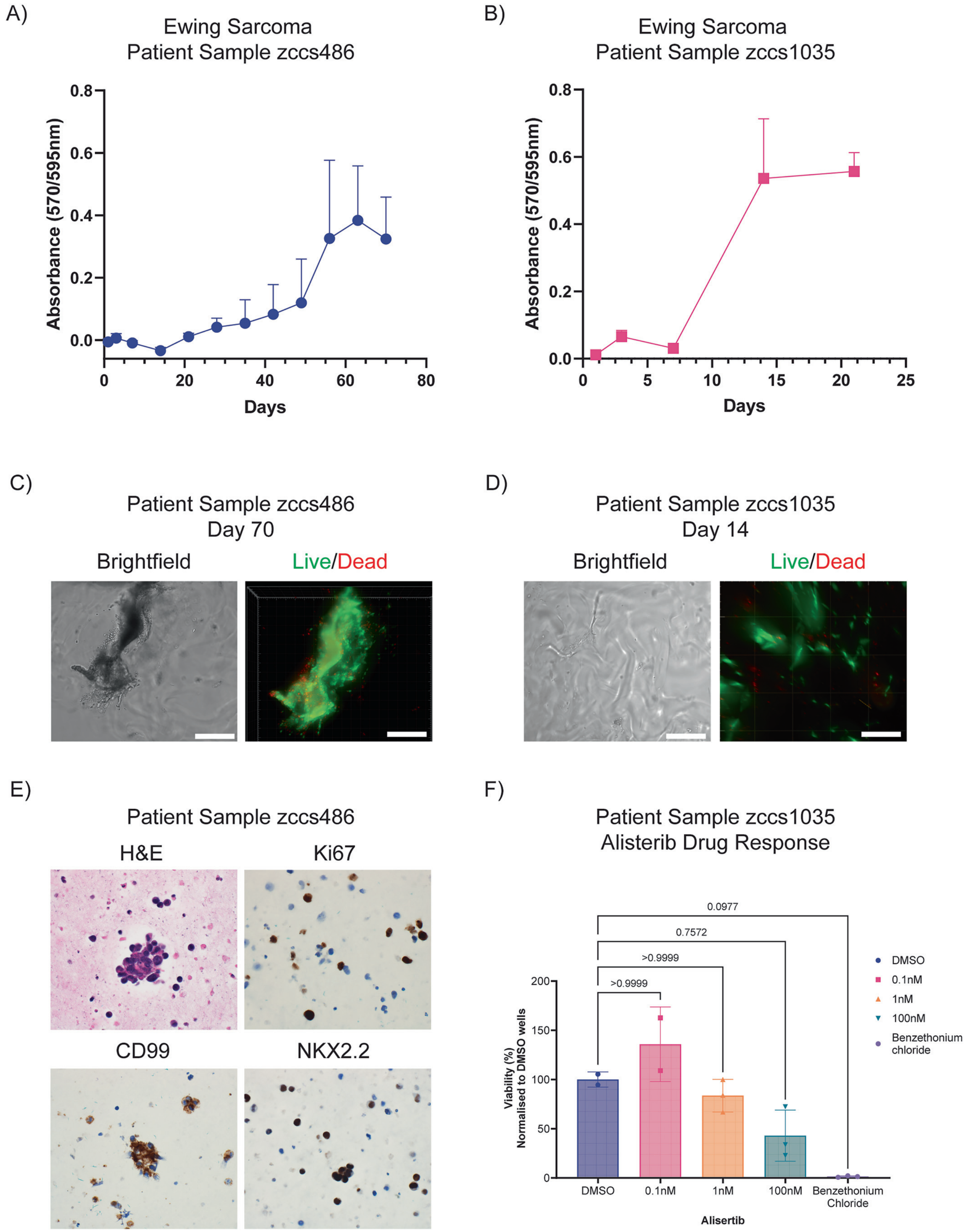
A)
Ewing Sarcoma
Patient Sample zccs486
Absorbance (570/595nm)
0.8
0.6
0.4
0.2
0.0
0
20
40
60
80
Days
B)
Ewing Sarcoma
Patient Sample zccs1035
Absorbance (570/595nm)
0.8
0.6
0.4
0.2
0.0
0
5
10
15
20
25
Days
C)
Patient Sample zccs486
Day 70
Brightfield
Live/Dead
D)
Patient Sample zccs1035
Day 14
Brightfield
Live/Dead
E)
Patient Sample zccs486
H&E
Ki67
CD99
NKX2.2
F)
Patient Sample zccs1035
Alisterib Drug Response
0.0977
0.7572
>0.9999
>0.9999
Viability (%)
Normalised to DMSO wells
200
150
100
50
0
DMSO
0.1nM
1nM
100nM
Benzethonium Chloride
Alisertib
DMSO
0.1nM
1nM
100nM
Benzethonium chloride

Figure EV6. Compatibility of original patient samples with 3D bioprinting for sample expansion and drug screening.

(**A**, **B**) Cell proliferation of 3D bioprinted patient cells for zccs486 and zccs1035. Data are presented as mean ± SD. (**C**, **D**) Representative brightfield (left) and live/dead images (right) for 3D bioprinted patient tumouroids at day 70 (zccs486) and day 14 (zccs1035). Scale bars on all images are 200 µm. (**E**) Histology and immunohistochemistry images for zccs486 sample. Panels H&E, Ki-67, Ewing sarcoma tumour-specific markers CD99 and NKX2.2. (**F**) Alisertib drug response in 3D bioprinted patient tumouroids, Kruskal–Wallis with Dunn's Test; n.s. *P* values are indicated on corresponding plot. Source data are available online for this figure.

 © The Author(s)
